# Supplementary material for: Ethnic disparities in COVID-19 mortality and cardiovascular disease in England and Wales between 2020-2022
Source: Nat Commun. 2025 Jul 2;16:6059. doi: 10.1038/s41467-025-59951-4 (PMC12217090; doi:10.1038/s41467-025-59951-4)
Supplement: Supplementary file 1 — Supplementary Information [file 41467_2025_59951_MOESM1_ESM.docx]

## Supplementary Materials

**Table of contents**

[Supplementary Materials 1](#_Toc196919866)

**[Supplementary Figures 3](#_Toc196919867)**

[Supplementary Figure 1. Study flow chart in England data. 3](#_Toc196919868)

[Supplementary Figure 2. Study flow chart in Wales data 4](#_Toc196919869)

[Supplementary Figure 3. Representation of the three classification systems in the NHS England and the impact of using them. 5](#_Toc196919870)

[Supplementary Figure 4. Age-standardised incidence rates (per 100,000 population/year) of a) 28-day mortality and b) 30-day CVD in Wales among COVID-19 patients aged >=30 years and stratifying by ethnicity group. 6](#_Toc196919871)

[Supplementary figure 5. Age-standardised incidence rates (per 100,000 population/year) of 28-day mortality in a) men and b) women, and of 30-day CVD in c) men and d) women diagnosed with COVID-19 between 30 and 100 years old and across different ethnic groups in Wales by period of recorded COVID-19 diagnosis. 9](#_Toc196919872)

[Supplementary Figure 6. Adjusted hazard ratios of 28-day mortality of the 6 high-level ethnicity groups by months of recorded COVID-19 diagnosis in England, using White ethnicity as reference group, in a) men and b) women. Dot lines in 1 highlights the risk from the reference group. 10](#_Toc196919873)

[Supplementary Figure 7. Adjusted hazard ratios of 30-day CVD of the 6 high-level ethnicity groups by months of recorded COVID-19 diagnosis in England, using White ethnicity as reference group, in a) men and b) women. 11](#_Toc196919874)

[**Supplementary Tables 12**](#_Toc196919875)

[Supplementary Table 1. List of terms included in outcome Cardiovascular Disease (CVD) 12](#_Toc196919876)

[Supplementary Table 2. Mapping between NHS ethnicity codes to High-level ethnic groups. 13](#_Toc196919877)

[Supplementary Table 3. Baseline characteristics of individuals aged between 30 to 100 diagnosed with COVID-19 in Wales by 6 High-level ethnic groups. 14](#_Toc196919878)

[Supplementary Table 4. Baseline characteristics of individuals aged between 30 to 100 years diagnosed with COVID-19 in Wales by 10 ethnic groups. 15](#_Toc196919879)

[Supplementary Table 5. Baseline characteristics of individuals aged between 30 to 100 diagnosed with COVID-19 in England by 6 High-level ethnic groups 17](#_Toc196919880)

[Supplementary Table 6. Baseline characteristics of individuals aged between 30 to 100 years diagnosed with COVID-19 in England by 19 NHS ethnicity codes.. 19](#_Toc196919881)

[Supplementary Table 7. Number of events and age-standardised IR of in 28-day mortality and 30-day CVD (100,000 population/year) in Wales by sex and in each ethnic group from 6 High-level ethnic groups and 10 ethnic codes. 21](#_Toc196919882)

[Supplementary Table 8. Number of events and age-standardised IR of in 28-day mortality and 30-day CVD (100,000 population/year) in England by sex and in each ethnic group from 6 High-level ethnic groups, 19 NHS ethnicity codes and SNOMED-CT concepts.. 23](#_Toc196919883)

[Supplementary Table 9. Adjusted hazard ratios of 28-day mortality from individuals diagnosed with COVID-19 with diverse ethnic background in England, using White British women as reference group 28](#_Toc196919884)

[Supplementary Table 10. Adjusted hazard ratios of 30-day CVD from individuals diagnosed with COVID-19 with diverse ethnic background in England, using White British women as reference group 30](#_Toc196919885)

[Supplementary Table 11. Adjusted hazard ratios of 28-day mortality from individuals diagnosed with COVID-19 with diverse ethnic background in Wales, using White women as reference group 32](#_Toc196919886)

[Supplementary Table 12. Adjusted hazard ratios of 30-day CVD from individuals diagnosed with COVID-19 with diverse ethnic background in Wales, using White women as reference group 33](#_Toc196919887)

[Supplementary table 13. Number of events and age-standardised IR of in 28-day mortality and 30-day CVD (100,000 population/year) in Wales by sex and time of COVID-19 diagnosis in each 6 High-level from 6 High-level ethnic groups and 10 ethnic codes 35](#_Toc196919888)

[Supplementary Table 14. Number of events and age-standardised IR of in 28-day mortality and 30-day CVD (100,000 population/year) in England by sex and time of COVID-19 diagnosis in each 6 High-level categories and 19 NHS ethnicity codes 45](#_Toc196919889)

[Supplementary Table 15. Adjusted hazard ratios of 28-day mortality from a) men and b) women diagnosed with COVID-19 with diverse ethnic background by the period of COVID-19 diagnosis in England, using White British women as reference group 60](#_Toc196919890)

[Supplementary Table 16. Adjusted hazard ratios of 30-day CVD from a) men and b) women diagnosed with COVID-19 with diverse ethnic background by the period of COVID-19 diagnosis in England, using White British women as reference group 64](#_Toc196919891)

### Supplementary Figures


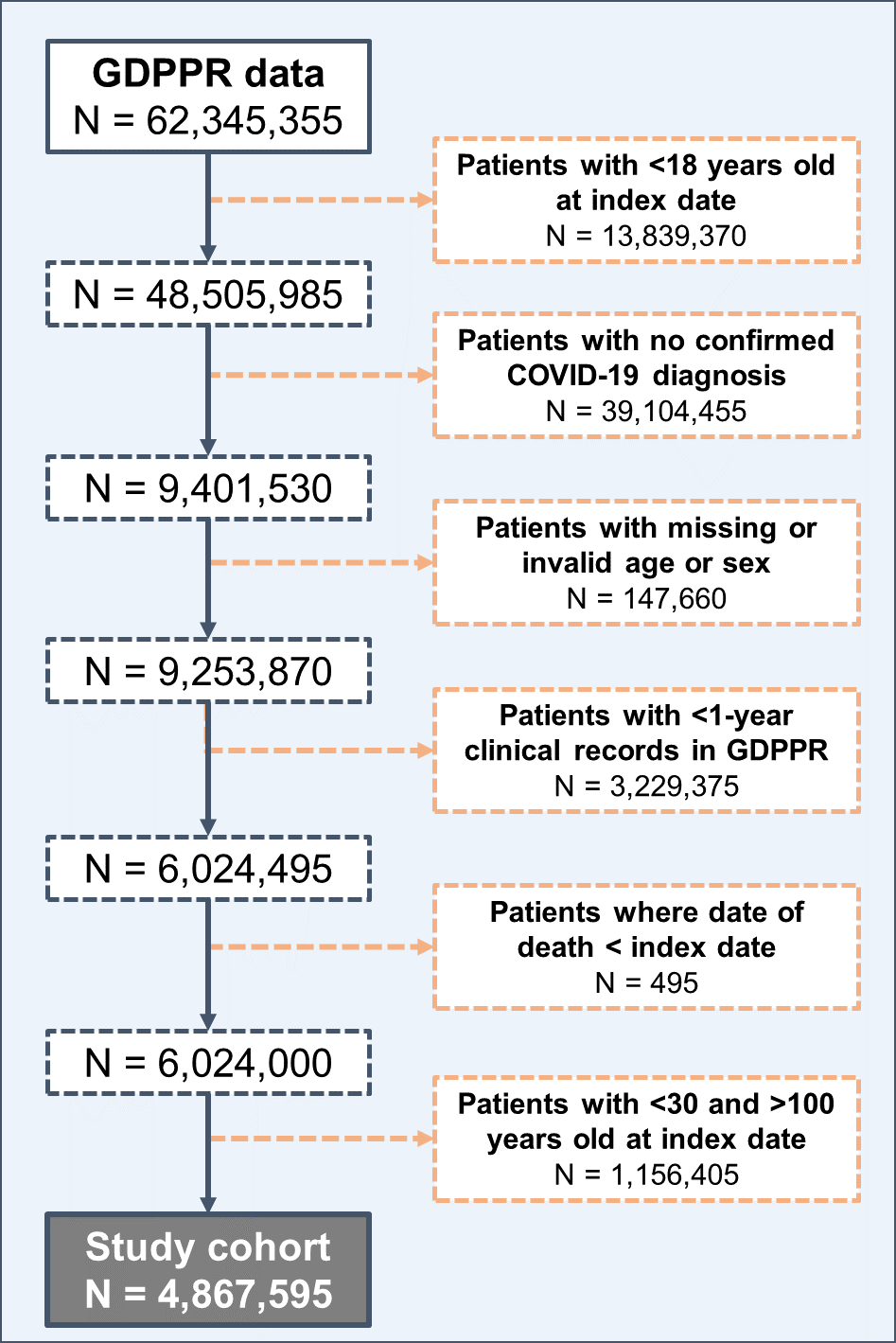


Supplementary Figure 1. Study flow chart in England data.
Patients were extracted from the General Practice Extraction Service Data for Pandemic Planning and Research (GDPPR).


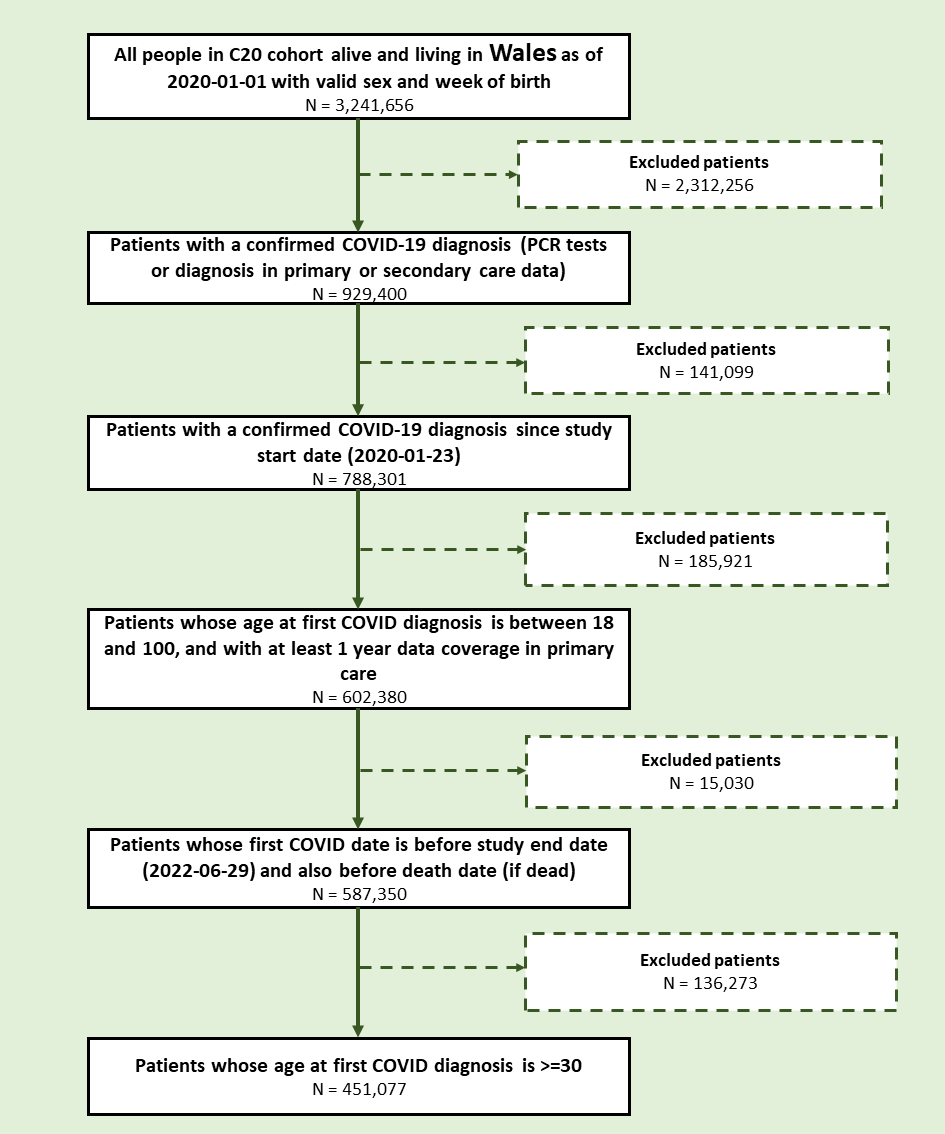


Supplementary Figure 2. Study flow chart in Wales data.
The C20 cohort is the Welsh COVID-19 e-cohort. DOI: 10.1136/bmjopen-2020-043010.

**
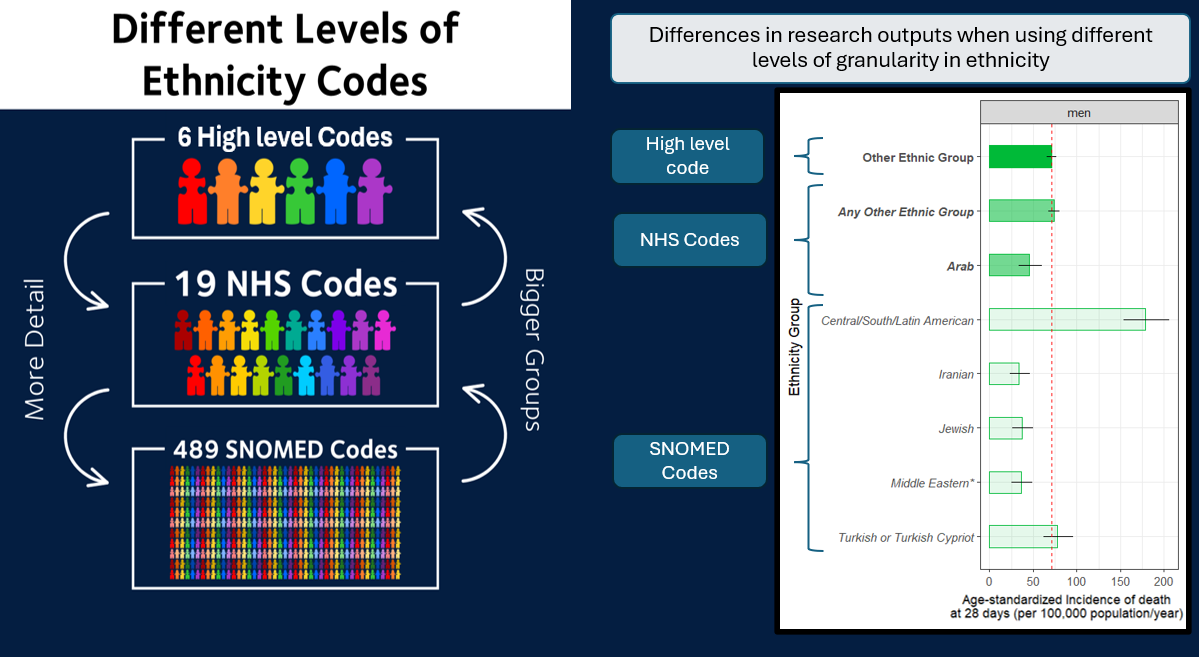
**

Supplementary Figure 3. Representation of the three classification systems in the NHS England and the impact of using them. High-level codes are the broader ethnicity categories whilst those SNOMED represent the most granular groups available. Use of NHS or SNOMED codes show differences across different ethnic groups that are masked when using the high-level codes. For instance, Arab men had lower incidence COVID-19 death compared to Other Ethnic Group, whilst incidence of COVID-19 death in the Central/South/Latin America population was more than double. The red dotted line indicates the age-standardised incidence rates of the Other Ethnic group.


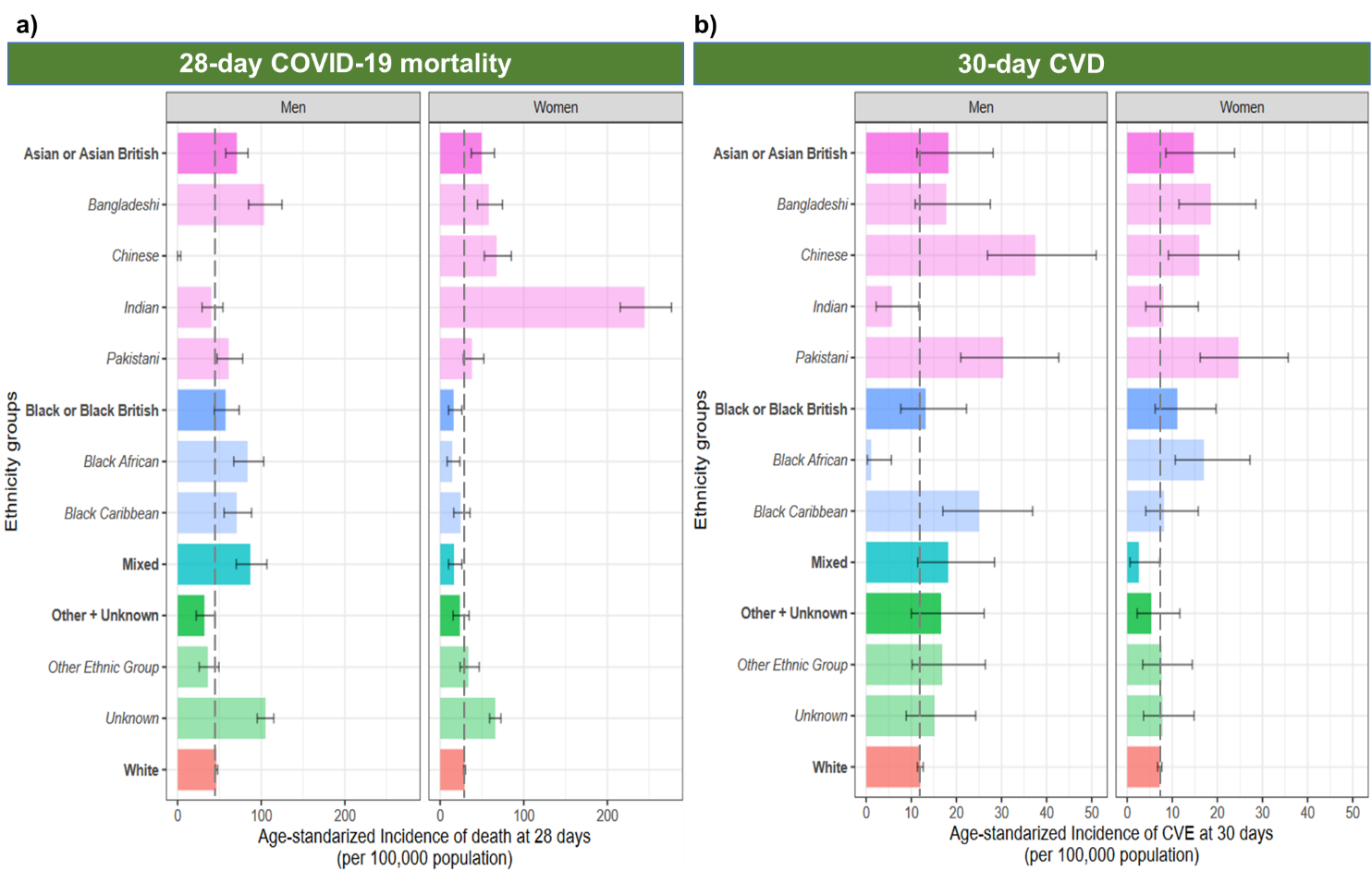


Supplementary Figure 4. Age-standardised incidence rates (per 100,000 population/year) of a) 28-day mortality and b) 30-day CVD in Wales among COVID-19 patients aged >=30 years and stratifying by ethnicity group.
Dark colours represent the 6 high-level groups and light colours correspond to 10 ethnicity sub-categories in Wales, which are denoted in bold and italics, respectively, in the Y axis. Vertical black dashed line marks the estimates from the White high-level group. To estimate the age-standardised incidence rates, age-specific incidence rates were calculated for 5-year age bands and then combined using the 2013 European Standard Population weights from 30 to 90+ age groups. Estimates are reported with their 95% confidence intervals. Abbreviation: CVD, cardiovascular disease; Middle eastern*, excluding Israeli, Iranian and Arab.


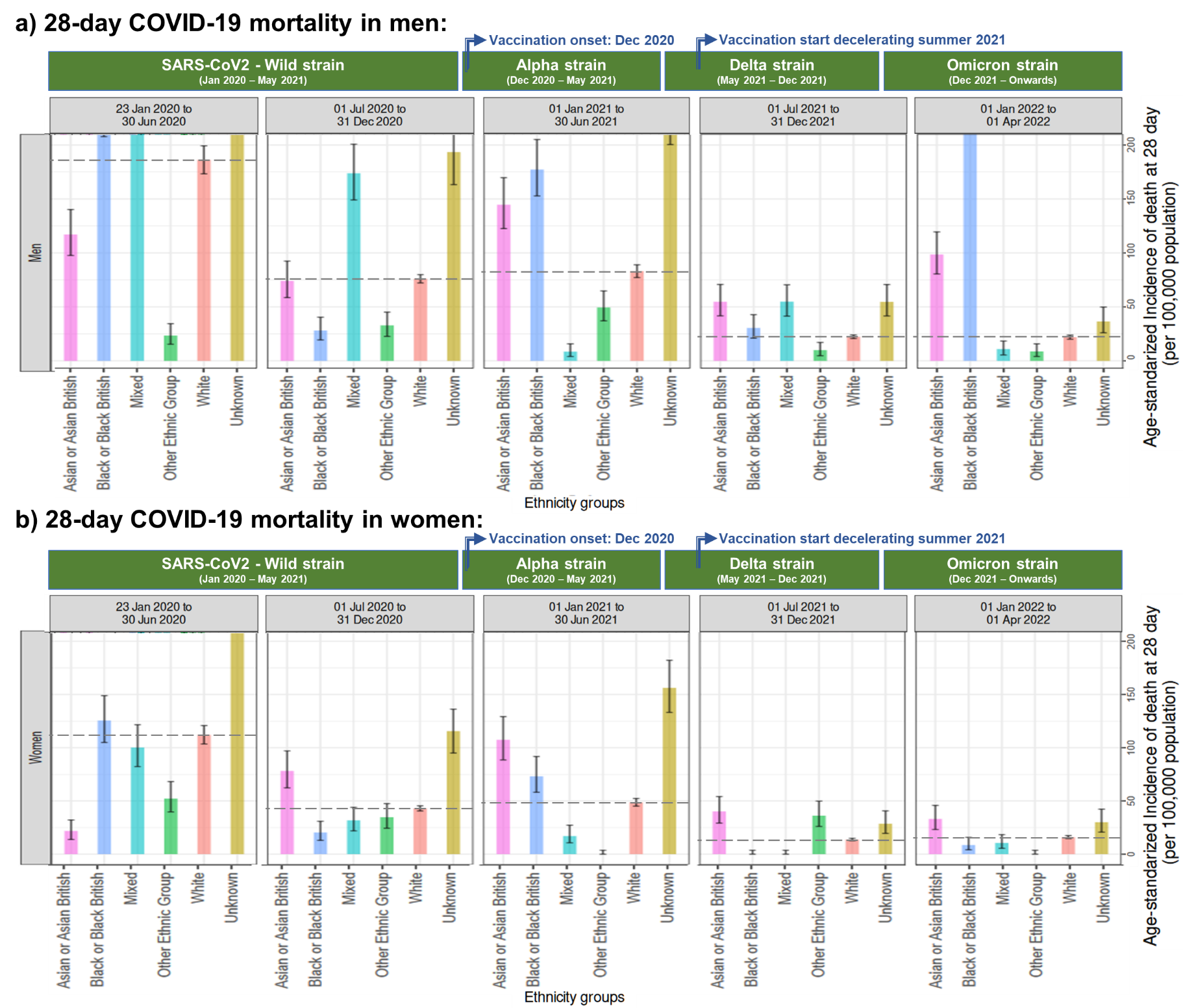


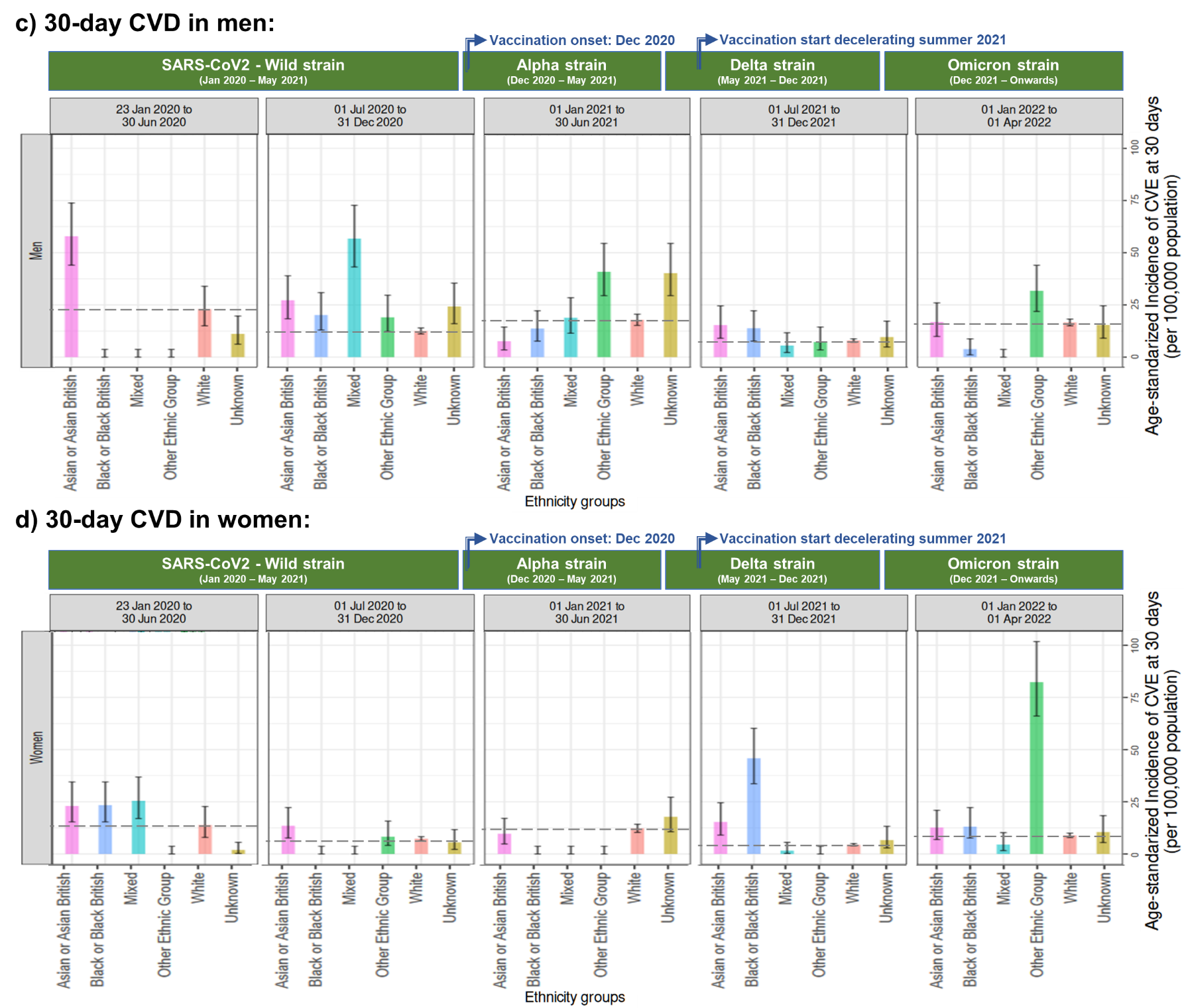


Supplementary figure 5. Age-standardised incidence rates (per 100,000 population/year) of 28-day mortality in a) men and b) women, and of 30-day CVD in c) men and d) women diagnosed with COVID-19 between 30 and 100 years old and across different ethnic groups in Wales by period of recorded COVID-19 diagnosis. Ethnic groups are the 6 high-level groups for Wales. Dotted horizontal black lines mark the estimates from the White high-level group. Important dates for contextualization such as the entrance of SARS-CoV2 variants and vaccination in the UK has been included. To estimate the age-standardised incidence rates, age-specific incidence rates were calculated for 5-year age bands and then combined using the 2013 European Standard Population weights from 30 to 90+ age groups. Estimates are reported with their 95% confidence intervals.


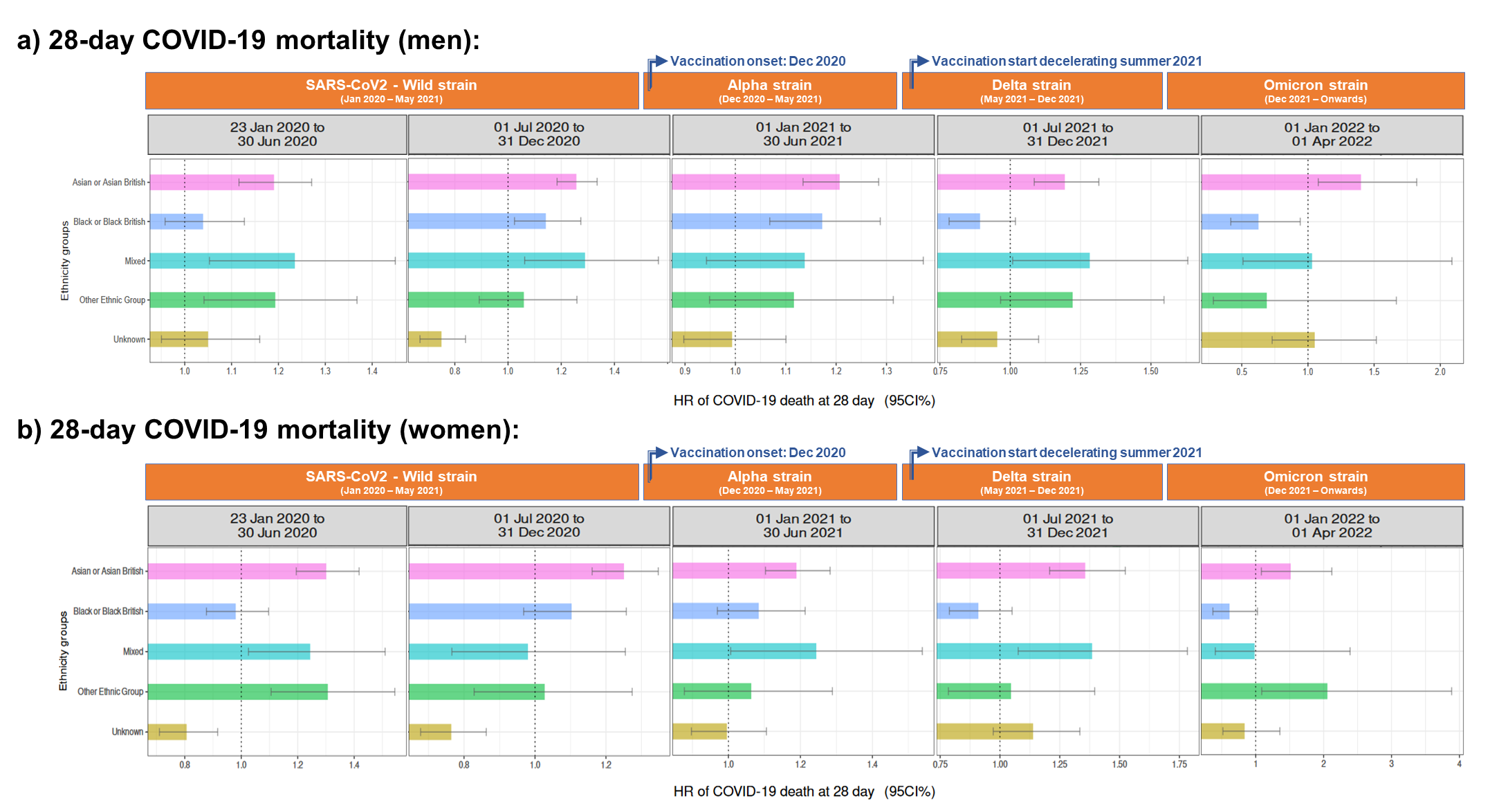


Supplementary Figure 6. Adjusted hazard ratios of 28-day mortality of the 6 high-level ethnicity groups by months of recorded COVID-19 diagnosis in England, using White ethnicity as reference group, in a) men and b) women. Dot lines in 1 highlights the risk from the reference group. Models were adjusted by age, ethnicity, deprivation index, vaccination status, geographic location in England, period of recorded COVID-19 diagnosis and comorbidities. Displayed hazard ratios belong to ethnicity coefficients and are reported with their 95% confidence intervals. Abbreviations: CI, confidence intervals; HR, hazard ratios.


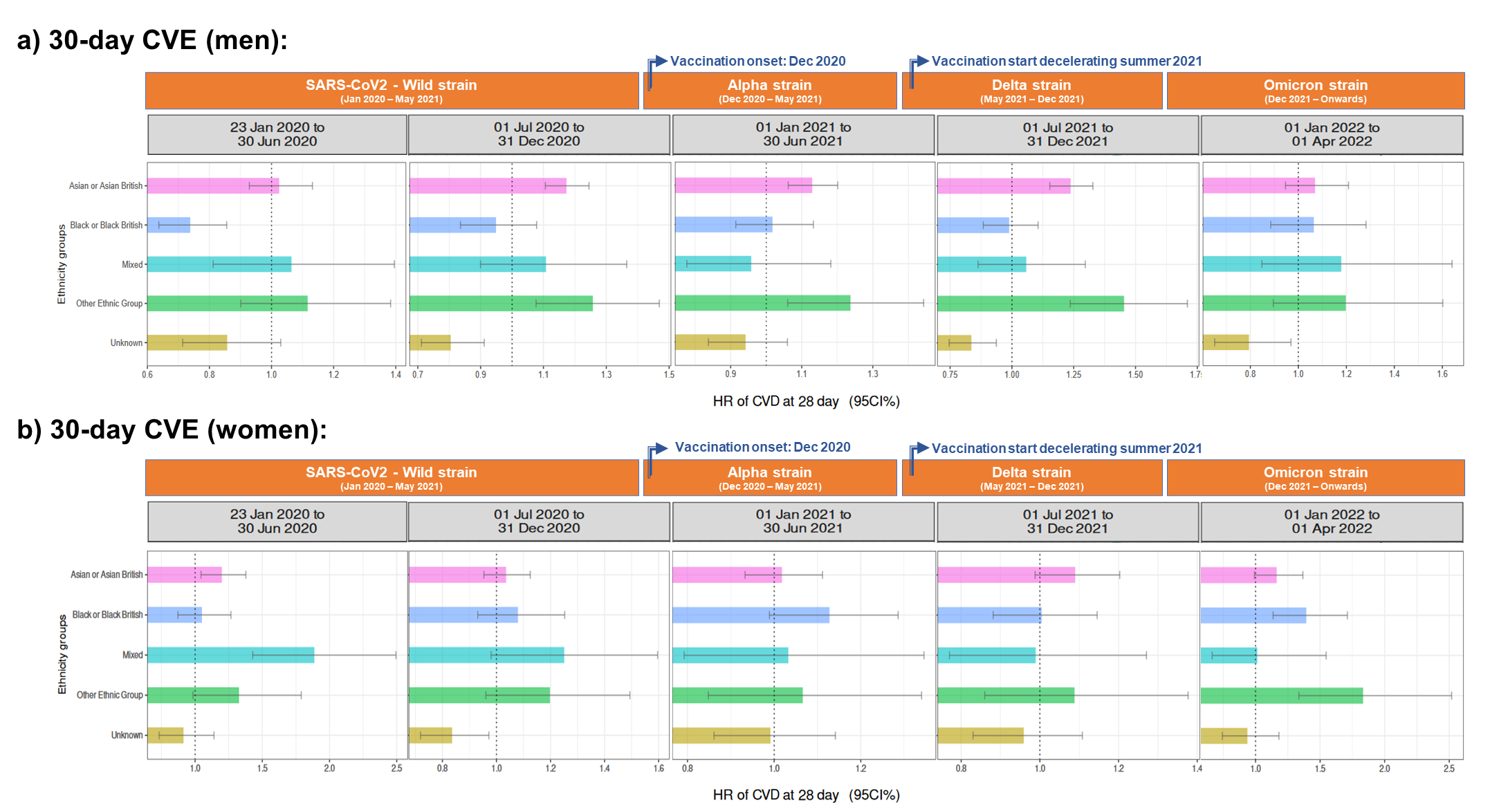


Supplementary Figure 7. Adjusted hazard ratios of 30-day CVD of the 6 high-level ethnicity groups by months of recorded COVID-19 diagnosis in England, using White ethnicity as reference group, in a) men and b) women. Dot lines in 1 highlights the risk from the reference group. Models were adjusted by age, ethnicity, deprivation index, vaccination status, geographic location in England, period of recorded COVID-19 diagnosis and comorbidities. Displayed hazard ratios belong to ethnicity coefficients and are reported with their 95% confidence intervals. Abbreviations: CI, confidence intervals; CVD, cardiovascular disease; HR, hazard ratios.

### Supplementary Tables

### Supplementary Table 1. List of terms included in outcome Cardiovascular Disease (CVD). Hypertension was not included.

| Abdominal aortic aneurysm |
| --- |
| Atrial fibrillation |
| Atrioventricular block complete |
| Atrioventricular block first degree |
| Atrioventricular block second degree |
| Bifascicular block |
| Branch block |
| Coronary heart disease not otherwise specified |
| Dilated cardiomyopathy |
| Heart failure |
| Hypertrophic cardiomyopathy |
| Intracerebral haemorrhage |
| Ischaemic stroke |
| Left bundle |
| Myocardial infraction |
| Other cardiomyopathy |
| Pericardial effusion non-inflammatory |
| Peripheral arterial disease |
| Primary pulmonary hypertension |
| Pulmonary embolism |
| Raynaud's syndrome |
| Rheumatic valve disease |
| Right bundle branch block |
| Secondary pulmonary hypertension |
| Sick sinus syndrome stable angina |
| Stroke (NOS) |
| Subarachnoid haemorrhage |
| Subdural hematoma non-traumatic |
| Supraventricular tachycardia |
| Transient ischaemic attack |
| Trifascicular block |
| Unstable angina |
| Venous thromboembolic disease (excluding Pulmonary Embolism) |
| Ventricular tachycardia |

### Supplementary Table 2. Mapping between NHS ethnicity codes to High-level ethnic groups.

| **High-level ethnic groups** | **NHS ethnicity codes** | |
| --- | --- | --- |
|  | **Short character abbreviation** | **Full code** |
| Asian or Asian British | 4 | Indian (prior 2001) |
|  | 5 | Pakistani (prior 2001) |
|  | 6 | Bangladeshi (prior 2001) |
|  | 7 | Chinese (prior 2001) |
|  | H | Indian |
|  | J | Pakistani |
|  | K | Bangladeshi |
|  | R | Chinese |
|  | L | Any other Asian background |
| Black or Black British | 1 | Black - Caribbean (prior 2001) |
|  | 2 | Black - African (prior 2001) |
|  | 3 | Black - Other (prior 2001) |
|  | M | Caribbean |
|  | N | African |
|  | P | Any other Black background |
| Mixed | D | White and Black Caribbean |
|  | E | White and Black African |
|  | F | White and Asian |
|  | G | Any other Mixed background |
| Other Ethnic Groups | 8 | Any Other Ethnic Group (prior 2001) |
|  | W | Arab |
|  | S | Any Other Ethnic Group |
| White | 0 | White (prior 2001) |
|  | A | British |
|  | B | Irish |
|  | C | Any other White background |
|  | T | Gypsy or Irish Traveller |
| Unknown | 9 | Not given |
|  | 99 | Not known (2013 onwards) |
|  | X | Not known (prior 2013) |
|  | Z | Not stated |

Supplementary Table 3. Baseline characteristics of individuals aged between 30 to 100 diagnosed with COVID-19 in Wales by 6 High-level ethnic groups. Variables were collected at the date of COVID-19 diagnosis except the study outcomes (i.e., 28-day mortality and 30-day CVD). Abbreviations: CVD, cardiovascular disease; IMD, index of multiple deprivation; n, number of individuals; SD, standard deviation.

| **High-level ethnic groups** | **Asian or Asian British** | **Black or Black British** | **Mixed** | **Other Ethnic Group** | **Unknown** | **White** |
| --- | --- | --- | --- | --- | --- | --- |
| **Variables** |  |  |  |  |  |  |
| **N (%)** | 11,082 | 3058* | 3067* | 3598* | 14,798 | 415,474 |
|  | 2.46% | 0.68% | 0.68% | 0.80% | 3.28% | 92.11% |
| **Women (%)** | 6061 (54.7) | 1632 (53.2) | 1756 (57.2) | 1868 (51.7) | 6300 (42.6) | 230457 (55.5) |
| **Age (mean (SD))** | 47.14 (13.04) | 45.34 (11.78) | 43.55 (11.57) | 44.85 (11.12) | 54.56 (16.38) | 51.30 (15.24) |
| **IMD quintile (%)** |  |  |  |  |  |  |
| **1 (most deprived)** | 3125 (30.5) | 1247 (44.6) | 897 (31.4) | 1105 (33.4) | 3161 (22.4) | 79818 (20.2) |
| **2** | 1676 (16.4) | 510 (18.3) | 538 (18.9) | 592 (17.9) | 3095 (21.9) | 83827 (21.3) |
| **3** | 1921 (18.8) | 421 (15.1) | 440 (15.4) | 519 (15.7) | 2547 (18.0) | 74398 (18.9) |
| **4** | 1365 (13.3) | 278 (10.0) | 421 (14.8) | 440 (13.3) | 2609 (18.5) | 75744 (19.2) |
| **5 (less deprived)** | 2147 (21.0) | 337 (12.1) | 558 (19.6) | 651 (19.7) | 2728 (19.3) | 80406 (20.4) |
| **Vaccination (%)** | 6415 (57.9) | 1573 (51.3) | 1806 (58.8) | 2023 (56.0) | 8924 (60.3) | 275636 (66.3) |
| **Pregnant (%)** | 159 (1.4) | 56 (1.8) | 56 (1.8) | 41 (1.1) | 47 (0.3) | 3383 (0.8) |
| **Atrial Fibrillation (%)** | 263 (2.4) | 34 (1.1) | 46 (1.5) | 57 (1.6) | 755 (5.1) | 18813 (4.5) |
| **Bipolar disorder (%)** | 37 (0.3) | 18 (0.6) | 31 (1.0) | <10 | 62 (0.4) | 3309 (0.8) |
| **Cancer (%)** | 725 (6.5) | 131 (4.3) | 181 (5.9) | 180 (5.0) | 1697 (11.5) | 50232 (12.1) |
| **Chronic Kidney Disease (%)** | 311 (2.8) | 74 (2.4) | 42 (1.4) | 51 (1.4) | 637 (4.3) | 15277 (3.7) |
| **Chronic obstructive pulmonary disease (%)** | 261 (2.4) | 31 (1.0) | 50 (1.6) | 53 (1.5) | 614 (4.1) | 18028 (4.3) |
| **Dementia (%)** | 130 (1.2) | 26 (0.8) | 27 (0.9) | 18 (0.5) | 580 (3.9) | 10320 (2.5) |
| **Depression (%)** | 1676 (15.1) | 514 (16.8) | 860 (28.0) | 564 (15.6) | 3013 (20.4) | 121718 (29.3) |
| **Diabetes (%)** | 1040 (9.4) | 202 (6.6) | 125 (4.1) | 173 (4.8) | 863 (5.8) | 25522 (6.1) |
| **Hypertension (%)** | 2266 (20.4) | 565 (18.4) | 368 (12.0) | 519 (14.4) | 3355 (22.7) | 93530 (22.5) |
| **Obesity (%)** | 2026 (18.3) | 543 (17.7) | 569 (18.5) | 619 (17.1) | 1985 (13.4) | 87404 (21.0) |
| **Osteoporosis (%)** | 281 (2.5) | 56 (1.8) | 63 (2.1) | 72 (2.0) | 687 (4.6) | 18896 (4.5) |
| **Rheumatoid Arthritis (%)** | 78 (0.7) | 20 (0.7) | 21 (0.7) | 18 (0.5) | 112 (0.8) | 4402 (1.1) |
| **Schizophrenia (%)** | 67 (0.6) | 41 (1.3) | 36 (1.2) | 25 (0.7) | 106 (0.7) | 3688 (0.9) |
| **Anti-hypertensive drugs (%)** | 23 (0.2) | <10 | <10 | 10 (0.3) | 33 (0.2) | 978 (0.2) |
| **Antipsychotic (%)** | 133 (1.2) | 50 (1.6) | 58 (1.9) | 56 (1.6) | 282 (1.9) | 9198 (2.2) |
| **Anti-coagulant drugs (%)** | 249 (2.2) | 46 (1.5) | 60 (2.0) | 49 (1.4) | 643 (4.3) | 18685 (4.5) |
| **Anti-diabetic drugs (%)** | 1516 (13.7) | 292 (9.5) | 185 (6.0) | 279 (7.7) | 971 (6.6) | 27965 (6.7) |
| **Anti-platelet drugs (%)** | 849 (7.7) | 143 (4.7) | 124 (4.0) | 185 (5.1) | 1189 (8.0) | 34720 (8.4) |
| **Statins (%)** | 2045 (18.5) | 309 (10.1) | 291 (9.5) | 429 (11.9) | 2517 (17.0) | 71293 (17.2) |
| **CVD history (ever) (%)** | 1028 (9.3) | 184 (6.0) | 166 (5.4) | 213 (5.9) | 1749 (11.8) | 52084 (12.5) |
| **CVD history (1-year prior) (%)** | 121 (1.1) | 18 (0.6) | 13 (0.4) | 12 (0.3) | 230 (1.6) | 5614 (1.4) |
| **28-days mortality (%)** | 191 (1.7) | 25 (0.8) | 25 (0.8) | 21 (0.6) | 791 (5.3) | 7877 (1.9) |
| **30-days CVD (%)** | 67 (0.6) | 14 (0.5) | 12 (0.4) | 13 (0.4) | 2244 (0.5) | 110 (0.7) |
| **Abbreviations:** *, excluding n<10; NA, non-available. | | | | | | |

Supplementary Table 4. Baseline characteristics of individuals aged between 30 to 100 years diagnosed with COVID-19 in Wales by 10 ethnic groups. Variables were collected at the date of COVID-19 diagnosis except the study outcomes (i.e., 28-day mortality and 30-day CVD). Abbreviations: CVD, cardiovascular disease; IMD, index of multiple deprivation; n, number of individuals; SD, standard deviation.

| **High-level ethnic groups** | **Asian/Asian British** | | | | **Black/Black British** | | **Mixed** | **Other Ethnic Groups** | **White** | **Unknown/ Not stated** |
| --- | --- | --- | --- | --- | --- | --- | --- | --- | --- | --- |
| **10 ethnicity codes** | **Indian** | **Pakistani** | **Bangladeshi** | **Chinese** | **African** | **Caribbean** |  |  |  |  |
|  |  |  |  |  |  |  |  |  |  |  |
| **Variables** |  |  |  |  |  |  |  |  |  |  |
| **N  (%)** | 2776* | 1958* | 2,930 | 594* | 2068* | 493* | 3067* | 6768* | 415,474 | 14,798 |
|  | 0.62% | 0.43% | 0.65% | 0.13% | 0.46% | 0.11% | 0.68% | 1.50% | 92.11% | 3.28% |
| **Women (%)** | 1419 (50.7) | 995 (50.4) | 1525 (52.0) | 417 (65.3) | 1137 (54.4) | 291 (52.7) | 1756 (57.2) | 3777 (55.8) | 230457 (55.5) | 6300 (42.6) |
| **Age (mean (SD))** | 46.04 (11.02) | 46.19 (12.69) | 49.97 (16.73) | 45.37 (11.98) | 44.31 (11.01) | 49.48 (14.03) | 43.55 (11.57) | 45.46 (10.76) | 51.30 (15.24) | 54.56 (16.38) |
| **IMD quintile (%)** |  |  |  |  |  |  |  |  |  |  |
| **1** | 611 (24.3) | 722 (38.0) | 859 (31.5) | 116 (20.1) | 885 (46.9) | 193 (37.1) | 897 (31.4) | 2091 (33.7) | 79818 (20.2) | 3161 (22.4) |
| **(most deprived)** |  |  |  |  |  |  |  |  |  |  |
| **2** | 406 (16.1) | 234 (12.3) | 452 (16.6) | 102 (17.7) | 342 (18.1) | 83 (16.0) | 538 (18.9) | 1159 (18.7) | 83827 (21.3) | 3095 (21.9) |
| **3** | 384 (15.3) | 347 (18.3) | 698 (25.6) | 76 (13.2) | 271 (14.4) | 100 (19.2) | 440 (15.4) | 985 (15.9) | 74398 (18.9) | 2547 (18.0) |
| **4** | 363 (14.4) | 180 (9.5) | 395 (14.5) | 96 (16.6) | 169 (9.0) | 67 (12.9) | 421 (14.8) | 813 (13.1) | 75744 (19.2) | 2609 (18.5) |
| **5** | 754 (29.9) | 416 (21.9) | 326 (11.9) | 187 (32.4) | 221 (11.7) | 77 (14.8) | 558 (19.6) | 1154 (18.6) | 80406 (20.4) | 2728 (19.3) |
| **(less deprived)** |  |  |  |  |  |  |  |  |  |  |
| **Vaccination (%)** | 1704 (60.9) | 975 (49.4) | 1793 (61.2) | 461 (72.1) | 1068 (51.1) | 295 (53.4) | 1806 (58.8) | 3715 (54.8) | 275636 (66.3) | 8924 (60.3) |
| **Pregnant (%)** | 35 (1.3) | 39 (2.0) | 41 (1.4) | 10 (1.6) | 48 (2.3) | <10 | 56 (1.8) | 80 (1.2) | 3383 (0.8) | 47 (0.3) |
| **Atrial Fibrillation (%)** | 18 (0.6) | 30 (1.5) | 174 (5.9) | 10 (1.6) | 21 (1.0) | <10 | 46 (1.5) | 93 (1.4) | 18813 (4.5) | 755 (5.1) |
| **Bipolar disorder (%)** | <10 | <10 | 18 (0.6) | <10 | <10 | <10 | 31 (1.0) | 17 (0.3) | 3309 (0.8) | 62 (0.4) |
| **Cancer (%)** | 110 (3.9) | 91 (4.6) | 343 (11.7) | 29 (4.5) | 76 (3.6) | 31 (5.6) | 181 (5.9) | 356 (5.3) | 50232 (12.1) | 1697 (11.5) |
| **Chronic Kidney Disease (%)** | 36 (1.3) | 62 (3.1) | 162 (5.5) | <10 | 37 (1.8) | 25 (4.5) | 42 (1.4) | 107 (1.6) | 15277 (3.7) | 637 (4.3) |
| **Chronic obstructive pulmonary disease (%)** | 15 (0.5) | 46 (2.3) | 178 (6.1) | <10 | 21 (1.0) | 5 (0.9) | 50 (1.6) | 78 (1.2) | 18028 (4.3) | 614 (4.1) |
| **Dementia (%)** | 14 (0.5) | 14 (0.7) | 95 (3.2) | 0 (0.0) | 14 (0.7) | 11 (2.0) | 27 (0.9) | 26 (0.4) | 10320 (2.5) | 580 (3.9) |
| **Depression (%)** | 269 (9.6) | 374 (18.9) | 677 (23.1) | 63 (9.9) | 298 (14.3) | 129 (23.4) | 860 (28.0) | 944 (13.9) | 121718 (29.3) | 3013 (20.4) |
| **Diabetes (%)** | 229 (8.2) | 273 (13.8) | 334 (11.4) | 29 (4.5) | 129 (6.2) | 39 (7.1) | 125 (4.1) | 382 (5.6) | 25522 (6.1) | 863 (5.8) |
| **Hypertension (%)** | 506 (18.1) | 394 (19.9) | 756 (25.8) | 66 (10.3) | 360 (17.2) | 130 (23.6) | 368 (12.0) | 1138 (16.8) | 93530 (22.5) | 3355 (22.7) |
| **Obesity (%)** | 384 (13.7) | 405 (20.5) | 801 (27.3) | 74 (11.6) | 352 (16.8) | 121 (21.9) | 569 (18.5) | 1051 (15.5) | 87404 (21.0) | 1985 (13.4) |
| **Osteoporosis (%)** | 48 (1.7) | 47 (2.4) | 144 (4.9) | <10 | 38 (1.8) | 12 (2.2) | 63 (2.1) | 112 (1.7) | 18896 (4.5) | 687 (4.6) |
| **Rheumatoid Arthritis (%)** | 14 (0.5) | 20 (1.0) | 35 (1.2) | <10 | 11 (0.5) | <10 | 21 (0.7) | 28 (0.4) | 4402 (1.1) | 112 (0.8) |
| **Schizophrenia (%)** | 10 (0.4) | 15 (0.8) | 28 (1.0) | <10 | 22 (1.1) | 13 (2.4) | 36 (1.2) | 42 (0.6) | 3688 (0.9) | 106 (0.7) |
| **Anti-hypertensive drugs (%)** | <10 | <10 | 11 (0.4) | <10 | <10 | <10 | <10 | 13 (0.2) | 978 (0.2) | 33 (0.2) |
| **Antipsychotic (%)** | 15 (0.5) | 19 (1.0) | 75 (2.6) | <10 | 34 (1.6) | 10 (1.8) | 58 (1.9) | 82 (1.2) | 9198 (2.2) | 282 (1.9) |
| **Anti-coagulant drugs (%)** | 22 (0.8) | 30 (1.5) | 162 (5.5) | 10 (1.6) | 30 (1.4) | <10 | 60 (2.0) | 81 (1.2) | 18685 (4.5) | 643 (4.3) |
| **Anti-diabetic drugs (%)** | 395 (14.1) | 365 (18.5) | 411 (14.0) | 36 (5.6) | 202 (9.7) | 52 (9.4) | 185 (6.0) | 626 (9.2) | 27965 (6.7) | 971 (6.6) |
| **Anti-platelet drugs (%)** | 153 (5.5) | 191 (9.7) | 332 (11.3) | 21 (3.3) | 80 (3.8) | 38 (6.9) | 124 (4.0) | 362 (5.3) | 34720 (8.4) | 1189 (8.0) |
| **Statins (%)** | 485 (17.3) | 445 (22.5) | 628 (21.4) | 63 (9.9) | 184 (8.8) | 83 (15.0) | 291 (9.5) | 895 (13.2) | 71293 (17.2) | 2517 (17.0) |
| **CVD history (ever) (%)** | 141 (5.0) | 182 (9.2) | 524 (17.9) | 29 (4.5) | 100 (4.8) | 55 (10.0) | 166 (5.4) | 394 (5.8) | 52084 (12.5) | 1749 (11.8) |
| **CVD history (1-year prior) (%)** | <10 | 18 (0.9) | 75 (2.6) | <10 | 11 (0.5) | <10 | 13 (0.4) | 33 (0.5) | 5614 (1.4) | 230 (1.6) |
| **28-days mortality (%)** | 19 (0.7) | 22 (1.1) | 121 (4.1) | <11 | 13 (0.6) | <10 | 25 (0.8) | 50 (0.7) | 7877 (1.9) | 791 (5.3) |
| **30-days CVD (%)** | <10 | 19 (1.0) | 26 (0.9) | <13 | <10 | <10 | 12 (0.4) | 25 (0.4) | 2244 (0.5) | 110 (0.7) |
| **Abbreviations:** *, excluding n<10; NA, non-available. | | | | | | | | | | |

Supplementary Table 5. Baseline characteristics of individuals aged between 30 to 100 diagnosed with COVID-19 in England by 6 High-level ethnic groups. Variables were collected at the date of COVID-19 diagnosis except the study outcomes (i.e., 28-day mortality and 30-day CVD). Abbreviations: CVD, cardiovascular disease; IMD, index of multiple deprivation; n, number of individuals; SD, standard deviation.

| **High-level ethnic groups**  **Variables** | **Asian or Asian British** | **Black or Black British** | **Mixed** | **Other Ethnic Group** | **Unknown** | **White** |
| --- | --- | --- | --- | --- | --- | --- |
| **N (%)** | 389,845  (8.0%) | 154,185  (3.2%) | 61,595  (1.3%) | 61,590  (1.3%) | 158,710  (3.3%) | 4,041,670  (83.0%) |
| **Women (%)** | 227,015 (58.2) | 97,770 (63.4) | 39,500 (64.1) | 36,660 (59.5) | 91,650 (57.7) | 2,430,150 (60.1) |
| **Age (mean (SD))** | 48.51 (13.23) | 49.36 (13.11) | 45.49 (12.36) | 47.53 (13.02) | 49.59 (14.88) | 51.94 (15.39) |
| **IMD quintile (%)** |  |  |  |  |  |  |
| **1 (most deprived)** | 114,380 (29.4) | 57,780 (37.5) | 17,110 (27.8) | 17,595 (28.6) | 30,265 (19.1) | 798,820 (19.7) |
| **2** | 103,760 (26.6) | 48,125 (31.2) | 15,425 (25.0) | 16,405 (26.6) | 31,005 (19.5) | 782,910 (19.4) |
| **3** | 74,010 (19.0) | 25,350 (16.4) | 11,645 (18.9) | 11,395 (18.5) | 31,720 (20.0) | 808,150 (20.0) |
| **4** | 53,600 (13.7) | 14,065 (9.1) | 9,405 (15.3) | 8,890 (14.4) | 32,525 (20.5) | 838,290 (20.7) |
| **5 (less deprived)** | 43,935 (11.3) | 8,765 (5.7) | 7,975 (12.9) | 7,260 (11.8) | 33,125 (20.9) | 811,170 (20.1) |
| **Unknown** | 160 (0.0) | 100 (0.1) | 35 (0.1) | 45 (0.1) | 70 (0.0) | 2,330 (0.1) |
| **Vaccination (%)** | 188,570 (48.4) | 66,455 (43.1) | 31,325 (50.9) | 30,345 (49.3) | 100,650 (63.4) | 2,606,080 (64.5) |
| **Pregnant (%)** | 2,700 (0.7) | 845 (0.5) | 435 (0.7) | 405 (0.7) | 535 (0.3) | 14,395 (0.4) |
| **Smokers (%)** | 125,230 (32.1) | 57,740 (37.4) | 31,930 (51.8) | 29,530 (47.9) | 89,885 (56.6) | 2,436,135 (60.3) |
| **Alcohol problems (%)** | 14,790 (3.8) | 6,315 (4.1) | 2,825 (4.6) | 2,605 (4.2) | 5,905 (3.7) | 216,150 (5.3) |
| **Atrial Fibrillation (%)** | 5,275 (1.4) | 2,340 (1.5) | 810 (1.3) | 1,230 (2.0) | 4,895 (3.1) | 169,825 (4.2) |
| **Bipolar disorder (%)** | 1,545 (0.4) | 800 (0.5) | 480 (0.8) | 315 (0.5) | 725 (0.5) | 23,830 (0.6) |
| **Cancer (%)** | 111,495 (28.6) | 49,780 (32.3) | 21,145 (34.3) | 19,405 (31.5) | 54,510 (34.3) | 1,358,970 (33.6) |
| **Chronic Kidney Disease (%)** | 24,505 (6.3) | 13,010 (8.4) | 2,800 (4.5) | 2,550 (4.1) | 8,575 (5.4) | 273,415 (6.8) |
| **Chronic obstructive pulmonary disease (%)** | 4,520 (1.2) | 1,600 (1.0) | 650 (1.1) | 875 (1.4) | 3,475 (2.2) | 141,220 (3.5) |
| **Dementia (%)** | 3,505 (0.9) | 2,465 (1.6) | 570 (0.9) | 740 (1.2) | 3,550 (2.2) | 110,670 (2.7) |
| **Depression (%)** | 8,065 (2.1) | 2,925 (1.9) | 1,945 (3.2) | 1,660 (2.7) | 7,075 (4.5) | 149,050 (3.7) |
| **Diabetes (%)** | 96,155 (24.7) | 28,205 (18.3) | 7,015 (11.4) | 7,890 (12.8) | 13,930 (8.8) | 382,840 (9.5) |
| **Hypertension (%)** | 107,160 (27.5) | 54,625 (35.4) | 12,700 (20.6) | 12,665 (20.6) | 33,435 (21.1) | 972,200 (24.1) |
| **Obesity (%)** | 42,295 (10.8) | 21,610 (14.0) | 6,290 (10.2) | 6,155 (10.0) | 16,065 (10.1) | 420,235 (10.4) |
| **Osteoporosis (%)** | 6,765 (1.7) | 1,400 (0.9) | 605 (1.0) | 1,060 (1.7) | 3,280 (2.1) | 110,045 (2.7) |
| **Rheumatoid Arthritis (%)** | 5,700 (1.5) | 1,435 (0.9) | 605 (1.0) | 580 (0.9) | 1,535 (1.0) | 48,220 (1.2) |
| **Schizophrenia (%)** | 2,595 (0.7) | 2,060 (1.3) | 625 (1.0) | 365 (0.6) | 570 (0.4) | 15,865 (0.4) |
| **Anti-hypertensive drugs (%)** | 905 (0.2) | 950 (0.6) | 160 (0.3) | 110 (0.2) | 240 (0.2) | 7,230 (0.2) |
| **Antipsychotic (%)** | 745 (0.2) | 440 (0.3) | 190 (0.3) | 170 (0.3) | 495 (0.3) | 13,685 (0.3) |
| **Anti-coagulant drugs (%)** | 6,305 (1.6) | 3,430 (2.2) | 1,075 (1.7) | 1,350 (2.2) | 5,265 (3.3) | 182,265 (4.5) |
| **Anti-diabetic drugs (%)** | 79,380 (20.4) | 21,255 (13.8) | 5,315 (8.6) | 6,245 (10.1) | 9,950 (6.3) | 271,820 (6.7) |
| **Anti-platelet drugs (%)** | 37,740 (9.7) | 9,285 (6.0) | 2,810 (4.6) | 4,060 (6.6) | 9,390 (5.9) | 302,310 (7.5) |
| **Statins (%)** | 107,640 (27.6) | 26,435 (17.1) | 7,825 (12.7) | 10,920 (17.7) | 24,225 (15.3) | 757,895 (18.8) |
| **CVD history (ever) (%)** | 51,515 (13.2) | 17,060 (11.1) | 5,350 (8.7) | 6,900 (11.2) | 19,365 (12.2) | 623,715 (15.4) |
| **CVD history (1-year prior) (%)** | 16,675 (4.3) | 5,895 (3.8) | 1,705 (2.8) | 2,345 (3.8) | 6,290 (4.0) | 226,895 (5.6) |
| **28-days mortality (%)** | 7,120 (1.8) | 3,110 (2.0) | 770 (1.3) | 975 (1.6) | 2,370 (1.5) | 84,280 (2.1) |
| **30-days CVD (%)** | 6,945 (1.8) | 2,315 (1.5) | 625 (1.0) | 940 (1.5) | 1,790 (1.1) | 66,290 (1.6) |

Supplementary Table 6. Baseline characteristics of individuals aged between 30 to 100 years diagnosed with COVID-19 in England by 19 NHS ethnicity codes. Variables were collected at the date of COVID-19 diagnosis except the study outcomes (i.e., 28-day mortality and 30-day CVD). Abbreviations: CVD, cardiovascular disease; IMD, index of multiple deprivation; n, number of individuals; SD, standard deviation.

| **High-level ethnic groups** | **Asian/Asian British** | | | | | **Black/Black British** | | | **Mixed** | | | | **Other Ethnic Groups** | | **White** | | | | **Unknown/ Not stated** |
| --- | --- | --- | --- | --- | --- | --- | --- | --- | --- | --- | --- | --- | --- | --- | --- | --- | --- | --- | --- |
| **NHS ethnicity codes**  **Variables** | **Indian** | **Pakista-ni** | **Bangla-deshi** | **Chinese** | **Any other Asian back-**  **ground** | **African** | **Cari-bbean** | **Any other Black back-**  **ground** | **White and Black Cari-bbean** | **White and Black African** | **White and Asian** | **Any other Mixed back-ground** | **Arab** | **Any Other Ethnic Group** | **British** | **Irish** | **Gypsy or Irish Trave-ller** | **Any other White back-ground** |  |
| **N (%)** | 146,305  (3.0%) | 112,675  (2.3%) | 38,795  (0.8%) | 11,630  (0.2%) | 80,440  (1.7%) | 80,785  (1.7%) | 48,890  (1.0%) | 24,510  (0.5%) | 16,850  (0.3%) | 11,520  (0.2%) | 10,845  (0.2%) | 22,380  (0.5%) | 4,330  (0.1%) | 57,260  (1.2%) | 3,611,735  (74.2%) | 31,105  (0.6%) | 2,655  (0.1%) | 396,175  (8.1%) | 158,710  (3.3%) |
| **Women (%)** | 83,275 (56.9) | 65,325 (58.0) | 22,160 (57.1) | 7,840 (67.4) | 48,420 (60.2) | 51,540 (63.8) | 30,990 (63.4) | 15,240 (62.2) | 11,075 (65.7) | 7,395 (64.2) | 6,695 (61.7) | 14,335 (64.1) | 2,310 (53.3) | 34,355 (60.0) | 2,154,125 (59.6) | 17,625 (56.7) | 1,620 (61.0) | 256,780 (64.8) | 91,650 (57.7) |
| **Age (mean (SD))** | 49.70 (13.65) | 47.68 (13.31) | 46.67 (12.56) | 48.02 (13.39) | 48.46 (12.41) | 47.52 (11.30) | 53.31 (15.27) | 47.52 (12.23) | 45.49 (13.58) | 46.19 (11.21) | 45.60 (12.12) | 45.08 (12.06) | 47.07 (12.49) | 47.57 (13.06) | 52.33 (15.48) | 55.70 (16.80) | 47.37 (13.12) | 48.08 (13.84) | 49.59 (14.88) |
| **IMD quintile (%)** |  |  |  |  |  |  |  |  |  |  |  |  |  |  |  |  |  |  |  |
| **1  (most deprived)** | 25,585 (17.5) | 52,780 (46.8) | 16,005 (41.2) | 2,005 (17.2) | 18,010 (22.4) | 31,875 (39.5) | 17,280 (35.3) | 8,620 (35.1) | 5,540 (32.9) | 3,715 (32.2) | 2,200 (20.3) | 5,650 (25.3) | 1,595 (36.8) | 16,010 (28.0) | 707,960 (19.6) | 5,685 (18.2) | 600 (22.6) | 84,575 (21.4) | 30,265 (19.1) |
| **2** | 37,750 (25.8) | 28,350 (25.2) | 13,025 (33.6) | 2,440 (21.0) | 22,200 (27.6) | 24,920 (30.8) | 15,725 (32.2) | 7,480 (30.5) | 4,525 (26.9) | 2,865 (24.9) | 2,325 (21.4) | 5,715 (25.6) | 1,085 (25.1) | 15,315 (26.7) | 684,560 (19.0) | 6,900 (22.2) | 655 (24.7) | 90,795 (22.9) | 31,005 (19.5) |
| **3** | 33,055 (22.6) | 15,595 (13.8) | 5,375 (13.9) | 2,255 (19.4) | 17,725 (22.0) | 12,270 (15.2) | 8,905 (18.2) | 4,180 (17.1) | 3,045 (18.1) | 2,085 (18.1) | 2,180 (20.1) | 4,330 (19.3) | 750 (17.3) | 10,645 (18.6) | 720,330 (19.9) | 6,650 (21.4) | 595 (22.4) | 80,575 (20.3) | 31,720 (20.0) |
| **4** | 26,160 (17.9) | 9,545 (8.5) | 2,800 (7.2) | 2,270 (19.5) | 12,825 (15.9) | 7,045 (8.7) | 4,455 (9.1) | 2,570 (10.5) | 2,180 (12.9) | 1,580 (13.7) | 2,065 (19.0) | 3,580 (16.0) | 565 (13.0) | 8,325 (14.5) | 758,775 (21.0) | 6,080 (19.5) | 465 (17.5) | 72,975 (18.4) | 32,525 (20.5) |
| **5 (less deprived)** | 23,700 (16.2) | 6,370 (5.7) | 1,575 (4.1) | 2,650 (22.8) | 9,635 (12.0) | 4,630 (5.7) | 2,490 (5.1) | 1,640 (6.7) | 1,550 (9.2) | 1,265 (11.0) | 2,065 (19.0) | 3,095 (13.8) | 335 (7.7) | 6,925 (12.1) | 738,045 (20.4) | 5,770 (18.6) | 340 (12.8) | 67,015 (16.9) | 33,125 (20.9) |
| **Unknown** | 55 (0.0) | 35 (0.0) | 15 (0.0) | <10 (<0.1) | 45 (0.1) | 45 (0.1) | 35 (0.1) | 20 (0.1) | <10 (<0.1) | 10 (0.1) | <10 (<0.1) | 10 (0.0) | <10 (<0.2) | 40 (0.1) | 2,065 (0.1) | 20 (0.1) | <10 (<0.4) | 240 (0.1) | 70 (0.0) |
| **Vaccination (%)** | 79,155 (54.1) | 42,930 (38.1) | 16,195 (41.7) | 7,870 (67.7) | 42,425 (52.7) | 37,985 (47.0) | 18,205 (37.2) | 10,265 (41.9) | 7,340 (43.6) | 5,895 (51.2) | 6,255 (57.7) | 11,840 (52.9) | 1,915 (44.2) | 28,435 (49.7) | 2,361,025 (65.4) | 19,210 (61.8) | 1,250 (47.1) | 224,590 (56.7) | 100,650 (63.4) |
| **Pregnant (%)** | 805 (0.6) | 1,030 (0.9) | 355 (0.9) | 55 (0.5) | 455 (0.6) | 550 (0.7) | 170 (0.3) | 125 (0.5) | 120 (0.7) | 65 (0.6) | 90 (0.8) | 160 (0.7) | 45 (1.0) | 360 (0.6) | 12,260 (0.3) | 125 (0.4) | 25 (0.9) | 1,990 (0.5) | 535 (0.3) |
| **Smokers (%)** | 41,685 (28.5) | 38,090 (33.8) | 15,035 (38.8) | 3,960 (34.0) | 26,460 (32.9) | 21,500 (26.6) | 24,930 (51.0) | 11,310 (46.1) | 10,270 (60.9) | 4,410 (38.3) | 5,180 (47.8) | 12,065 (53.9) | 1,725 (39.8) | 27,810 (48.6) | 2,179,190 (60.3) | 19,695 (63.3) | 1,510 (56.9) | 235,745 (59.5) | 89,885 (56.6) |
| **Alcohol problems (%)** | 6,435 (4.4) | 4,245 (3.8) | 830 (2.1) | 345 (3.0) | 2,935 (3.6) | 3,150 (3.9) | 2,095 (4.3) | 1,070 (4.4) | 820 (4.9) | 535 (4.6) | 475 (4.4) | 995 (4.4) | 195 (4.5) | 2,410 (4.2) | 194,400 (5.4) | 2,435 (7.8) | 155 (5.8) | 19,160 (4.8) | 5,905 (3.7) |
| **Atrial Fibrillation (%)** | 2,130 (1.5) | 1,535 (1.4) | 345 (0.9) | 185 (1.6) | 1,080 (1.3) | 840 (1.0) | 1,170 (2.4) | 335 (1.4) | 240 (1.4) | 135 (1.2) | 135 (1.2) | 305 (1.4) | 70 (1.6) | 1,160 (2.0) | 156,575 (4.3) | 2,025 (6.5) | 60 (2.3) | 11,160 (2.8) | 4,895 (3.1) |
| **Bipolar disorder (%)** | 510 (0.3) | 545 (0.5) | 180 (0.5) | 40 (0.3) | 265 (0.3) | 325 (0.4) | 300 (0.6) | 175 (0.7) | 150 (0.9) | 75 (0.7) | 75 (0.7) | 180 (0.8) | 15 (0.3) | 300 (0.5) | 21,470 (0.6) | 275 (0.9) | 15 (0.6) | 2,070 (0.5) | 725 (0.5) |
| **Cancer (%)** | 43,430 (29.7) | 29,145 (25.9) | 8,640 (22.3) | 4,710 (40.5) | 25,570 (31.8) | 26,435 (32.7) | 15,530 (31.8) | 7,815 (31.9) | 5,800 (34.4) | 4,010 (34.8) | 3,670 (33.8) | 7,665 (34.2) | 1,235 (28.5) | 18,175 (31.7) | 1,197,210 (33.1) | 9,380 (30.2) | 1,005 (37.9) | 151,380 (38.2) | 54,510 (34.3) |
| **Chronic Kidney Disease (%)** | 9,410 (6.4) | 7,905 (7.0) | 2,790 (7.2) | 455 (3.9) | 3,945 (4.9) | 5,155 (6.4) | 6,180 (12.6) | 1,680 (6.9) | 1,055 (6.3) | 565 (4.9) | 400 (3.7) | 775 (3.5) | 150 (3.5) | 2,400 (4.2) | 253,365 (7.0) | 2,745 (8.8) | 110 (4.1) | 17,195 (4.3) | 8,575 (5.4) |
| **Chronic obstructive pulmonary disease (%)** | 1,440 (1.0) | 1,615 (1.4) | 615 (1.6) | 70 (0.6) | 780 (1.0) | 450 (0.6) | 905 (1.9) | 240 (1.0) | 210 (1.2) | 95 (0.8) | 115 (1.1) | 230 (1.0) | 55 (1.3) | 815 (1.4) | 130,770 (3.6) | 1,900 (6.1) | 60 (2.3) | 8,490 (2.1) | 3,475 (2.2) |
| **Dementia (%)** | 1,405 (1.0) | 980 (0.9) | 400 (1.0) | 120 (1.0) | 595 (0.7) | 540 (0.7) | 1,675 (3.4) | 250 (1.0) | 260 (1.5) | 55 (0.5) | 90 (0.8) | 160 (0.7) | 30 (0.7) | 710 (1.2) | 103,145 (2.9) | 1,545 (5.0) | 15 (0.6) | 5,970 (1.5) | 3,550 (2.2) |
| **Depression (%)** | 2,915 (2.0) | 2,725 (2.4) | 845 (2.2) | 140 (1.2) | 1,440 (1.8) | 1,220 (1.5) | 1,105 (2.3) | 600 (2.4) | 665 (3.9) | 260 (2.3) | 300 (2.8) | 720 (3.2) | 120 (2.8) | 1,540 (2.7) | 137,680 (3.8) | 1,005 (3.2) | 75 (2.8) | 10,290 (2.6) | 7,075 (4.5) |
| **Diabetes (%)** | 34,870 (23.8) | 29,650 (26.3) | 12,550 (32.3) | 1,285 (11.0) | 17,800 (22.1) | 14,115 (17.5) | 10,570 (21.6) | 3,525 (14.4) | 1,835 (10.9) | 1,540 (13.4) | 1,350 (12.4) | 2,290 (10.2) | 750 (17.3) | 7,140 (12.5) | 347,390 (9.6) | 3,200 (10.3) | 275 (10.4) | 31,980 (8.1) | 13,930 (8.8) |
| **Hypertension (%)** | 42,650 (29.2) | 28,330 (25.1) | 11,230 (28.9) | 2,355 (20.2) | 22,605 (28.1) | 28,000 (34.7) | 19,425 (39.7) | 7,195 (29.4) | 3,740 (22.2) | 3,125 (27.1) | 1,945 (17.9) | 3,895 (17.4) | 890 (20.6) | 11,775 (20.6) | 881,340 (24.4) | 8,805 (28.3) | 605 (22.8) | 81,450 (20.6) | 33,435 (21.1) |
| **Obesity (%)** | 14,720 (10.1) | 16,080 (14.3) | 3,700 (9.5) | 395 (3.4) | 7,395 (9.2) | 11,345 (14.0) | 7,005 (14.3) | 3,265 (13.3) | 1,890 (11.2) | 1,355 (11.8) | 890 (8.2) | 2,150 (9.6) | 540 (12.5) | 5,615 (9.8) | 381,860 (10.6) | 2,835 (9.1) | 280 (10.5) | 35,250 (8.9) | 16,065 (10.1) |
| **Osteoporosis (%)** | 3,345 (2.3) | 1,520 (1.3) | 520 (1.3) | 200 (1.7) | 1,180 (1.5) | 725 (0.9) | 485 (1.0) | 195 (0.8) | 120 (0.7) | 100 (0.9) | 110 (1.0) | 270 (1.2) | 65 (1.5) | 995 (1.7) | 100,920 (2.8) | 1,490 (4.8) | 35 (1.3) | 7,595 (1.9) | 3,280 (2.1) |
| **Rheumatoid Arthritis (%)** | 2,455 (1.7) | 1,675 (1.5) | 530 (1.4) | 105 (0.9) | 930 (1.2) | 560 (0.7) | 635 (1.3) | 245 (1.0) | 185 (1.1) | 120 (1.0) | 115 (1.1) | 185 (0.8) | 40 (0.9) | 540 (0.9) | 44,020 (1.2) | 465 (1.5) | 30 (1.1) | 3,710 (0.9) | 1,535 (1.0) |
| **Schizophrenia (%)** | 770 (0.5) | 885 (0.8) | 405 (1.0) | 65 (0.6) | 470 (0.6) | 850 (1.1) | 800 (1.6) | 410 (1.7) | 240 (1.4) | 115 (1.0) | 75 (0.7) | 195 (0.9) | 35 (0.8) | 330 (0.6) | 14,215 (0.4) | 200 (0.6) | 10 (0.4) | 1,435 (0.4) | 570 (0.4) |
| **Anti-hypertensive drugs (%)** | 355 (0.2) | 255 (0.2) | 140 (0.4) | 15 (0.1) | 140 (0.2) | 455 (0.6) | 380 (0.8) | 115 (0.5) | 50 (0.3) | 50 (0.4) | 10 (0.1) | 45 (0.2) | <10 (<0.2) | 105 (0.2) | 6,480 (0.2) | 65 (0.2) | <10 (<0.4) | 680 (0.2) | 240 (0.2) |
| **Antipsychotic (%)** | 255 (0.2) | 225 (0.2) | 80 (0.2) | 25 (0.2) | 160 (0.2) | 160 (0.2) | 195 (0.4) | 85 (0.3) | 60 (0.4) | 30 (0.3) | 25 (0.2) | 70 (0.3) | 15 (0.3) | 155 (0.3) | 12,580 (0.3) | 185 (0.6) | <10 (<0.4) | 920 (0.2) | 495 (0.3) |
| **Anti-coagulant drugs (%)** | 2,540 (1.7) | 1,975 (1.8) | 425 (1.1) | 155 (1.3) | 1,210 (1.5) | 1,255 (1.6) | 1,655 (3.4) | 515 (2.1) | 345 (2.0) | 195 (1.7) | 160 (1.5) | 375 (1.7) | 80 (1.8) | 1,270 (2.2) | 168,330 (4.7) | 2,085 (6.7) | 65 (2.4) | 11,790 (3.0) | 5,265 (3.3) |
| **Anti-diabetic drugs (%)** | 28,000 (19.1) | 24,985 (22.2) | 10,735 (27.7) | 965 (8.3) | 14,695 (18.3) | 10,865 (13.4) | 7,800 (16.0) | 2,590 (10.6) | 1,310 (7.8) | 1,130 (9.8) | 1,105 (10.2) | 1,765 (7.9) | 630 (14.5) | 5,615 (9.8) | 245,955 (6.8) | 2,235 (7.2) | 230 (8.7) | 23,400 (5.9) | 9,950 (6.3) |
| **Anti-platelet drugs (%)** | 13,860 (9.5) | 12,975 (11.5) | 4,260 (11.0) | 535 (4.6) | 6,115 (7.6) | 3,915 (4.8) | 4,165 (8.5) | 1,205 (4.9) | 780 (4.6) | 480 (4.2) | 590 (5.4) | 965 (4.3) | 380 (8.8) | 3,680 (6.4) | 276,890 (7.7) | 3,295 (10.6) | 150 (5.6) | 21,975 (5.5) | 9,390 (5.9) |
| **Statins (%)** | 39,815 (27.2) | 32,310 (28.7) | 13,900 (35.8) | 1,570 (13.5) | 20,045 (24.9) | 12,460 (15.4) | 10,515 (21.5) | 3,460 (14.1) | 1,915 (11.4) | 1,485 (12.9) | 1,635 (15.1) | 2,790 (12.5) | 955 (22.1) | 9,970 (17.4) | 692,875 (19.2) | 7,690 (24.7) | 460 (17.3) | 56,865 (14.4) | 24,225 (15.3) |
| **CVD history (ever) (%)** | 19,030 (13.0) | 17,355 (15.4) | 5,245 (13.5) | 930 (8.0) | 8,950 (11.1) | 7,000 (8.7) | 7,655 (15.7) | 2,405 (9.8) | 1,595 (9.5) | 945 (8.2) | 945 (8.7) | 1,865 (8.3) | 505 (11.7) | 6,395 (11.2) | 572,375 (15.8) | 6,680 (21.5) | 280 (10.5) | 44,385 (11.2) | 19,365 (12.2) |
| **CVD history (1-year prior) (%)** | 6,075 (4.2) | 5,740 (5.1) | 1,745 (4.5) | 305 (2.6) | 2,815 (3.5) | 2,190 (2.7) | 2,915 (6.0) | 785 (3.2) | 525 (3.1) | 275 (2.4) | 320 (3.0) | 585 (2.6) | 175 (4.0) | 2,170 (3.8) | 208,795 (5.8) | 2,745 (8.8) | 95 (3.6) | 15,265 (3.9) | 6,290 (4.0) |
| **28-days mortality (%)** | 2,645 (1.8) | 2,265 (2.0) | 880 (2.3) | 240 (2.1) | 1,085 (1.3) | 1,050 (1.3) | 1,675 (3.4) | 385 (1.6) | 345 (2.0) | 100 (0.9) | 120 (1.1) | 200 (0.9) | 40 (0.9) | 930 (1.6) | 78,640 (2.2) | 980 (3.2) | 20 (0.8) | 4,645 (1.2) | 2,370 (1.5) |
| **30-days CVD (%)** | 2,355 (1.6) | 2,495 (2.2) | 765 (2.0) | 150 (1.3) | 1,180 (1.5) | 845 (1.0) | 1,180 (2.4) | 295 (1.2) | 205 (1.2) | 90 (0.8) | 125 (1.2) | 205 (0.9) | 85 (2.0) | 855 (1.5) | 60,630 (1.7) | 815 (2.6) | 35 (1.3) | 4,815 (1.2) | 1,790 (1.1) |

Supplementary Table 7. Number of events and age-standardised IR of in 28-day mortality and 30-day CVD (100,000 population/year) in Wales by sex and in each ethnic group from 6 High-level ethnic groups and 10 ethnic codes. Estimates are reported with their 95% confidence intervals. Abbreviations: Age-std, age-standardised; CI, confidence interval; CVD, Cardiovascular disease.

| Outcome | Ethnic classification | Ethnic code | Men results | | | | Women results | | | |
| --- | --- | --- | --- | --- | --- | --- | --- | --- | --- | --- |
|  |  |  | Number of outcome events in men | Age-std IR | Low 95%CI | High 95%CI | Number of outcome events in women | Age-std IR | Low 95%CI | High 95%CI |
| 28-day mortality | High-level groups | Asian or Asian British | 110 | 70.76 | 57.41 | 84.10 | 85 | 49.58 | 37.18 | 64.88 |
| 28-day mortality | High-level groups | Black or Black British | 20 | 57.26 | 43.83 | 73.58 | <10 | 16.10 | 9.90 | 25.98 |
| 28-day mortality | High-level groups | Mixed | 15 | 86.91 | 70.04 | 106.66 | <10 | 16.59 | 9.90 | 25.98 |
| 28-day mortality | High-level groups | Other Ethnic Group | 10 | 31.93 | 22.24 | 44.51 | 10 | 23.57 | 15.43 | 34.60 |
| 28-day mortality | High-level groups | Unknown | 430 | 105.04 | 95.11 | 114.96 | 360 | 65.90 | 59.10 | 72.69 |
| 28-day mortality | High-level groups | White | 4275 | 46.33 | 44.95 | 47.72 | 3600 | 29.30 | 28.35 | 30.26 |
| 28-day mortality | 10 ethnic codes | Bangladeshi | 65 | 103.31 | 84.80 | 124.71 | 55 | 58.08 | 44.54 | 74.51 |
| 28-day mortality | 10 ethnic codes | Black African | <10 | 83.60 | 67.00 | 102.89 | <10 | 14.34 | 8.40 | 23.49 |
| 28-day mortality | 10 ethnic codes | Black Caribbean | <10 | 70.60 | 55.45 | 88.44 | <10 | 24.42 | 16.18 | 35.71 |
| 28-day mortality | 10 ethnic codes | Chinese | 0 | - | - | 3.69 | <10 | 67.49 | 52.81 | 85.09 |
| 28-day mortality | 10 ethnic codes | Indian | 15 | 40.18 | 29.14 | 54.10 | <10 | 244.44 | 215.28 | 276.61 |
| 28-day mortality | 10 ethnic codes | Mixed | 15 | 86.91 | 70.04 | 106.66 | <10 | 16.59 | 9.90 | 25.98 |
| 28-day mortality | 10 ethnic codes | Other Ethnic Group | 30 | 36.09 | 25.71 | 49.37 | 20 | 33.73 | 23.74 | 46.62 |
| 28-day mortality | 10 ethnic codes | Pakistani | 15 | 61.04 | 47.13 | 77.84 | <10 | 38.05 | 27.73 | 52.16 |
| 28-day mortality | 10 ethnic codes | Unknown | 430 | 105.04 | 95.11 | 114.96 | 360 | 65.90 | 59.10 | 72.69 |
| 28-day mortality | 10 ethnic codes | White | 4275 | 46.33 | 44.95 | 47.72 | 3600 | 29.30 | 28.35 | 30.26 |
| 30-day CVD | High-level groups | Asian or Asian British | 35 | 18.26 | 11.25 | 28.15 | 30 | 14.74 | 8.57 | 23.79 |
| 30-day CVD | High-level groups | Black or Black British | <10 | 13.16 | 7.65 | 22.23 | <10 | 11.12 | 6.20 | 19.68 |
| 30-day CVD | High-level groups | Mixed | <10 | 18.18 | 11.44 | 28.45 | <10 | 2.58 | 0.62 | 7.23 |
| 30-day CVD | High-level groups | Other Ethnic Group | 10 | 16.63 | 10.00 | 26.15 | <10 | 5.35 | 2.20 | 11.67 |
| 30-day CVD | High-level groups | Unknown | 70 | 15.13 | 8.86 | 24.29 | 40 | 7.80 | 3.64 | 14.82 |
| 30-day CVD | High-level groups | White | 1310 | 12.00 | 11.35 | 12.65 | 930 | 7.22 | 6.75 | 7.68 |
| 30-day CVD | 10 ethnic codes | Bangladeshi | 10 | 17.76 | 10.86 | 27.54 | 15 | 18.57 | 11.49 | 28.53 |
| 30-day CVD | 10 ethnic codes | Black African | <10 | 1.09 | 0.24 | 5.57 | <10 | 17.02 | 10.67 | 27.22 |
| 30-day CVD | 10 ethnic codes | Black Caribbean | <10 | 25.04 | 16.98 | 36.91 | <10 | 8.17 | 4.12 | 15.76 |
| 30-day CVD | 10 ethnic codes | Chinese | <10 | 37.52 | 26.89 | 51.00 | <10 | 15.97 | 9.15 | 24.74 |
| 30-day CVD | 10 ethnic codes | Indian | <10 | 5.71 | 2.20 | 11.67 | <10 | 8.06 | 4.12 | 15.76 |
| 30-day CVD | 10 ethnic codes | Mixed | <10 | 18.18 | 11.44 | 28.45 | <10 | 2.58 | 0.62 | 7.23 |
| 30-day CVD | 10 ethnic codes | Other Ethnic Group | 20 | 16.85 | 10.17 | 26.42 | <10 | 7.65 | 3.45 | 14.42 |
| 30-day CVD | 10 ethnic codes | Pakistani | 10 | 30.38 | 20.96 | 42.69 | <10 | 24.69 | 16.18 | 35.71 |
| 30-day CVD | 10 ethnic codes | Unknown | 70 | 15.13 | 8.86 | 24.29 | 40 | 7.80 | 3.64 | 14.82 |
| 30-day CVD | 10 ethnic codes | White | 1310 | 12.00 | 11.35 | 12.65 | 930 | 7.22 | 6.75 | 7.68 |

Supplementary Table 8. Number of events and age-standardised IR of in 28-day mortality and 30-day CVD (100,000 population/year) in England by sex and in each ethnic group from 6 High-level ethnic groups, 19 NHS ethnicity codes and SNOMED-CT concepts. Estimates are reported with their 95% confidence intervals. Abbreviations: Age-std, age-standardised; CI, confidence interval; CVD, Cardiovascular disease; Middle eastern*, excluding Israeli, Iranian and Arab.

| **Outcome** | **Ethnic classification** | **Ethnic code** | **Men results** | | | | **Women results** | | | |
| --- | --- | --- | --- | --- | --- | --- | --- | --- | --- | --- |
|  |  |  | **Number of outcome events in men** | **Age-std IR** | **Low 95%CI** | **High 95%CI** | **Number of outcome events in women** | **Age-std IR** | **Low 95%CI** | **High 95%CI** |
| 28-day mortality | High-level groups | Asian or Asian British | 4390 | 74.20 | 72.00 | 76.39 | 2730 | 44.50 | 42.80 | 46.10 |
| 28-day mortality | High-level groups | Black or Black British | 1890 | 82.79 | 79.06 | 86.52 | 1220 | 43.50 | 41.10 | 46.00 |
| 28-day mortality | High-level categories | Mixed | 445 | 75.22 | 68.25 | 82.19 | 325 | 46.60 | 41.50 | 51.70 |
| 28-day mortality | High-level groups | Other Ethnic Group | 580 | 71.05 | 65.26 | 76.84 | 395 | 42.70 | 38.50 | 46.90 |
| 28-day mortality | High-level groups | Unknown | 1310 | 45.37 | 42.91 | 47.82 | 1060 | 25.50 | 24.00 | 27.00 |
| 28-day mortality | High-level groups | White | 46290 | 48.88 | 48.44 | 49.33 | 37995 | 28.80 | 28.50 | 29.10 |
| 28-day mortality | NHS ethnicity codes | African | 650 | 83.62 | 77.19 | 90.05 | 400 | 45.60 | 41.10 | 50.10 |
| 28-day mortality | NHS ethnicity codes | Any other Asian background | 695 | 66.48 | 61.53 | 71.43 | 390 | 38.40 | 34.60 | 42.20 |
| 28-day mortality | NHS ethnicity codes | Any other Black background | 245 | 90.68 | 79.27 | 102.08 | 145 | 46.80 | 39.10 | 54.50 |
| 28-day mortality | NHS ethnicity codes | Any Other Ethnic Group | 560 | 73.88 | 67.75 | 80.01 | 375 | 42.60 | 38.20 | 46.90 |
| 28-day mortality | NHS ethnicity codes | Any other Mixed background | 115 | 59.96 | 48.91 | 71.02 | 90 | 35.60 | 25.30 | 48.80 |
| 28-day mortality | NHS ethnicity codes | Any other White background | 2595 | 44.53 | 42.81 | 46.24 | 2045 | 24.30 | 23.30 | 25.40 |
| 28-day mortality | NHS ethnicity codes | Arab | 20 | 45.31 | 33.51 | 60.00 | 20 | 49.20 | 36.80 | 64.40 |
| 28-day mortality | NHS ethnicity codes | Bangladeshi | 535 | 116.77 | 106.89 | 126.65 | 345 | 65.50 | 58.50 | 72.40 |
| 28-day mortality | NHS ethnicity codes | British | 43135 | 49.17 | 48.70 | 49.63 | 35505 | 29.20 | 28.90 | 29.50 |
| 28-day mortality | NHS ethnicity codes | Caribbean | 995 | 81.69 | 76.61 | 86.76 | 680 | 41.70 | 38.50 | 44.80 |
| 28-day mortality | NHS ethnicity codes | Chinese | 150 | 94.01 | 78.87 | 109.16 | 95 | 50.30 | 37.80 | 65.70 |
| 28-day mortality | NHS ethnicity codes | Gypsy or Irish Traveller | 10 | 27.20 | 18.36 | 38.93 | <10 | 34.90 | 24.70 | 47.90 |
| 28-day mortality | NHS ethnicity codes | Indian | 1620 | 64.60 | 61.45 | 67.75 | 1030 | 39.50 | 37.10 | 42.00 |
| 28-day mortality | NHS ethnicity codes | Irish | 545 | 47.86 | 43.84 | 51.88 | 435 | 27.20 | 24.70 | 29.80 |
| 28-day mortality | NHS ethnicity codes | Pakistani | 1395 | 81.31 | 77.04 | 85.58 | 870 | 49.70 | 46.40 | 52.90 |
| 28-day mortality | NHS ethnicity codes | Unknown/Not stated | 1310 | 45.37 | 42.91 | 47.82 | 1060 | 25.50 | 24.00 | 27.00 |
| 28-day mortality | NHS ethnicity codes | White and Asian | 75 | 72.66 | 57.36 | 90.85 | 45 | 43.90 | 32.30 | 58.40 |
| 28-day mortality | NHS ethnicity codes | White and Black African | 60 | 64.50 | 50.17 | 81.73 | 40 | 40.90 | 29.80 | 54.90 |
| 28-day mortality | NHS ethnicity codes | White and Black Caribbean | 195 | 96.28 | 82.84 | 109.73 | 150 | 64.80 | 54.50 | 75.20 |
| 28-day mortality | SNOMED-CT concept | Central/South/Latin American | <10 | 178.47 | 153.74 | 206.16 | 10 | 55.20 | 42.10 | 71.30 |
| 28-day mortality | SNOMED-CT concept | Filipino | 15 | 57.34 | 43.90 | 73.67 | 15 | 40.30 | 29.20 | 54.20 |
| 28-day mortality | SNOMED-CT concept | Iranian | 10 | 33.35 | 23.42 | 46.17 | <10 | 10.20 | 5.50 | 18.40 |
| 28-day mortality | SNOMED-CT concept | Jewish | 15 | 37.00 | 26.47 | 50.42 | 10 | 25.70 | 17.10 | 37.10 |
| 28-day mortality | SNOMED-CT concept | Middle Eastern* | <10 | 35.98 | 25.21 | 48.68 | <10 | 29.40 | 20.20 | 41.60 |
| 28-day mortality | SNOMED-CT concept | Nigerian | <10 | 137.01 | 115.94 | 161.96 | <10 | 37.70 | 26.90 | 51.00 |
| 28-day mortality | SNOMED-CT concept | Punjabi | <10 | 20.68 | 13.00 | 30.89 | 10 | 29.20 | 20.00 | 41.30 |
| 28-day mortality | SNOMED-CT concept | Somali | 25 | 89.95 | 72.77 | 110.02 | 15 | 34.10 | 24.10 | 47.10 |
| 28-day mortality | SNOMED-CT concept | Turkish or Turkish Cypriot | 60 | 77.57 | 61.72 | 96.32 | 30 | 36.30 | 25.80 | 49.60 |
| 30-day CVD | High-level groups | Asian or Asian British | 4665 | 64.45 | 62.60 | 66.30 | 2280 | 31.20 | 29.90 | 32.50 |
| 30-day CVD | High-level groups | Mixed | 1340 | 51.17 | 48.42 | 53.91 | 260 | 30.20 | 26.50 | 33.90 |
| 30-day CVD | High-level groups | Black or Black British | 365 | 48.37 | 43.40 | 53.33 | 980 | 29.60 | 27.70 | 31.40 |
| 30-day CVD | High-level groups | Other Ethnic Group | 625 | 60.71 | 55.95 | 65.46 | 315 | 29.20 | 26.00 | 32.50 |
| 30-day CVD | High-level groups | White | 1070 | 32.62 | 30.66 | 34.57 | 25920 | 20.10 | 19.90 | 20.40 |
| 30-day CVD | High-level groups | Unknown | 40370 | 39.85 | 39.46 | 40.24 | 720 | 17.40 | 16.10 | 18.70 |
| 30-day CVD | NHS ethnicity codes | African | 495 | 48.44 | 44.18 | 52.70 | 345 | 25.50 | 22.80 | 28.20 |
| 30-day CVD | NHS ethnicity codes | Any other Asian background | 800 | 57.10 | 53.14 | 61.06 | 385 | 27.90 | 25.10 | 30.70 |
| 30-day CVD | NHS ethnicity codes | Any other Black background | 185 | 48.63 | 41.60 | 55.65 | 110 | 30.90 | 25.10 | 36.70 |
| 30-day CVD | NHS ethnicity codes | Any Other Ethnic Group | 565 | 59.74 | 54.82 | 64.67 | 290 | 28.30 | 25.00 | 31.50 |
| 30-day CVD | NHS ethnicity codes | Any other Mixed background | 125 | 51.42 | 42.41 | 60.44 | 80 | 26.10 | 17.50 | 37.70 |
| 30-day CVD | NHS ethnicity codes | Any other White background | 2960 | 43.92 | 42.34 | 45.50 | 1855 | 21.60 | 20.60 | 22.60 |
| 30-day CVD | NHS ethnicity codes | Arab | 60 | 71.82 | 56.62 | 89.91 | 25 | 50.90 | 38.30 | 66.40 |
| 30-day CVD | NHS ethnicity codes | Bangladeshi | 520 | 88.74 | 81.12 | 96.37 | 245 | 38.30 | 33.50 | 43.10 |
| 30-day CVD | NHS ethnicity codes | British | 36890 | 39.47 | 39.07 | 39.88 | 23740 | 20.00 | 19.70 | 20.20 |
| 30-day CVD | NHS ethnicity codes | Caribbean | 655 | 56.39 | 52.08 | 60.71 | 520 | 32.10 | 29.30 | 34.90 |
| 30-day CVD | NHS ethnicity codes | Chinese | 100 | 50.09 | 37.61 | 65.45 | 50 | 20.70 | 13.20 | 31.10 |
| 30-day CVD | NHS ethnicity codes | Gypsy or Irish Traveller | 15 | 37.01 | 26.47 | 50.43 | 15 | 38.60 | 27.80 | 52.30 |
| 30-day CVD | NHS ethnicity codes | Indian | 1555 | 52.02 | 49.43 | 54.60 | 800 | 27.20 | 25.30 | 29.10 |
| 30-day CVD | NHS ethnicity codes | Irish | 505 | 45.39 | 41.43 | 49.35 | 310 | 21.70 | 19.20 | 24.10 |
| 30-day CVD | NHS ethnicity codes | Pakistani | 1690 | 85.03 | 80.98 | 89.08 | 800 | 39.20 | 36.50 | 41.90 |
| 30-day CVD | NHS ethnicity codes | Unknown/Not stated | 1070 | 32.62 | 30.66 | 34.57 | 720 | 17.40 | 16.10 | 18.70 |
| 30-day CVD | NHS ethnicity codes | White and Asian | 80 | 55.55 | 42.34 | 71.65 | 40 | 24.70 | 16.30 | 35.90 |
| 30-day CVD | NHS ethnicity codes | White and Black African | 50 | 51.98 | 39.25 | 67.60 | 40 | 26.90 | 18.10 | 38.60 |
| 30-day CVD | NHS ethnicity codes | White and Black Caribbean | 105 | 47.95 | 38.86 | 57.03 | 100 | 38.70 | 31.10 | 46.30 |
| 30-day CVD | SNOMED-CT concept | Central/South/Latin American | 10 | 62.26 | 48.20 | 79.21 | <10 | 19.60 | 12.20 | 29.70 |
| 30-day CVD | SNOMED-CT concept | Filipino | 20 | 66.69 | 52.09 | 84.19 | 10 | 23.00 | 15.00 | 33.90 |
| 30-day CVD | SNOMED-CT concept | Iranian | 20 | 41.76 | 30.49 | 55.92 | <10 | 27.20 | 18.60 | 39.30 |
| 30-day CVD | SNOMED-CT concept | Jewish | 20 | 48.20 | 35.99 | 63.30 | 15 | 35.50 | 25.20 | 48.70 |
| 30-day CVD | SNOMED-CT concept | Middle Eastern* | 10 | 34.81 | 24.63 | 47.87 | <10 | 73.50 | 58.10 | 91.80 |
| 30-day CVD | SNOMED-CT concept | Nigerian | <10 | 29.17 | 20.24 | 41.65 | <10 | 32.10 | 22.70 | 45.20 |
| 30-day CVD | SNOMED-CT concept | Punjabi | 15 | 39.18 | 28.30 | 52.95 | 10 | 30.10 | 20.70 | 42.30 |
| 30-day CVD | SNOMED-CT concept | Somali | 25 | 62.15 | 48.10 | 79.09 | 15 | 21.50 | 13.80 | 32.10 |
| 30-day CVD | SNOMED-CT concept | Turkish or Turkish Cypriot | 105 | 93.16 | 75.26 | 111.07 | 50 | 44.20 | 32.50 | 58.70 |

### Supplementary Table 9. Adjusted hazard ratios of 28-day mortality from individuals diagnosed with COVID-19 with diverse ethnic background in England, using White British women as reference group. Ethnic classification used: the 19 NHS ethnicity codes.

|  | **Men** | | | **Women** | | |
| --- | --- | --- | --- | --- | --- | --- |
| **Variables of adjustment** | **HR** | **Low 95%CI** | **High 95%CI** | **HR** | **Low 95%CI** | **High 95%CI** |
| Indian | 1.15 | 1.10 | 1.21 | 1.20 | 1.12 | 1.27 |
| Pakistani | 1.31 | 1.24 | 1.38 | 1.34 | 1.25 | 1.44 |
| Bangladeshi | 1.62 | 1.48 | 1.76 | 1.65 | 1.48 | 1.84 |
| Chinese | 1.81 | 1.54 | 2.13 | 1.75 | 1.43 | 2.15 |
| Any other Asian background | 1.15 | 1.07 | 1.25 | 1.04 | 0.94 | 1.15 |
| African | 1.32 | 1.22 | 1.43 | 1.21 | 1.09 | 1.34 |
| Caribbean | 1.00 | 0.94 | 1.07 | 1.04 | 0.96 | 1.12 |
| Any other Black background | 1.45 | 1.28 | 1.64 | 1.32 | 1.12 | 1.56 |
| White and Black Caribbean | 1.37 | 1.19 | 1.57 | 1.48 | 1.26 | 1.74 |
| White and Black African | 1.14 | 0.89 | 1.47 | 1.07 | 0.79 | 1.45 |
| White and Asian | 1.46 | 1.17 | 1.83 | 1.27 | 0.95 | 1.70 |
| Any other Mixed background | 1.14 | 0.95 | 1.37 | 1.08 | 0.88 | 1.33 |
| Arab | 0.56 | 0.36 | 0.86 | 1.24 | 0.80 | 1.93 |
| Any Other Ethnic Group | 1.23 | 1.13 | 1.34 | 1.16 | 1.04 | 1.28 |
| Irish | 0.87 | 0.80 | 0.95 | 0.92 | 0.83 | 1.01 |
| Gypsy or Irish Traveller | 0.67 | 0.38 | 1.18 | 0.89 | 0.46 | 1.71 |
| Any other White background | 0.91 | 0.87 | 0.94 | 0.84 | 0.80 | 0.88 |
| Unknown/Not stated | 0.93 | 0.88 | 0.99 | 0.89 | 0.84 | 0.94 |
| Age | 1.28 | 1.27 | 1.29 | 1.33 | 1.32 | 1.34 |
| I(age^2) | 1.00 | 1.00 | 1.00 | 1.00 | 1.00 | 1.00 |
| Pregnancy (yes) | *Not included in the adjustment* | | | 0.75 | 0.24 | 2.33 |
| Vaccination (yes) | 0.49 | 0.47 | 0.51 | 0.50 | 0.48 | 0.52 |
| IMD quintile: IMD 2 | 0.90 | 0.88 | 0.92 | 0.92 | 0.89 | 0.95 |
| IMD quintile: IMD 3 | 0.83 | 0.81 | 0.85 | 0.86 | 0.83 | 0.88 |
| IMD quintile: IMD 4 | 0.78 | 0.76 | 0.80 | 0.81 | 0.79 | 0.84 |
| IMD quintile: IMD 5 (less deprived) | 0.74 | 0.72 | 0.76 | 0.79 | 0.76 | 0.81 |
| IMD quintile: IMD Unknown | 0.85 | 0.60 | 1.20 | 0.79 | 0.53 | 1.18 |
| English region: East of England | 1.04 | 1.00 | 1.08 | 1.07 | 1.02 | 1.12 |
| English region: London | 0.91 | 0.87 | 0.94 | 0.96 | 0.92 | 1.01 |
| English region: North East | 0.91 | 0.86 | 0.95 | 0.98 | 0.93 | 1.04 |
| English region: North West | 0.95 | 0.91 | 0.98 | 1.00 | 0.96 | 1.04 |
| English region: South East | 0.98 | 0.94 | 1.02 | 1.02 | 0.97 | 1.06 |
| English region: South West | 0.87 | 0.83 | 0.91 | 0.88 | 0.83 | 0.93 |
| English region: West Midlands | 0.92 | 0.88 | 0.95 | 0.94 | 0.90 | 0.98 |
| English region: Yorkshire and The Humber | 0.98 | 0.94 | 1.02 | 1.02 | 0.98 | 1.07 |
| English region: Unknown | 0.93 | 0.89 | 0.97 | 1.00 | 0.96 | 1.05 |
| Recruiting Months:  01 Jul 2020 to 31 Dec 2020 | 1.11 | 0.78 | 1.59 | 0.89 | 0.56 | 1.39 |
| Recruiting Months:  01 Jan 2021 to 30 Jun 2021 | 1.40 | 0.98 | 2.01 | 1.09 | 0.69 | 1.72 |
| Recruiting Months:  01 Jul 2021 to 31 Dec 2021 | 1.43 | 0.92 | 2.21 | 1.20 | 0.71 | 2.05 |
| Recruiting Months:  01 Jan 2022 to 01 Apr 2022 | 1.89 | 1.20 | 2.96 | 1.47 | 0.85 | 2.54 |
| Atrial Fibrillation (yes) | 1.10 | 1.07 | 1.12 | 1.15 | 1.12 | 1.18 |
| Obesity (yes) | 1.07 | 1.04 | 1.10 | 1.13 | 1.09 | 1.16 |
| Chronic Kidney Disease (yes) | 1.40 | 1.38 | 1.43 | 1.36 | 1.33 | 1.38 |
| Diabetes (yes) | 1.31 | 1.28 | 1.33 | 1.37 | 1.34 | 1.40 |
| Chronic mental health disorders (yes) | 1.25 | 1.19 | 1.30 | 1.23 | 1.18 | 1.28 |
| Use of CVD prevention medication (yes) | 1.09 | 1.06 | 1.11 | 1.10 | 1.07 | 1.12 |
| Rheumatoid Arthritis (yes) | 1.36 | 1.29 | 1.44 | 1.44 | 1.37 | 1.50 |
| Antipsychotic (yes) | 1.30 | 1.22 | 1.38 | 1.25 | 1.17 | 1.34 |
| Cancer (yes) | 1.28 | 1.25 | 1.31 | 1.10 | 1.07 | 1.12 |
| Chronic obstructive pulmonary disease (yes) | 1.44 | 1.41 | 1.48 | 1.67 | 1.62 | 1.71 |
| Dementia (yes) | 1.44 | 1.41 | 1.48 | 1.29 | 1.26 | 1.32 |
| Hypertension (yes) | 0.97 | 0.95 | 0.99 | 1.03 | 1.01 | 1.05 |
| **Reference groups**: White British for ethnicity; IMD 1 (most deprived) for IMD quintile; East Midlands for English region; 23 Jan 2020 to 30 Jun 2020 for Recruiting Months. **Abbreviations:** CI, confidence interval; CVD, cardiovascular disease; IMD, index of multiple deprivation; I(age^2), main effect and the second order interaction of age. | | | | | | |

### Supplementary Table 10. Adjusted hazard ratios of 30-day CVD from individuals diagnosed with COVID-19 with diverse ethnic background in England, using White British women as reference group. Ethnic classification used: the 19 NHS ethnicity codes.

|  | **Men** | | | **Women** | | |
| --- | --- | --- | --- | --- | --- | --- |
| **Variables of adjustment** | **HR** | **Low 95%CI** | **High 95%CI** | **HR** | **Low 95%CI** | **High 95%CI** |
| Indian | 1.01 | 0.96 | 1.06 | 1.02 | 0.95 | 1.1 |
| Pakistani | 1.39 | 1.32 | 1.46 | 1.18 | 1.09 | 1.26 |
| Bangladeshi | 1.27 | 1.16 | 1.39 | 1.09 | 0.96 | 1.24 |
| Chinese | 1.41 | 1.16 | 1.72 | 1.25 | 0.94 | 1.65 |
| Any other Asian background | 1.18 | 1.10 | 1.27 | 1.06 | 0.96 | 1.17 |
| African | 0.95 | 0.87 | 1.05 | 1.14 | 1.02 | 1.27 |
| Caribbean | 1.07 | 0.99 | 1.16 | 1.2 | 1.1 | 1.32 |
| Any other Black background | 1.16 | 1.00 | 1.34 | 1.16 | 0.96 | 1.41 |
| White and Black Caribbean | 1.11 | 0.92 | 1.35 | 1.45 | 1.19 | 1.76 |
| White and Black African | 0.83 | 0.63 | 1.09 | 1.02 | 0.74 | 1.4 |
| White and Asian | 1.37 | 1.11 | 1.71 | 1.27 | 0.94 | 1.73 |
| Any other Mixed background | 1.14 | 0.95 | 1.35 | 1.14 | 0.91 | 1.41 |
| Arab | 1.48 | 1.15 | 1.91 | 1.77 | 1.2 | 2.6 |
| Any Other Ethnic Group | 1.31 | 1.21 | 1.43 | 1.21 | 1.08 | 1.36 |
| Irish | 1.05 | 0.96 | 1.15 | 1.05 | 0.93 | 1.17 |
| Gypsy or Irish Traveller | 0.91 | 0.57 | 1.47 | 1.71 | 1.05 | 2.79 |
| Any other White background | 1.09 | 1.05 | 1.13 | 1.02 | 0.98 | 1.07 |
| Unknown/Not stated | 0.87 | 0.81 | 0.92 | 0.93 | 0.87 | 1.01 |
| Age | 1.17 | 1.16 | 1.18 | 1.17 | 1.17 | 1.18 |
| I(age^2) | 1.00 | 1.00 | 1.00 | 1 | 1 | 1 |
| Pregnancy (yes) | *Not included in the adjustment* | | | 3.2 | 2.44 | 4.21 |
| Vaccination (yes) | 0.65 | 0.62 | 0.67 | 0.67 | 0.64 | 0.7 |
| IMD quintile: IMD 2 | 0.91 | 0.89 | 0.94 | 0.92 | 0.89 | 0.95 |
| IMD quintile: IMD 3 | 0.84 | 0.82 | 0.87 | 0.86 | 0.84 | 0.9 |
| IMD quintile: IMD 4 | 0.80 | 0.78 | 0.82 | 0.82 | 0.8 | 0.86 |
| IMD quintile: IMD 5 (less deprived) | 0.76 | 0.74 | 0.78 | 0.78 | 0.75 | 0.81 |
| IMD quintile: IMD Unknown | 1.15 | 0.80 | 1.67 | 1.58 | 1.07 | 2.33 |
| English region: East of England | 0.96 | 0.91 | 1.00 | 0.94 | 0.89 | 1 |
| English region: London | 1.02 | 0.98 | 1.06 | 1.03 | 0.98 | 1.09 |
| English region: North East | 0.89 | 0.84 | 0.94 | 0.93 | 0.87 | 0.99 |
| English region: North West | 1.07 | 1.03 | 1.11 | 1.17 | 1.11 | 1.23 |
| English region: South East | 1.01 | 0.97 | 1.05 | 1.04 | 0.99 | 1.1 |
| English region: South West | 0.98 | 0.94 | 1.04 | 0.95 | 0.89 | 1.01 |
| English region: West Midlands | 1.07 | 1.03 | 1.12 | 1.07 | 1.01 | 1.12 |
| English region: Yorkshire and The Humber | 0.98 | 0.94 | 1.02 | 1.04 | 0.98 | 1.09 |
| English region: Unknown | 0.96 | 0.92 | 1.01 | 0.99 | 0.93 | 1.04 |
| Recruiting Months:  01 Jul 2020 to 31 Dec 2020 | 2.11 | 1.57 | 2.84 | 2.18 | 1.49 | 3.17 |
| Recruiting Months:  01 Jan 2021 to 30 Jun 2021 | 5.18 | 3.84 | 7.00 | 4.62 | 3.15 | 6.78 |
| Recruiting Months:  01 Jul 2021 to 31 Dec 2021 | 11.05 | 7.75 | 15.74 | 11.81 | 7.47 | 18.66 |
| Recruiting Months:  01 Jan 2022 to 01 Apr 2022 | 16.15 | 11.28 | 23.13 | 16.49 | 10.37 | 26.21 |
| Atrial Fibrillation (yes) | 1.51 | 1.48 | 1.55 | 1.72 | 1.67 | 1.77 |
| Obesity (yes) | 1.11 | 1.08 | 1.14 | 1.14 | 1.1 | 1.18 |
| Chronic Kidney Disease (yes) | 1.59 | 1.56 | 1.62 | 1.52 | 1.48 | 1.56 |
| Diabetes (yes) | 1.25 | 1.22 | 1.27 | 1.3 | 1.27 | 1.34 |
| Chronic mental health disorders (yes) | 1.16 | 1.11 | 1.21 | 1.11 | 1.06 | 1.17 |
| Use of CVD prevention medication (yes) | 3.15 | 3.07 | 3.24 | 3.25 | 3.14 | 3.36 |
| Rheumatoid Arthritis (yes) | 1.26 | 1.19 | 1.35 | 1.33 | 1.26 | 1.41 |
| Antipsychotic (yes) | 0.95 | 0.86 | 1.06 | 1 | 0.89 | 1.13 |
| Cancer (yes) | 1.15 | 1.12 | 1.18 | 0.91 | 0.89 | 0.94 |
| Chronic obstructive pulmonary disease (yes) | 1.60 | 1.56 | 1.64 | 1.83 | 1.77 | 1.89 |
| Dementia (yes) | 0.84 | 0.81 | 0.86 | 0.7 | 0.67 | 0.72 |
| Hypertension (yes) | 1.02 | 1.00 | 1.04 | 1.2 | 1.17 | 1.23 |
| **Reference groups**: White British for ethnicity; IMD 1 (most deprived) for IMD quintile; East Midlands for English region; 23 Jan 2020 to 30 Jun 2020 for Recruiting Months. **Abbreviations:** CI, confidence interval; CVD, cardiovascular disease; IMD, index of multiple deprivation; I(age^2), main effect and the second order interaction of age. | | | | | | |

### Supplementary Table 11. Adjusted hazard ratios of 28-day mortality from individuals diagnosed with COVID-19 with diverse ethnic background in Wales, using White women as reference group. Ethnic classification used: the 6 High-level groups.

|  | **Men** | | | **Women** | | |
| --- | --- | --- | --- | --- | --- | --- |
| **Variables of adjustment** | **HR** | **Low 95%CI** | **High 95%CI** | **HR** | **Low 95%CI** | **High 95%CI** |
| Age | 1.32 | 1.28 | 1.35 | 1.31 | 1.27 | 1.34 |
| I(age^2) | 1.00 | 1.00 | 1.00 | 1.00 | 1.00 | 1.00 |
| Asian or Asian British | 1.57 | 1.30 | 1.90 | 1.79 | 1.44 | 2.23 |
| Black or Black British | 1.25 | 0.79 | 2.00 | 0.68 | 0.33 | 1.44 |
| Mixed | 1.53 | 0.94 | 2.51 | 0.96 | 0.50 | 1.85 |
| Other Ethnic Group | 0.76 | 0.42 | 1.37 | 1.34 | 0.72 | 2.49 |
| Unknown | 2.38 | 2.15 | 2.63 | 2.20 | 1.97 | 2.45 |
| Vaccination (yes) | 0.44 | 0.37 | 0.51 | 0.45 | 0.38 | 0.52 |
| IMD quintile: IMD 2 | 0.95 | 0.87 | 1.04 | 0.96 | 0.88 | 1.06 |
| IMD quintile: IMD 3 | 0.96 | 0.88 | 1.05 | 0.90 | 0.81 | 0.99 |
| IMD quintile: IMD 4 | 0.87 | 0.80 | 0.96 | 0.89 | 0.80 | 0.98 |
| IMD quintile: IMD 5 (less deprived) | 0.85 | 0.78 | 0.94 | 0.79 | 0.72 | 0.88 |
| IMD quintile: IMD Unknown | 0.90 | 0.79 | 1.03 | 0.88 | 0.77 | 1.00 |
| Recruiting Months: 01 Jul 2020 to 31 Dec 2020 | 0.44 | 0.06 | 3.10 | 2.70 | 0.26 | 27.54 |
| Recruiting Months: 01 Jan 2021 to 30 Jun 2021 | 0.86 | 0.12 | 6.05 | 5.25 | 0.51 | 54.00 |
| Recruiting Months: 01 Jul 2021 to 31 Dec 2021 | 1.44 | 0.14 | 14.66 | 4.28 | 0.30 | 61.39 |
| Recruiting Months: 01 Jan 2022 to 01 Apr 2022 | 2.14 | 0.21 | 22.28 | 6.76 | 0.46 | 99.57 |
| Atrial Fibrillation (yes) | 1.35 | 1.26 | 1.44 | 1.32 | 1.23 | 1.42 |
| Obesity (yes) | 1.14 | 1.07 | 1.21 | 1.13 | 1.05 | 1.21 |
| Chronic Kidney Disease (yes) | 1.27 | 1.19 | 1.36 | 1.25 | 1.17 | 1.35 |
| Diabetes (yes) | 1.43 | 1.34 | 1.53 | 1.52 | 1.41 | 1.63 |
| Chronic mental health disorders (yes) | 1.11 | 1.04 | 1.19 | 1.11 | 1.04 | 1.18 |
| Use of CVD prevention medication (yes) | 1.02 | 0.95 | 1.10 | 1.00 | 0.93 | 1.08 |
| Rheumatoid Arthritis (yes) | 1.39 | 1.19 | 1.62 | 1.52 | 1.35 | 1.71 |
| Antipsychotic (yes) | 1.15 | 1.03 | 1.28 | 1.20 | 1.08 | 1.34 |
| Cancer (yes) | 1.35 | 1.27 | 1.43 | 1.25 | 1.17 | 1.33 |
| Chronic obstructive pulmonary disease (yes) | 1.57 | 1.47 | 1.68 | 1.71 | 1.59 | 1.84 |
| Dementia (yes) | 1.45 | 1.34 | 1.56 | 1.19 | 1.10 | 1.28 |
| Hypertension (yes) | 1.19 | 1.10 | 1.28 | 1.28 | 1.18 | 1.40 |
| **Reference groups**: White for ethnicity; IMD 1 (most deprived) for IMD quintile; 23 Jan 2020 to 30 Jun 2020 for Recruiting Months.  **Abbreviations:** CI, confidence interval; CVD, cardiovascular disease; IMD, index of multiple deprivation; I(age^2), main effect and the second order interaction of age. | | | | | | |

### Supplementary Table 12. Adjusted hazard ratios of 30-day CVD from individuals diagnosed with COVID-19 with diverse ethnic background in Wales, using White women as reference group. Ethnic classification used: the 6 High-level groups.

|  | **Men** | | | **Women** | | |
| --- | --- | --- | --- | --- | --- | --- |
| **Variables of adjustment** | **HR** | **Low 95%CI** | **High 95%CI** | **HR** | **Low 95%CI** | **High 95%CI** |
| Age | 1.14 | 1.11 | 1.18 | 1.11 | 1.07 | 1.15 |
| I(age^2) | 1.00 | 1.00 | 1.00 | 1.00 | 1.00 | 1.00 |
| Asian or Asian British | 1.20 | 0.85 | 1.68 | 1.83 | 1.28 | 2.62 |
| Black or Black British | 1.25 | 0.59 | 2.63 | 1.86 | 0.88 | 3.94 |
| Mixed | 1.69 | 0.84 | 3.39 | 1.15 | 0.43 | 3.07 |
| Other Ethnic Group | 1.45 | 0.80 | 2.63 | 0.49 | 0.12 | 1.97 |
| Unknown | 1.26 | 0.98 | 1.61 | 1.18 | 0.86 | 1.63 |
| Vaccination (yes) | 0.53 | 0.41 | 0.67 | 0.56 | 0.43 | 0.74 |
| IMD quintile: IMD 2 | 1.03 | 0.87 | 1.20 | 0.85 | 0.70 | 1.02 |
| IMD quintile: IMD 3 | 0.99 | 0.84 | 1.17 | 0.79 | 0.65 | 0.95 |
| IMD quintile: IMD 4 | 0.83 | 0.69 | 0.98 | 0.72 | 0.58 | 0.88 |
| IMD quintile: IMD 5 (less deprived) | 0.94 | 0.79 | 1.11 | 0.79 | 0.65 | 0.96 |
| IMD quintile: IMD Unknown | 0.96 | 0.74 | 1.25 | 0.89 | 0.67 | 1.19 |
| Recruiting Months: 01 Jul 2020 to 31 Dec 2020 | 2.53 | 0.26 | 24.87 | 3.35 | 0.43 | 25.97 |
| Recruiting Months: 01 Jan 2021 to 30 Jun 2021 | 3.27 | 0.32 | 33.23 | 5.58 | 0.69 | 45.21 |
| Recruiting Months: 01 Jul 2021 to 31 Dec 2021 | 839,265 | - | Inf | 9.69 | 0.52 | 180.98 |
| Recruiting Months: 01 Jan 2022 to 01 Apr 2022 | 1,676,978 | - | Inf | 15.41 | 0.79 | 299.86 |
| Atrial Fibrillation (yes) | 1.77 | 1.55 | 2.01 | 2.23 | 1.91 | 2.61 |
| Obesity (yes) | 1.14 | 1.01 | 1.28 | 1.21 | 1.06 | 1.39 |
| Chronic Kidney Disease (yes) | 1.08 | 0.92 | 1.27 | 1.14 | 0.96 | 1.35 |
| Diabetes (yes) | 1.00 | 0.88 | 1.14 | 1.08 | 0.92 | 1.27 |
| Chronic mental health disorders (yes) | 1.07 | 0.94 | 1.21 | 1.13 | 0.99 | 1.29 |
| Use of CVD prevention medication (yes) | 3.21 | 2.75 | 3.75 | 3.42 | 2.85 | 4.11 |
| Rheumatoid Arthritis (yes) | 1.25 | 0.88 | 1.77 | 0.86 | 0.62 | 1.19 |
| Antipsychotic (yes) | 0.59 | 0.40 | 0.88 | 0.85 | 0.60 | 1.18 |
| Cancer (yes) | 1.05 | 0.92 | 1.18 | 1.37 | 1.20 | 1.58 |
| Chronic obstructive pulmonary disease (yes) | 1.21 | 1.04 | 1.40 | 1.35 | 1.14 | 1.59 |
| Dementia (yes) | 0.51 | 0.39 | 0.66 | 0.50 | 0.39 | 0.64 |
| Hypertension (yes) | 0.99 | 0.87 | 1.13 | 1.04 | 0.88 | 1.22 |
| **Reference groups**: White for ethnicity; IMD 1 (most deprived) for IMD quintile; 23 Jan 2020 to 30 Jun 2020 for Recruiting Months.  **Abbreviations:** CI, confidence interval; CVD, cardiovascular disease; IMD, index of multiple deprivation; I(age^2), main effect and the second order interaction of age. | | | | | | |

Supplementary table 13. Number of events and age-standardised IR of in 28-day mortality and 30-day CVD (100,000 population/year) in Wales by sex and time of COVID-19 diagnosis in each 6 High-level from 6 High-level ethnic groups and 10 ethnic codes. Estimates are reported with their 95% confidence intervals. Abbreviations: Age-std, age-standardised; CI, confidence interval; CVD, Cardiovascular disease.

| **Outcome** | **Ethnic classification** | **Ethnic code** | **Period of COVID-19 diagnosis** | **Men results** | | | | **Women results** | | | |
| --- | --- | --- | --- | --- | --- | --- | --- | --- | --- | --- | --- |
|  |  |  |  | **Number of events** | **Age-std IR** | **Low 95%CI** | **High 95%CI** | **Number of events** | **Age-std IR** | **Low 95%CI** | **High 95%CI** |
| High-level groups | 28-day mortality | Asian or Asian British | 23 Jan 2020 to 30 Jun 2020 | <10 | 117.09 | 97.67 | 140.22 | <10 | 21.81 | 13.79 | 32.10 |
| High-level groups | 28-day mortality | Black or Black British | 23 Jan 2020 to 30 Jun 2020 | <10 | 236.25 | 207.79 | 268.11 | <10 | 125.56 | 104.96 | 148.93 |
| High-level groups | 28-day mortality | Mixed | 23 Jan 2020 to 30 Jun 2020 | <10 | 1,336.34 | 1,266.29 | 1,409.61 | <10 | 100.18 | 82.27 | 121.63 |
| High-level groups | 28-day mortality | Other Ethnic Group | 23 Jan 2020 to 30 Jun 2020 | <10 | 23.51 | 15.38 | 34.51 | <10 | 52.24 | 39.70 | 68.19 |
| High-level groups | 28-day mortality | Unknown | 23 Jan 2020 to 30 Jun 2020 | 100 | 421.03 | 338.92 | 503.14 | 85 | 278.57 | 247.28 | 312.75 |
| High-level groups | 28-day mortality | White | 23 Jan 2020 to 30 Jun 2020 | 790 | 186.29 | 173.30 | 199.28 | 650 | 112.17 | 103.53 | 120.80 |
| High-level groups | 28-day mortality | Asian or Asian British | 01 Jul 2020 to 31 Dec 2020 | 20 | 74.09 | 58.63 | 92.45 | 15 | 78.19 | 62.27 | 97.01 |
| High-level groups | 28-day mortality | Black or Black British | 01 Jul 2020 to 31 Dec 2020 | <10 | 28.14 | 19.42 | 40.47 | <10 | 20.38 | 13.00 | 30.89 |
| High-level groups | 28-day mortality | Mixed | 01 Jul 2020 to 31 Dec 2020 | <10 | 173.84 | 149.11 | 200.79 | <10 | 31.77 | 21.89 | 44.00 |
| High-level groups | 28-day mortality | Other Ethnic Group | 01 Jul 2020 to 31 Dec 2020 | <10 | 32.87 | 22.72 | 45.17 | <10 | 34.70 | 24.38 | 47.51 |
| High-level groups | 28-day mortality | Unknown | 01 Jul 2020 to 31 Dec 2020 | 160 | 193.47 | 163.30 | 223.64 | 120 | 115.62 | 95.02 | 136.22 |
| High-level groups | 28-day mortality | White | 01 Jul 2020 to 31 Dec 2020 | 1490 | 76.08 | 72.21 | 79.94 | 1235 | 43.02 | 40.61 | 45.42 |
| High-level groups | 28-day mortality | Asian or Asian British | 01 Jan 2021 to 30 Jun 2021 | 35 | 144.69 | 122.53 | 169.74 | 30 | 107.43 | 88.53 | 129.22 |
| High-level groups | 28-day mortality | Black or Black British | 01 Jan 2021 to 30 Jun 2021 | <10 | 177.31 | 152.81 | 205.08 | <10 | 73.18 | 58.11 | 91.79 |
| High-level groups | 28-day mortality | Mixed | 01 Jan 2021 to 30 Jun 2021 | <10 | 8.56 | 4.12 | 15.76 | <10 | 17.01 | 10.67 | 27.22 |
| High-level groups | 28-day mortality | Other Ethnic Group | 01 Jan 2021 to 30 Jun 2021 | <10 | 49.50 | 37.11 | 64.78 | 0 | - | - | 3.69 |
| High-level groups | 28-day mortality | Unknown | 01 Jan 2021 to 30 Jun 2021 | 60 | 228.61 | 200.40 | 259.71 | 70 | 156.22 | 133.14 | 182.19 |
| High-level groups | 28-day mortality | White | 01 Jan 2021 to 30 Jun 2021 | 760 | 83.13 | 77.22 | 89.03 | 690 | 48.72 | 45.08 | 52.35 |
| High-level groups | 28-day mortality | Asian or Asian British | 01 Jul 2021 to 31 Dec 2021 | 25 | 54.81 | 41.70 | 70.81 | 20 | 40.21 | 29.18 | 54.14 |
| High-level groups | 28-day mortality | Black or Black British | 01 Jul 2021 to 31 Dec 2021 | <10 | 30.52 | 21.06 | 42.83 | 0 | - | - | 3.69 |
| High-level groups | 28-day mortality | Mixed | 01 Jul 2021 to 31 Dec 2021 | <10 | 54.89 | 41.43 | 70.46 | 0 | - | - | 3.69 |
| High-level groups | 28-day mortality | Other Ethnic Group | 01 Jul 2021 to 31 Dec 2021 | <10 | 9.92 | 4.80 | 17.09 | <10 | 36.10 | 26.05 | 49.84 |
| High-level groups | 28-day mortality | Unknown | 01 Jul 2021 to 31 Dec 2021 | 55 | 54.73 | 41.63 | 70.72 | 30 | 28.66 | 19.55 | 40.66 |
| High-level groups | 28-day mortality | White | 01 Jul 2021 to 31 Dec 2021 | 710 | 22.37 | 20.73 | 24.02 | 530 | 13.73 | 12.56 | 14.90 |
| High-level groups | 28-day mortality | Asian or Asian British | 01 Jan 2022 to 01 Apr 2022 | 20 | 98.59 | 80.54 | 119.53 | 15 | 33.07 | 23.18 | 45.84 |
| High-level groups | 28-day mortality | Black or Black British | 01 Jan 2022 to 01 Apr 2022 | <10 | 2,492.06 | 2,396.09 | 2,591.81 | <10 | 8.58 | 4.12 | 15.76 |
| High-level groups | 28-day mortality | Mixed | 01 Jan 2022 to 01 Apr 2022 | <10 | 10.98 | 5.49 | 18.39 | <10 | 10.56 | 5.49 | 18.39 |
| High-level groups | 28-day mortality | Other Ethnic Group | 01 Jan 2022 to 01 Apr 2022 | <10 | 8.78 | 4.12 | 15.76 | 0 | - | - | 3.69 |
| High-level groups | 28-day mortality | Unknown | 01 Jan 2022 to 01 Apr 2022 | 55 | 36.52 | 26.07 | 49.87 | 55 | 30.04 | 20.68 | 42.28 |
| High-level groups | 28-day mortality | White | 01 Jan 2022 to 01 Apr 2022 | 525 | 21.96 | 20.08 | 23.84 | 500 | 16.05 | 14.64 | 17.46 |
| 10 ethnic codes | 28-day mortality | Bangladeshi | 23 Jan 2020 to 30 Jun 2020 | <10 | 66.94 | 51.92 | 83.97 | 0 | - | - | 3.69 |
| 10 ethnic codes | 28-day mortality | Black African | 23 Jan 2020 to 30 Jun 2020 | <10 | 196.03 | 170.45 | 225.44 | <10 | 16.44 | 9.90 | 25.98 |
| 10 ethnic codes | 28-day mortality | Black Caribbean | 23 Jan 2020 to 30 Jun 2020 | <10 | 82.33 | 66.11 | 101.78 | <10 | 864.47 | 808.31 | 923.59 |
| 10 ethnic codes | 28-day mortality | Chinese | 23 Jan 2020 to 30 Jun 2020 | 0 | - | - | 3.69 | 0 | - | - | 3.69 |
| 10 ethnic codes | 28-day mortality | Indian | 23 Jan 2020 to 30 Jun 2020 | <10 | 50.40 | 37.97 | 65.92 | <10 | 24.95 | 16.18 | 35.71 |
| 10 ethnic codes | 28-day mortality | Mixed | 23 Jan 2020 to 30 Jun 2020 | <10 | 1,336.34 | 1,266.29 | 1,409.61 | <10 | 100.18 | 82.27 | 121.63 |
| 10 ethnic codes | 28-day mortality | Other Ethnic Group | 23 Jan 2020 to 30 Jun 2020 | <10 | 392.14 | 355.10 | 432.79 | <10 | 40.16 | 29.42 | 54.47 |
| 10 ethnic codes | 28-day mortality | Pakistani | 23 Jan 2020 to 30 Jun 2020 | <10 | 22.25 | 14.58 | 33.31 | <10 | 481.84 | 439.93 | 525.97 |
| 10 ethnic codes | 28-day mortality | Unknown | 23 Jan 2020 to 30 Jun 2020 | 100 | 421.03 | 338.92 | 503.14 | 85 | 278.57 | 247.28 | 312.75 |
| 10 ethnic codes | 28-day mortality | White | 23 Jan 2020 to 30 Jun 2020 | 790 | 186.29 | 173.30 | 199.28 | 650 | 112.17 | 103.53 | 120.80 |
| 10 ethnic codes | 28-day mortality | Bangladeshi | 01 Jul 2020 to 31 Dec 2020 | <10 | 15.32 | 9.15 | 24.74 | <10 | 88.01 | 71.47 | 108.42 |
| 10 ethnic codes | 28-day mortality | Black African | 01 Jul 2020 to 31 Dec 2020 | <10 | 117.62 | 97.67 | 140.22 | <10 | 28.55 | 19.42 | 40.47 |
| 10 ethnic codes | 28-day mortality | Black Caribbean | 01 Jul 2020 to 31 Dec 2020 | <10 | 19.39 | 12.22 | 29.67 | 0 | - | - | 3.69 |
| 10 ethnic codes | 28-day mortality | Chinese | 01 Jul 2020 to 31 Dec 2020 | 0 | - | - | 3.69 | <10 | 344.29 | 309.55 | 382.34 |
| 10 ethnic codes | 28-day mortality | Indian | 01 Jul 2020 to 31 Dec 2020 | <10 | 79.06 | 63.44 | 98.46 | <10 | 274.44 | 243.46 | 308.44 |
| 10 ethnic codes | 28-day mortality | Mixed | 01 Jul 2020 to 31 Dec 2020 | <10 | 173.84 | 149.11 | 200.79 | <10 | 31.77 | 21.89 | 44.00 |
| 10 ethnic codes | 28-day mortality | Other Ethnic Group | 01 Jul 2020 to 31 Dec 2020 | 10 | 48.36 | 36.13 | 63.48 | 10 | 59.73 | 45.98 | 76.37 |
| 10 ethnic codes | 28-day mortality | Pakistani | 01 Jul 2020 to 31 Dec 2020 | <10 | 314.89 | 281.17 | 350.72 | 0 | - | - | 3.69 |
| 10 ethnic codes | 28-day mortality | Unknown | 01 Jul 2020 to 31 Dec 2020 | 160 | 193.47 | 163.30 | 223.64 | 120 | 115.62 | 95.02 | 136.22 |
| 10 ethnic codes | 28-day mortality | White | 01 Jul 2020 to 31 Dec 2020 | 1490 | 76.08 | 72.21 | 79.94 | 1235 | 43.02 | 40.61 | 45.42 |
| 10 ethnic codes | 28-day mortality | Bangladeshi | 01 Jan 2021 to 30 Jun 2021 | 25 | 189.79 | 164.21 | 218.26 | 25 | 144.23 | 122.11 | 169.24 |
| 10 ethnic codes | 28-day mortality | Black African | 01 Jan 2021 to 30 Jun 2021 | <10 | 185.57 | 160.23 | 213.67 | <10 | 73.18 | 58.11 | 91.79 |
| 10 ethnic codes | 28-day mortality | Black Caribbean | 01 Jan 2021 to 30 Jun 2021 | 0 | - | - | 3.69 | 0 | - | - | 3.69 |
| 10 ethnic codes | 28-day mortality | Chinese | 01 Jan 2021 to 30 Jun 2021 | 0 | - | - | 3.69 | <10 | 102.91 | 84.07 | 123.82 |
| 10 ethnic codes | 28-day mortality | Indian | 01 Jan 2021 to 30 Jun 2021 | <10 | 4.54 | 1.62 | 10.24 | 0 | - | - | 3.69 |
| 10 ethnic codes | 28-day mortality | Mixed | 01 Jan 2021 to 30 Jun 2021 | <10 | 8.56 | 4.12 | 15.76 | <10 | 17.01 | 10.67 | 27.22 |
| 10 ethnic codes | 28-day mortality | Other Ethnic Group | 01 Jan 2021 to 30 Jun 2021 | <10 | 76.50 | 60.77 | 95.13 | <10 | 7.46 | 3.45 | 14.42 |
| 10 ethnic codes | 28-day mortality | Pakistani | 01 Jan 2021 to 30 Jun 2021 | <10 | 22.87 | 14.58 | 33.31 | <10 | 46.68 | 34.53 | 61.36 |
| 10 ethnic codes | 28-day mortality | Unknown | 01 Jan 2021 to 30 Jun 2021 | 60 | 228.61 | 200.40 | 259.71 | 70 | 156.22 | 133.14 | 182.19 |
| 10 ethnic codes | 28-day mortality | White | 01 Jan 2021 to 30 Jun 2021 | 760 | 83.13 | 77.22 | 89.03 | 690 | 48.72 | 45.08 | 52.35 |
| 10 ethnic codes | 28-day mortality | Bangladeshi | 01 Jul 2021 to 31 Dec 2021 | 20 | 112.45 | 93.08 | 134.72 | 15 | 49.29 | 36.93 | 64.55 |
| 10 ethnic codes | 28-day mortality | Black African | 01 Jul 2021 to 31 Dec 2021 | <10 | 29.51 | 20.24 | 41.65 | 0 | - | - | 3.69 |
| 10 ethnic codes | 28-day mortality | Black Caribbean | 01 Jul 2021 to 31 Dec 2021 | <10 | 40.15 | 29.42 | 54.47 | 0 | - | - | 3.69 |
| 10 ethnic codes | 28-day mortality | Chinese | 01 Jul 2021 to 31 Dec 2021 | 0 | - | - | 3.69 | 0 | - | - | 3.69 |
| 10 ethnic codes | 28-day mortality | Indian | 01 Jul 2021 to 31 Dec 2021 | <10 | 31.02 | 21.89 | 44.00 | 0 | - | - | 3.69 |
| 10 ethnic codes | 28-day mortality | Mixed | 01 Jul 2021 to 31 Dec 2021 | <10 | 54.89 | 41.43 | 70.46 | 0 | - | - | 3.69 |
| 10 ethnic codes | 28-day mortality | Other Ethnic Group | 01 Jul 2021 to 31 Dec 2021 | <10 | 9.69 | 4.80 | 17.09 | <10 | 37.32 | 26.89 | 51.00 |
| 10 ethnic codes | 28-day mortality | Pakistani | 01 Jul 2021 to 31 Dec 2021 | <10 | 57.04 | 44.04 | 73.85 | <10 | 18.01 | 11.44 | 28.45 |
| 10 ethnic codes | 28-day mortality | Unknown | 01 Jul 2021 to 31 Dec 2021 | 55 | 54.73 | 41.63 | 70.72 | 30 | 28.66 | 19.55 | 40.66 |
| 10 ethnic codes | 28-day mortality | White | 01 Jul 2021 to 31 Dec 2021 | 710 | 22.37 | 20.73 | 24.02 | 530 | 13.73 | 12.56 | 14.90 |
| 10 ethnic codes | 28-day mortality | Bangladeshi | 01 Jan 2022 to 01 Apr 2022 | 15 | 123.76 | 103.37 | 147.04 | 10 | 44.80 | 33.07 | 59.41 |
| 10 ethnic codes | 28-day mortality | Black African | 01 Jan 2022 to 01 Apr 2022 | <10 | 227.96 | 199.36 | 258.53 | 0 | - | - | 3.69 |
| 10 ethnic codes | 28-day mortality | Black Caribbean | 01 Jan 2022 to 01 Apr 2022 | <10 | 2,607.14 | 2,508.86 | 2,709.04 | <10 | 20.58 | 13.00 | 30.89 |
| 10 ethnic codes | 28-day mortality | Chinese | 01 Jan 2022 to 01 Apr 2022 | 0 | - | - | 3.69 | 0 | - | - | 3.69 |
| 10 ethnic codes | 28-day mortality | Indian | 01 Jan 2022 to 01 Apr 2022 | 0 | - | - | 3.69 | <10 | 208.74 | 181.62 | 238.27 |
| 10 ethnic codes | 28-day mortality | Mixed | 01 Jan 2022 to 01 Apr 2022 | <10 | 10.98 | 5.49 | 18.39 | <10 | 10.56 | 5.49 | 18.39 |
| 10 ethnic codes | 28-day mortality | Other Ethnic Group | 01 Jan 2022 to 01 Apr 2022 | <10 | 7.81 | 3.45 | 14.42 | <10 | 11.14 | 6.20 | 19.68 |
| 10 ethnic codes | 28-day mortality | Pakistani | 01 Jan 2022 to 01 Apr 2022 | <10 | 61.03 | 47.54 | 78.36 | 0 | - | - | 3.69 |
| 10 ethnic codes | 28-day mortality | Unknown | 01 Jan 2022 to 01 Apr 2022 | 55 | 36.52 | 26.07 | 49.87 | 55 | 30.04 | 20.68 | 42.28 |
| 10 ethnic codes | 28-day mortality | White | 01 Jan 2022 to 01 Apr 2022 | 525 | 21.96 | 20.08 | 23.84 | 500 | 16.05 | 14.64 | 17.46 |
| High-level groups | 30-day CVD | Asian or Asian British | 23 Jan 2020 to 30 Jun 2020 | <10 | 57.90 | 44.04 | 73.85 | <10 | 23.01 | 15.38 | 34.51 |
| High-level groups | 30-day CVD | Black or Black British | 23 Jan 2020 to 30 Jun 2020 | 0 | - | - | 3.69 | <10 | 23.43 | 15.38 | 34.51 |
| High-level groups | 30-day CVD | Mixed | 23 Jan 2020 to 30 Jun 2020 | 0 | - | - | 3.69 | <10 | 25.44 | 16.98 | 36.91 |
| High-level groups | 30-day CVD | Other Ethnic Group | 23 Jan 2020 to 30 Jun 2020 | 0 | - | - | 3.69 | 0 | - | - | 3.69 |
| High-level groups | 30-day CVD | Unknown | 23 Jan 2020 to 30 Jun 2020 | <10 | 11.12 | 6.20 | 19.68 | <10 | 1.95 | 0.24 | 5.57 |
| High-level groups | 30-day CVD | White | 23 Jan 2020 to 30 Jun 2020 | 85 | 23.03 | 14.99 | 33.94 | 75 | 13.91 | 7.95 | 22.75 |
| High-level groups | 30-day CVD | Asian or Asian British | 01 Jul 2020 to 31 Dec 2020 | 10 | 27.22 | 18.37 | 38.95 | <10 | 13.54 | 7.65 | 22.23 |
| High-level groups | 30-day CVD | Black or Black British | 01 Jul 2020 to 31 Dec 2020 | <10 | 20.18 | 13.00 | 30.89 | 0 | - | - | 3.69 |
| High-level groups | 30-day CVD | Mixed | 01 Jul 2020 to 31 Dec 2020 | <10 | 56.78 | 43.17 | 72.72 | 0 | - | - | 3.69 |
| High-level groups | 30-day CVD | Other Ethnic Group | 01 Jul 2020 to 31 Dec 2020 | <10 | 19.05 | 12.22 | 29.67 | <10 | 8.27 | 4.12 | 15.76 |
| High-level groups | 30-day CVD | Unknown | 01 Jul 2020 to 31 Dec 2020 | 20 | 24.27 | 15.99 | 35.43 | <10 | 5.54 | 2.20 | 11.67 |
| High-level groups | 30-day CVD | White | 01 Jul 2020 to 31 Dec 2020 | 290 | 12.54 | 11.10 | 13.98 | 200 | 7.31 | 6.30 | 8.32 |
| High-level groups | 30-day CVD | Asian or Asian British | 01 Jan 2021 to 30 Jun 2021 | <10 | 7.64 | 3.45 | 14.42 | <10 | 9.69 | 4.80 | 17.09 |
| High-level groups | 30-day CVD | Black or Black British | 01 Jan 2021 to 30 Jun 2021 | <10 | 13.72 | 7.65 | 22.23 | 0 | - | - | 3.69 |
| High-level groups | 30-day CVD | Mixed | 01 Jan 2021 to 30 Jun 2021 | <10 | 18.84 | 11.44 | 28.45 | 0 | - | - | 3.69 |
| High-level groups | 30-day CVD | Other Ethnic Group | 01 Jan 2021 to 30 Jun 2021 | <10 | 40.90 | 29.42 | 54.47 | 0 | - | - | 3.69 |
| High-level groups | 30-day CVD | Unknown | 01 Jan 2021 to 30 Jun 2021 | <10 | 40.21 | 29.42 | 54.47 | <10 | 17.82 | 10.67 | 27.22 |
| High-level groups | 30-day CVD | White | 01 Jan 2021 to 30 Jun 2021 | 170 | 17.88 | 15.20 | 20.56 | 150 | 12.30 | 10.34 | 14.26 |
| High-level groups | 30-day CVD | Asian or Asian British | 01 Jul 2021 to 31 Dec 2021 | 10 | 15.36 | 9.03 | 24.57 | 10 | 15.36 | 9.04 | 24.57 |
| High-level groups | 30-day CVD | Black or Black British | 01 Jul 2021 to 31 Dec 2021 | <10 | 13.85 | 7.65 | 22.23 | <10 | 45.86 | 33.68 | 60.21 |
| High-level groups | 30-day CVD | Mixed | 01 Jul 2021 to 31 Dec 2021 | <10 | 5.53 | 2.20 | 11.67 | <10 | 1.60 | 0.24 | 5.57 |
| High-level groups | 30-day CVD | Other Ethnic Group | 01 Jul 2021 to 31 Dec 2021 | <10 | 7.21 | 3.45 | 14.42 | 0 | - | - | 3.69 |
| High-level groups | 30-day CVD | Unknown | 01 Jul 2021 to 31 Dec 2021 | 15 | 9.60 | 4.86 | 17.22 | 10 | 6.64 | 2.89 | 13.25 |
| High-level groups | 30-day CVD | White | 01 Jul 2021 to 31 Dec 2021 | 360 | 7.96 | 7.14 | 8.79 | 220 | 4.43 | 3.85 | 5.01 |
| High-level groups | 30-day CVD | Asian or Asian British | 01 Jan 2022 to 01 Apr 2022 | <10 | 16.82 | 9.90 | 25.98 | <10 | 12.68 | 6.92 | 20.96 |
| High-level groups | 30-day CVD | Black or Black British | 01 Jan 2022 to 01 Apr 2022 | <10 | 3.85 | 1.09 | 8.77 | <10 | 13.11 | 7.65 | 22.23 |
| High-level groups | 30-day CVD | Mixed | 01 Jan 2022 to 01 Apr 2022 | 0 | - | - | 3.69 | <10 | 4.53 | 1.62 | 10.24 |
| High-level groups | 30-day CVD | Other Ethnic Group | 01 Jan 2022 to 01 Apr 2022 | <10 | 31.77 | 21.89 | 44.00 | <10 | 82.33 | 66.11 | 101.78 |
| High-level groups | 30-day CVD | Unknown | 01 Jan 2022 to 01 Apr 2022 | 25 | 15.40 | 9.06 | 24.61 | 20 | 10.49 | 5.48 | 18.38 |
| High-level groups | 30-day CVD | White | 01 Jan 2022 to 01 Apr 2022 | 410 | 16.62 | 15.00 | 18.23 | 280 | 8.95 | 7.90 | 10.00 |
| 10 ethnic codes | 30-day CVD | Bangladeshi | 23 Jan 2020 to 30 Jun 2020 | <10 | 83.07 | 67.00 | 102.89 | 0 | - | - | 3.69 |
| 10 ethnic codes | 30-day CVD | Black African | 23 Jan 2020 to 30 Jun 2020 | 0 | - | - | 3.69 | 0 | - | - | 3.69 |
| 10 ethnic codes | 30-day CVD | Black Caribbean | 23 Jan 2020 to 30 Jun 2020 | 0 | - | - | 3.69 | <10 | 274.44 | 243.46 | 308.44 |
| 10 ethnic codes | 30-day CVD | Chinese | 23 Jan 2020 to 30 Jun 2020 | 0 | - | - | 3.69 | 0 | - | - | 3.69 |
| 10 ethnic codes | 30-day CVD | Indian | 23 Jan 2020 to 30 Jun 2020 | <10 | 22.56 | 14.58 | 33.31 | 0 | - | - | 3.69 |
| 10 ethnic codes | 30-day CVD | Mixed | 23 Jan 2020 to 30 Jun 2020 | 0 | - | - | 3.69 | <10 | 25.44 | 16.98 | 36.91 |
| 10 ethnic codes | 30-day CVD | Other Ethnic Group | 23 Jan 2020 to 30 Jun 2020 | <10 | 88.53 | 71.47 | 108.42 | <10 | 1.28 | 0.24 | 5.57 |
| 10 ethnic codes | 30-day CVD | Pakistani | 23 Jan 2020 to 30 Jun 2020 | <10 | 23.52 | 15.38 | 34.51 | <10 | 177.58 | 152.81 | 205.08 |
| 10 ethnic codes | 30-day CVD | Unknown | 23 Jan 2020 to 30 Jun 2020 | <10 | 11.12 | 6.20 | 19.68 | <10 | 1.95 | 0.24 | 5.57 |
| 10 ethnic codes | 30-day CVD | White | 23 Jan 2020 to 30 Jun 2020 | 85 | 23.03 | 14.99 | 33.94 | 75 | 13.91 | 7.95 | 22.75 |
| 10 ethnic codes | 30-day CVD | Bangladeshi | 01 Jul 2020 to 31 Dec 2020 | <10 | 7.11 | 3.45 | 14.42 | <10 | 22.68 | 14.58 | 33.31 |
| 10 ethnic codes | 30-day CVD | Black African | 01 Jul 2020 to 31 Dec 2020 | 0 | - | - | 3.69 | 0 | - | - | 3.69 |
| 10 ethnic codes | 30-day CVD | Black Caribbean | 01 Jul 2020 to 31 Dec 2020 | <10 | 171.52 | 147.26 | 198.64 | 0 | - | - | 3.69 |
| 10 ethnic codes | 30-day CVD | Chinese | 01 Jul 2020 to 31 Dec 2020 | <10 | 823.31 | 768.69 | 881.20 | <10 | 77.41 | 61.66 | 96.24 |
| 10 ethnic codes | 30-day CVD | Indian | 01 Jul 2020 to 31 Dec 2020 | <10 | 3.34 | 1.09 | 8.77 | <10 | 2.45 | 0.62 | 7.23 |
| 10 ethnic codes | 30-day CVD | Mixed | 01 Jul 2020 to 31 Dec 2020 | <10 | 56.78 | 43.17 | 72.72 | 0 | - | - | 3.69 |
| 10 ethnic codes | 30-day CVD | Other Ethnic Group | 01 Jul 2020 to 31 Dec 2020 | <10 | 12.32 | 6.92 | 20.96 | <10 | 3.46 | 1.09 | 8.77 |
| 10 ethnic codes | 30-day CVD | Pakistani | 01 Jul 2020 to 31 Dec 2020 | <10 | 35.09 | 25.21 | 48.68 | <10 | 25.99 | 16.98 | 36.91 |
| 10 ethnic codes | 30-day CVD | Unknown | 01 Jul 2020 to 31 Dec 2020 | 20 | 24.27 | 15.99 | 35.43 | <10 | 5.54 | 2.20 | 11.67 |
| 10 ethnic codes | 30-day CVD | White | 01 Jul 2020 to 31 Dec 2020 | 290 | 12.54 | 11.10 | 13.98 | 200 | 7.31 | 6.30 | 8.32 |
| 10 ethnic codes | 30-day CVD | Bangladeshi | 01 Jan 2021 to 30 Jun 2021 | 0 | - | - | 3.69 | <10 | 10.13 | 5.49 | 18.39 |
| 10 ethnic codes | 30-day CVD | Black African | 01 Jan 2021 to 30 Jun 2021 | <10 | 9.37 | 4.80 | 17.09 | 0 | - | - | 3.69 |
| 10 ethnic codes | 30-day CVD | Black Caribbean | 01 Jan 2021 to 30 Jun 2021 | <10 | 35.80 | 25.21 | 48.68 | 0 | - | - | 3.69 |
| 10 ethnic codes | 30-day CVD | Chinese | 01 Jan 2021 to 30 Jun 2021 | 0 | - | - | 3.69 | 0 | - | - | 3.69 |
| 10 ethnic codes | 30-day CVD | Indian | 01 Jan 2021 to 30 Jun 2021 | <10 | 25.84 | 16.98 | 36.91 | <10 | 9.44 | 4.80 | 17.09 |
| 10 ethnic codes | 30-day CVD | Mixed | 01 Jan 2021 to 30 Jun 2021 | <10 | 18.84 | 11.44 | 28.45 | 0 | - | - | 3.69 |
| 10 ethnic codes | 30-day CVD | Other Ethnic Group | 01 Jan 2021 to 30 Jun 2021 | <10 | 30.33 | 21.06 | 42.83 | <10 | 1.96 | 0.24 | 5.57 |
| 10 ethnic codes | 30-day CVD | Pakistani | 01 Jan 2021 to 30 Jun 2021 | 0 | - | - | 3.69 | <10 | 6.04 | 2.81 | 13.06 |
| 10 ethnic codes | 30-day CVD | Unknown | 01 Jan 2021 to 30 Jun 2021 | <10 | 40.21 | 29.42 | 54.47 | <10 | 17.82 | 10.67 | 27.22 |
| 10 ethnic codes | 30-day CVD | White | 01 Jan 2021 to 30 Jun 2021 | 170 | 17.88 | 15.20 | 20.56 | 150 | 12.30 | 10.34 | 14.26 |
| 10 ethnic codes | 30-day CVD | Bangladeshi | 01 Jul 2021 to 31 Dec 2021 | <10 | 21.08 | 13.79 | 32.10 | <10 | 25.69 | 16.98 | 36.91 |
| 10 ethnic codes | 30-day CVD | Black African | 01 Jul 2021 to 31 Dec 2021 | 0 | - | - | 3.69 | <10 | 112.45 | 93.13 | 134.77 |
| 10 ethnic codes | 30-day CVD | Black Caribbean | 01 Jul 2021 to 31 Dec 2021 | <10 | 18.70 | 11.44 | 28.45 | 0 | - | - | 3.69 |
| 10 ethnic codes | 30-day CVD | Chinese | 01 Jul 2021 to 31 Dec 2021 | 0 | - | - | 3.69 | 0 | - | - | 3.69 |
| 10 ethnic codes | 30-day CVD | Indian | 01 Jul 2021 to 31 Dec 2021 | 0 | - | - | 3.69 | <10 | 5.21 | 2.20 | 11.67 |
| 10 ethnic codes | 30-day CVD | Mixed | 01 Jul 2021 to 31 Dec 2021 | <10 | 5.53 | 2.20 | 11.67 | <10 | 1.60 | 0.24 | 5.57 |
| 10 ethnic codes | 30-day CVD | Other Ethnic Group | 01 Jul 2021 to 31 Dec 2021 | <10 | 13.06 | 7.65 | 22.23 | <10 | 0.80 | 0.03 | 3.69 |
| 10 ethnic codes | 30-day CVD | Pakistani | 01 Jul 2021 to 31 Dec 2021 | <10 | 27.34 | 18.61 | 39.28 | <10 | 18.95 | 11.44 | 28.45 |
| 10 ethnic codes | 30-day CVD | Unknown | 01 Jul 2021 to 31 Dec 2021 | 15 | 9.60 | 4.86 | 17.22 | 10 | 6.64 | 2.89 | 13.25 |
| 10 ethnic codes | 30-day CVD | White | 01 Jul 2021 to 31 Dec 2021 | 360 | 7.96 | 7.14 | 8.79 | 220 | 4.43 | 3.85 | 5.01 |
| 10 ethnic codes | 30-day CVD | Bangladeshi | 01 Jan 2022 to 01 Apr 2022 | <10 | 33.99 | 23.55 | 46.34 | <10 | 12.13 | 6.92 | 20.96 |
| 10 ethnic codes | 30-day CVD | Black African | 01 Jan 2022 to 01 Apr 2022 | 0 | - | - | 3.69 | <10 | 19.54 | 12.22 | 29.67 |
| 10 ethnic codes | 30-day CVD | Black Caribbean | 01 Jan 2022 to 01 Apr 2022 | 0 | - | - | 3.69 | <10 | 9.36 | 4.80 | 17.09 |
| 10 ethnic codes | 30-day CVD | Chinese | 01 Jan 2022 to 01 Apr 2022 | 0 | - | - | 3.69 | 0 | - | - | 3.69 |
| 10 ethnic codes | 30-day CVD | Indian | 01 Jan 2022 to 01 Apr 2022 | <10 | 3.30 | 1.09 | 8.77 | <10 | 16.47 | 9.90 | 25.98 |
| 10 ethnic codes | 30-day CVD | Mixed | 01 Jan 2022 to 01 Apr 2022 | 0 | - | - | 3.69 | <10 | 4.53 | 1.62 | 10.24 |
| 10 ethnic codes | 30-day CVD | Other Ethnic Group | 01 Jan 2022 to 01 Apr 2022 | <10 | 18.24 | 11.44 | 28.45 | <10 | 35.41 | 25.21 | 48.68 |
| 10 ethnic codes | 30-day CVD | Pakistani | 01 Jan 2022 to 01 Apr 2022 | <10 | 16.12 | 9.90 | 25.98 | <10 | 27.32 | 18.61 | 39.28 |
| 10 ethnic codes | 30-day CVD | Unknown | 01 Jan 2022 to 01 Apr 2022 | 25 | 15.40 | 9.06 | 24.61 | 20 | 10.49 | 5.48 | 18.38 |
| 10 ethnic codes | 30-day CVD | White | 01 Jan 2022 to 01 Apr 2022 | 410 | 16.62 | 15.00 | 18.23 | 280 | 8.95 | 7.90 | 10.00 |

Supplementary Table 14. Number of events and age-standardised IR of in 28-day mortality and 30-day CVD (100,000 population/year) in England by sex and time of COVID-19 diagnosis in each 6 High-level categories and 19 NHS ethnicity codes. Estimates are reported with their 95% confidence intervals. Abbreviations: Age-std, age-standardised; CI, confidence interval; CVD, Cardiovascular disease.

| **Outcome** | **Ethnic classification** | **Ethnic code** | **Period of COVID-19 diagnosis** | **Men results** | | | | **Women results** | | | |
| --- | --- | --- | --- | --- | --- | --- | --- | --- | --- | --- | --- |
|  |  |  |  | **Number of events** | **Age-std IR** | **Low 95%CI** | **High 95%CI** | **Number of events** | **Age-std IR** | **Low 95%CI** | **High 95%CI** |
| 28-day mortality | High-level groups | Asian or Asian British | 23 Jan 2020 to 30 Jun 2020 | 1,145 | 239.7 | 225.8 | 253.5 | 640 | 148.4 | 136.9 | 159.9 |
| 28-day mortality | High-level groups | Asian or Asian British | 01 Jul 2020 to 31 Dec 2020 | 1,370 | 85.4 | 80.9 | 89.9 | 860 | 49.4 | 46.1 | 52.7 |
| 28-day mortality | High-level groups | Asian or Asian British | 01 Jan 2021 to 30 Jun 2021 | 1,295 | 96.6 | 91.3 | 101.9 | 840 | 56.3 | 52.5 | 60.1 |
| 28-day mortality | High-level groups | Asian or Asian British | 01 Jul 2021 to 31 Dec 2021 | 510 | 29.8 | 27.2 | 32.4 | 345 | 21.0 | 18.8 | 23.2 |
| 28-day mortality | High-level groups | Asian or Asian British | 01 Jan 2022 to 01 Apr 2022 | 70 | 11.2 | 6.0 | 19.4 | 40 | 5.9 | 2.4 | 12.2 |
| 28-day mortality | High-level groups | Black or Black British | 23 Jan 2020 to 30 Jun 2020 | 720 | 257.5 | 238.7 | 276.3 | 360 | 135.3 | 121.4 | 149.3 |
| 28-day mortality | High-level groups | Black or Black British | 01 Jul 2020 to 31 Dec 2020 | 355 | 86.7 | 77.7 | 95.7 | 250 | 50.5 | 44.2 | 56.8 |
| 28-day mortality | High-level groups | Black or Black British | 01 Jan 2021 to 30 Jun 2021 | 515 | 105.0 | 96.0 | 114.1 | 350 | 54.4 | 48.7 | 60.1 |
| 28-day mortality | High-level groups | Black or Black British | 01 Jul 2021 to 31 Dec 2021 | 270 | 36.8 | 32.4 | 41.1 | 240 | 24.6 | 21.5 | 27.7 |
| 28-day mortality | High-level groups | Black or Black British | 01 Jan 2022 to 01 Apr 2022 | 25 | 9.7 | 4.9 | 17.3 | 15 | 4.1 | 1.4 | 9.7 |
| 28-day mortality | High-level groups | Mixed | 23 Jan 2020 to 30 Jun 2020 | 155 | 256.6 | 216.2 | 297.0 | 105 | 165.8 | 134.2 | 197.3 |
| 28-day mortality | High-level groups | Mixed | 01 Jul 2020 to 31 Dec 2020 | 105 | 92.7 | 74.8 | 110.5 | 65 | 41.5 | 30.3 | 55.7 |
| 28-day mortality | High-level groups | Mixed | 01 Jan 2021 to 30 Jun 2021 | 110 | 94.8 | 77.2 | 112.4 | 85 | 58.7 | 45.1 | 75.2 |
| 28-day mortality | High-level groups | Mixed | 01 Jul 2021 to 31 Dec 2021 | 70 | 32.3 | 22.6 | 45.0 | 65 | 30.9 | 21.4 | 43.2 |
| 28-day mortality | High-level groups | Mixed | 01 Jan 2022 to 01 Apr 2022 | <10 | 8.3 | 4.1 | 15.8 | <10 | 4.4 | 1.6 | 10.2 |
| 28-day mortality | High-level groups | Other Ethnic Group | 23 Jan 2020 to 30 Jun 2020 | 220 | 245.3 | 212.7 | 277.9 | 145 | 163.4 | 136.7 | 190.0 |
| 28-day mortality | High-level groups | Other Ethnic Group | 01 Jul 2020 to 31 Dec 2020 | 135 | 72.3 | 60.0 | 84.6 | 85 | 40.4 | 29.3 | 54.4 |
| 28-day mortality | High-level groups | Other Ethnic Group | 01 Jan 2021 to 30 Jun 2021 | 150 | 92.8 | 78.0 | 107.6 | 105 | 51.5 | 41.8 | 61.3 |
| 28-day mortality | High-level groups | Other Ethnic Group | 01 Jul 2021 to 31 Dec 2021 | 70 | 29.4 | 20.1 | 41.5 | 50 | 19.5 | 12.2 | 29.7 |
| 28-day mortality | High-level groups | Other Ethnic Group | 01 Jan 2022 to 01 Apr 2022 | <10 | 4.3 | 1.6 | 10.2 | 10 | 7.5 | 3.4 | 14.4 |
| 28-day mortality | High-level groups | Unknown | 23 Jan 2020 to 30 Jun 2020 | 400 | 201.5 | 181.8 | 221.3 | 245 | 79.4 | 69.5 | 89.4 |
| 28-day mortality | High-level groups | Unknown | 01 Jul 2020 to 31 Dec 2020 | 305 | 52.4 | 46.5 | 58.3 | 270 | 30.5 | 26.9 | 34.2 |
| 28-day mortality | High-level groups | Unknown | 01 Jan 2021 to 30 Jun 2021 | 385 | 77.7 | 69.9 | 85.5 | 365 | 48.1 | 43.2 | 53.1 |
| 28-day mortality | High-level groups | Unknown | 01 Jul 2021 to 31 Dec 2021 | 195 | 19.6 | 16.8 | 22.3 | 160 | 13.1 | 11.1 | 15.1 |
| 28-day mortality | High-level groups | Unknown | 01 Jan 2022 to 01 Apr 2022 | 30 | 5.7 | 2.3 | 11.9 | 15 | 2.4 | 0.6 | 7.0 |
| 28-day mortality | High-level groups | White | 23 Jan 2020 to 30 Jun 2020 | 12,960 | 183.5 | 180.3 | 186.7 | 9,845 | 103.8 | 101.7 | 105.8 |
| 28-day mortality | High-level groups | White | 01 Jul 2020 to 31 Dec 2020 | 14,210 | 68.7 | 67.6 | 69.8 | 11,825 | 39.9 | 39.1 | 40.6 |
| 28-day mortality | High-level groups | White | 01 Jan 2021 to 30 Jun 2021 | 12,040 | 78.5 | 77.1 | 79.9 | 11,155 | 46.5 | 45.6 | 47.3 |
| 28-day mortality | High-level groups | White | 01 Jul 2021 to 31 Dec 2021 | 6,225 | 19.5 | 19.0 | 20.0 | 4,470 | 11.7 | 11.3 | 12.0 |
| 28-day mortality | High-level groups | White | 01 Jan 2022 to 01 Apr 2022 | 855 | 5.9 | 5.5 | 6.3 | 700 | 3.3 | 3.1 | 3.6 |
| 28-day mortality | NHS ethnicity codes | African | 23 Jan 2020 to 30 Jun 2020 | 270 | 245.4 | 216.0 | 274.8 | 115 | 135.1 | 110.6 | 159.6 |
| 28-day mortality | NHS ethnicity codes | African | 01 Jul 2020 to 31 Dec 2020 | 115 | 88.9 | 72.7 | 105.1 | 85 | 47.6 | 35.4 | 62.6 |
| 28-day mortality | NHS ethnicity codes | African | 01 Jan 2021 to 30 Jun 2021 | 200 | 124.9 | 107.5 | 142.3 | 110 | 59.6 | 48.4 | 70.8 |
| 28-day mortality | NHS ethnicity codes | African | 01 Jul 2021 to 31 Dec 2021 | 60 | 22.2 | 14.3 | 32.9 | 85 | 29.6 | 20.3 | 41.8 |
| 28-day mortality | NHS ethnicity codes | African | 01 Jan 2022 to 01 Apr 2022 | <10 | 9.6 | 2.9 | 16.2 | <10 | 2.1 | 0.0 | 4.2 |
| 28-day mortality | NHS ethnicity codes | Any other Asian background | 23 Jan 2020 to 30 Jun 2020 | 185 | 183.8 | 157.2 | 210.4 | 85 | 99.7 | 81.6 | 120.8 |
| 28-day mortality | NHS ethnicity codes | Any other Asian background | 01 Jul 2020 to 31 Dec 2020 | 195 | 80.2 | 68.9 | 91.5 | 120 | 49.3 | 40.4 | 58.2 |
| 28-day mortality | NHS ethnicity codes | Any other Asian background | 01 Jan 2021 to 30 Jun 2021 | 225 | 103.6 | 90.1 | 117.1 | 140 | 55.9 | 46.7 | 65.2 |
| 28-day mortality | NHS ethnicity codes | Any other Asian background | 01 Jul 2021 to 31 Dec 2021 | 80 | 24.0 | 15.8 | 35.1 | 40 | 14.6 | 8.5 | 23.6 |
| 28-day mortality | NHS ethnicity codes | Any other Asian background | 01 Jan 2022 to 01 Apr 2022 | 10 | 9.3 | 4.6 | 16.8 | <10 | 5.6 | 1.7 | 9.5 |
| 28-day mortality | NHS ethnicity codes | Any other Black background | 23 Jan 2020 to 30 Jun 2020 | 95 | 366.6 | 330.5 | 405.6 | 50 | 205.8 | 179.1 | 235.3 |
| 28-day mortality | NHS ethnicity codes | Any other Black background | 01 Jul 2020 to 31 Dec 2020 | 45 | 86.5 | 69.7 | 106.2 | 40 | 100.2 | 82.0 | 121.2 |
| 28-day mortality | NHS ethnicity codes | Any other Black background | 01 Jan 2021 to 30 Jun 2021 | 55 | 102.5 | 84.0 | 123.8 | 30 | 37.9 | 27.2 | 51.5 |
| 28-day mortality | NHS ethnicity codes | Any other Black background | 01 Jul 2021 to 31 Dec 2021 | 40 | 47.3 | 35.2 | 62.3 | 20 | 16.3 | 9.7 | 25.7 |
| 28-day mortality | NHS ethnicity codes | Any other Black background | 01 Jan 2022 to 01 Apr 2022 | <10 | 16.1 | 3.2 | 28.9 | <10 | 4.8 | -1.8 | 11.4 |
| 28-day mortality | NHS ethnicity codes | Any other ethnic group | 23 Jan 2020 to 30 Jun 2020 | 215 | 256.6 | 222.2 | 290.9 | 135 | 160.7 | 133.8 | 187.6 |
| 28-day mortality | NHS ethnicity codes | Any other ethnic group | 01 Jul 2020 to 31 Dec 2020 | 125 | 73.8 | 60.9 | 86.7 | 80 | 41.9 | 30.6 | 56.1 |
| 28-day mortality | NHS ethnicity codes | Any other ethnic group | 01 Jan 2021 to 30 Jun 2021 | 145 | 97.3 | 81.4 | 113.2 | 100 | 51.3 | 41.3 | 61.2 |
| 28-day mortality | NHS ethnicity codes | Any other ethnic group | 01 Jul 2021 to 31 Dec 2021 | 70 | 30.5 | 21.1 | 42.9 | 45 | 19.2 | 12.0 | 29.3 |
| 28-day mortality | NHS ethnicity codes | Any other ethnic group | 01 Jan 2022 to 01 Apr 2022 | <10 | 3.3 | 0.1 | 6.5 | <10 | 6.3 | 2.2 | 10.4 |
| 28-day mortality | NHS ethnicity codes | Any other Mixed background | 23 Jan 2020 to 30 Jun 2020 | 20 | 125.3 | 104.8 | 148.7 | 20 | 93.7 | 76.2 | 114.2 |
| 28-day mortality | NHS ethnicity codes | Any other Mixed background | 01 Jul 2020 to 31 Dec 2020 | 25 | 69.6 | 54.7 | 87.5 | 25 | 45.6 | 33.7 | 60.3 |
| 28-day mortality | NHS ethnicity codes | Any other Mixed background | 01 Jan 2021 to 30 Jun 2021 | 40 | 116.8 | 97.0 | 139.4 | 20 | 40.9 | 29.7 | 54.9 |
| 28-day mortality | NHS ethnicity codes | Any other Mixed background | 01 Jul 2021 to 31 Dec 2021 | 25 | 32.9 | 23.0 | 45.6 | 20 | 30.6 | 21.1 | 42.9 |
| 28-day mortality | NHS ethnicity codes | Any other Mixed background | 01 Jan 2022 to 01 Apr 2022 | <10 | 3.9 | -3.7 | 11.5 | <10 | 7.9 | -3.1 | 19.0 |
| 28-day mortality | NHS ethnicity codes | Any other White background | 23 Jan 2020 to 30 Jun 2020 | 595 | 145.4 | 133.8 | 157.1 | 395 | 69.5 | 62.6 | 76.3 |
| 28-day mortality | NHS ethnicity codes | Any other White background | 01 Jul 2020 to 31 Dec 2020 | 770 | 62.7 | 58.2 | 67.1 | 565 | 31.7 | 29.0 | 34.3 |
| 28-day mortality | NHS ethnicity codes | Any other White background | 01 Jan 2021 to 30 Jun 2021 | 730 | 70.2 | 65.1 | 75.3 | 695 | 39.5 | 36.6 | 42.5 |
| 28-day mortality | NHS ethnicity codes | Any other White background | 01 Jul 2021 to 31 Dec 2021 | 440 | 20.2 | 18.3 | 22.1 | 350 | 13.6 | 12.1 | 15.0 |
| 28-day mortality | NHS ethnicity codes | Any other White background | 01 Jan 2022 to 01 Apr 2022 | 60 | 6.8 | 3.0 | 13.5 | 40 | 3.3 | 1.0 | 8.5 |
| 28-day mortality | NHS ethnicity codes | Arab | 23 Jan 2020 to 30 Jun 2020 | <10 | 98.0 | 2.0 | 194.1 | <10 | 375.4 | 97.3 | 653.5 |
| 28-day mortality | NHS ethnicity codes | Arab | 01 Jul 2020 to 31 Dec 2020 | <10 | 107.5 | 27.9 | 187.0 | <10 | 22.8 | -3.0 | 48.6 |
| 28-day mortality | NHS ethnicity codes | Arab | 01 Jan 2021 to 30 Jun 2021 | <10 | 46.5 | 12.1 | 81.0 | <10 | 69.6 | 8.6 | 130.6 |
| 28-day mortality | NHS ethnicity codes | Arab | 01 Jul 2021 to 31 Dec 2021 | <10 | 276.9 | - 106.9 | 660.7 | <10 | Inf | NA | Inf |
| 28-day mortality | NHS ethnicity codes | Arab | 01 Jan 2022 to 01 Apr 2022 | <10 | 25.8 | - 24.8 | 76.5 | <10 | 109.8 | - 105.4 | 324.9 |
| 28-day mortality | NHS ethnicity codes | Bangladeshi | 23 Jan 2020 to 30 Jun 2020 | 110 | 459.8 | 374.6 | 544.9 | 60 | 264.5 | 234.1 | 297.9 |
| 28-day mortality | NHS ethnicity codes | Bangladeshi | 01 Jul 2020 to 31 Dec 2020 | 185 | 133.1 | 113.9 | 152.4 | 120 | 68.9 | 56.5 | 81.4 |
| 28-day mortality | NHS ethnicity codes | Bangladeshi | 01 Jan 2021 to 30 Jun 2021 | 170 | 150.9 | 128.2 | 173.6 | 115 | 78.6 | 64.1 | 93.1 |
| 28-day mortality | NHS ethnicity codes | Bangladeshi | 01 Jul 2021 to 31 Dec 2021 | 65 | 51.6 | 38.9 | 67.1 | 50 | 35.6 | 25.3 | 48.8 |
| 28-day mortality | NHS ethnicity codes | Bangladeshi | 01 Jan 2022 to 01 Apr 2022 | <10 | 16.3 | 3.3 | 29.3 | <10 | 8.0 | -1.1 | 17.0 |
| 28-day mortality | NHS ethnicity codes | British | 23 Jan 2020 to 30 Jun 2020 | 12,190 | 186.8 | 183.5 | 190.1 | 9,355 | 106.5 | 104.3 | 108.6 |
| 28-day mortality | NHS ethnicity codes | British | 01 Jul 2020 to 31 Dec 2020 | 13,270 | 69.1 | 67.9 | 70.2 | 11,120 | 40.4 | 39.7 | 41.2 |
| 28-day mortality | NHS ethnicity codes | British | 01 Jan 2021 to 30 Jun 2021 | 11,180 | 79.4 | 77.9 | 80.9 | 10,320 | 47.1 | 46.2 | 48.0 |
| 28-day mortality | NHS ethnicity codes | British | 01 Jul 2021 to 31 Dec 2021 | 5,710 | 19.4 | 18.9 | 20.0 | 4,060 | 11.5 | 11.2 | 11.9 |
| 28-day mortality | NHS ethnicity codes | British | 01 Jan 2022 to 01 Apr 2022 | 785 | 5.8 | 5.4 | 6.2 | 650 | 3.4 | 3.1 | 3.6 |
| 28-day mortality | NHS ethnicity codes | Caribbean | 23 Jan 2020 to 30 Jun 2020 | 355 | 259.9 | 233.0 | 286.9 | 190 | 134.8 | 115.7 | 154.0 |
| 28-day mortality | NHS ethnicity codes | Caribbean | 01 Jul 2020 to 31 Dec 2020 | 195 | 88.2 | 75.8 | 100.7 | 125 | 45.8 | 37.8 | 53.9 |
| 28-day mortality | NHS ethnicity codes | Caribbean | 01 Jan 2021 to 30 Jun 2021 | 265 | 104.1 | 91.6 | 116.6 | 215 | 56.7 | 49.1 | 64.3 |
| 28-day mortality | NHS ethnicity codes | Caribbean | 01 Jul 2021 to 31 Dec 2021 | 170 | 40.6 | 34.4 | 46.7 | 135 | 23.6 | 19.6 | 27.5 |
| 28-day mortality | NHS ethnicity codes | Caribbean | 01 Jan 2022 to 01 Apr 2022 | 15 | 9.6 | 4.9 | 17.2 | 10 | 4.2 | 1.4 | 9.8 |
| 28-day mortality | NHS ethnicity codes | Chinese | 23 Jan 2020 to 30 Jun 2020 | 55 | 347.9 | 312.7 | 385.9 | 25 | 151.6 | 128.8 | 177.2 |
| 28-day mortality | NHS ethnicity codes | Chinese | 01 Jul 2020 to 31 Dec 2020 | 25 | 102.6 | 84.2 | 124.0 | 30 | 75.9 | 60.2 | 94.4 |
| 28-day mortality | NHS ethnicity codes | Chinese | 01 Jan 2021 to 30 Jun 2021 | 50 | 169.5 | 145.4 | 196.5 | 25 | 70.5 | 55.4 | 88.4 |
| 28-day mortality | NHS ethnicity codes | Chinese | 01 Jul 2021 to 31 Dec 2021 | 15 | 26.5 | 17.8 | 38.1 | 15 | 25.7 | 17.1 | 37.1 |
| 28-day mortality | NHS ethnicity codes | Chinese | 01 Jan 2022 to 01 Apr 2022 | <10 | 13.0 | 0.3 | 25.8 | <10 | 3.6 | -3.5 | 10.7 |
| 28-day mortality | NHS ethnicity codes | Gypsy or Irish Traveller | 23 Jan 2020 to 30 Jun 2020 | <10 | 782.9 | 240.4 | 1325.4 | <10 | 49.0 | - 47.0 | 145.1 |
| 28-day mortality | NHS ethnicity codes | Gypsy or Irish Traveller | 01 Jul 2020 to 31 Dec 2020 | <10 | 20.6 | -8.0 | 49.2 | <10 | 29.4 | - 28.2 | 87.0 |
| 28-day mortality | NHS ethnicity codes | Gypsy or Irish Traveller | 01 Jan 2021 to 30 Jun 2021 | - | - | NA | NA | <10 | 74.2 | 14.8 | 133.6 |
| 28-day mortality | NHS ethnicity codes | Gypsy or Irish Traveller | 01 Jul 2021 to 31 Dec 2021 | <10 | 2.5 | -2.4 | 7.4 | <10 | 1.2 | -1.2 | 3.6 |
| 28-day mortality | NHS ethnicity codes | Gypsy or Irish Traveller | 01 Jan 2022 to 01 Apr 2022 | <10 | 5.2 | -5.0 | 15.3 | - | - | NA | NA |
| 28-day mortality | NHS ethnicity codes | Indian | 23 Jan 2020 to 30 Jun 2020 | 510 | 235.9 | 215.5 | 256.4 | 295 | 152.4 | 135.1 | 169.7 |
| 28-day mortality | NHS ethnicity codes | Indian | 01 Jul 2020 to 31 Dec 2020 | 455 | 74.3 | 67.5 | 81.1 | 290 | 41.9 | 37.0 | 46.7 |
| 28-day mortality | NHS ethnicity codes | Indian | 01 Jan 2021 to 30 Jun 2021 | 470 | 83.3 | 75.8 | 90.9 | 320 | 53.1 | 47.3 | 58.9 |
| 28-day mortality | NHS ethnicity codes | Indian | 01 Jul 2021 to 31 Dec 2021 | 155 | 21.6 | 18.2 | 25.0 | 110 | 15.2 | 12.4 | 18.1 |
| 28-day mortality | NHS ethnicity codes | Indian | 01 Jan 2022 to 01 Apr 2022 | 25 | 10.4 | 5.4 | 18.3 | 15 | 4.6 | 1.7 | 10.4 |
| 28-day mortality | NHS ethnicity codes | Irish | 23 Jan 2020 to 30 Jun 2020 | 170 | 177.2 | 150.4 | 204.0 | 95 | 82.9 | 66.5 | 102.3 |
| 28-day mortality | NHS ethnicity codes | Irish | 01 Jul 2020 to 31 Dec 2020 | 170 | 70.7 | 60.1 | 81.4 | 140 | 39.4 | 32.9 | 46.0 |
| 28-day mortality | NHS ethnicity codes | Irish | 01 Jan 2021 to 30 Jun 2021 | 130 | 61.5 | 50.9 | 72.1 | 135 | 42.7 | 35.5 | 49.9 |
| 28-day mortality | NHS ethnicity codes | Irish | 01 Jul 2021 to 31 Dec 2021 | 70 | 18.3 | 11.3 | 28.2 | 55 | 12.6 | 7.0 | 21.1 |
| 28-day mortality | NHS ethnicity codes | Irish | 01 Jan 2022 to 01 Apr 2022 | <10 | 5.3 | 1.6 | 9.0 | <10 | 3.5 | 0.9 | 6.1 |
| 28-day mortality | NHS ethnicity codes | Pakistani | 23 Jan 2020 to 30 Jun 2020 | 285 | 248.2 | 219.3 | 277.0 | 180 | 163.9 | 139.9 | 187.9 |
| 28-day mortality | NHS ethnicity codes | Pakistani | 01 Jul 2020 to 31 Dec 2020 | 510 | 91.1 | 83.2 | 99.0 | 305 | 52.2 | 46.3 | 58.1 |
| 28-day mortality | NHS ethnicity codes | Pakistani | 01 Jan 2021 to 30 Jun 2021 | 385 | 89.5 | 80.6 | 98.5 | 245 | 53.2 | 46.5 | 59.8 |
| 28-day mortality | NHS ethnicity codes | Pakistani | 01 Jul 2021 to 31 Dec 2021 | 190 | 46.2 | 39.7 | 52.8 | 135 | 31.2 | 25.9 | 36.5 |
| 28-day mortality | NHS ethnicity codes | Pakistani | 01 Jan 2022 to 01 Apr 2022 | 20 | 15.0 | 8.7 | 24.1 | 10 | 8.3 | 4.0 | 15.6 |
| 28-day mortality | NHS ethnicity codes | Unknown/Not stated | 23 Jan 2020 to 30 Jun 2020 | 400 | 201.5 | 181.8 | 221.3 | 245 | 79.4 | 69.5 | 89.4 |
| 28-day mortality | NHS ethnicity codes | Unknown/Not stated | 01 Jul 2020 to 31 Dec 2020 | 305 | 52.4 | 46.5 | 58.3 | 270 | 30.5 | 26.9 | 34.2 |
| 28-day mortality | NHS ethnicity codes | Unknown/Not stated | 01 Jan 2021 to 30 Jun 2021 | 385 | 77.7 | 69.9 | 85.5 | 365 | 48.1 | 43.2 | 53.1 |
| 28-day mortality | NHS ethnicity codes | Unknown/Not stated | 01 Jul 2021 to 31 Dec 2021 | 195 | 19.6 | 16.8 | 22.3 | 160 | 13.1 | 11.1 | 15.1 |
| 28-day mortality | NHS ethnicity codes | Unknown/Not stated | 01 Jan 2022 to 01 Apr 2022 | 30 | 5.7 | 2.3 | 11.9 | 15 | 2.4 | 0.6 | 7.0 |
| 28-day mortality | NHS ethnicity codes | White and Asian | 23 Jan 2020 to 30 Jun 2020 | 25 | 289.7 | 257.7 | 324.5 | 15 | 184.6 | 159.4 | 212.7 |
| 28-day mortality | NHS ethnicity codes | White and Asian | 01 Jul 2020 to 31 Dec 2020 | 20 | 72.7 | 57.4 | 90.9 | 10 | 38.7 | 27.9 | 52.4 |
| 28-day mortality | NHS ethnicity codes | White and Asian | 01 Jan 2021 to 30 Jun 2021 | 20 | 101.6 | 83.3 | 122.9 | 15 | 53.8 | 40.8 | 69.7 |
| 28-day mortality | NHS ethnicity codes | White and Asian | 01 Jul 2021 to 31 Dec 2021 | 10 | 43.0 | 31.5 | 57.3 | <10 | 36.7 | 4.5 | 68.9 |
| 28-day mortality | NHS ethnicity codes | White and Asian | 01 Jan 2022 to 01 Apr 2022 | <10 | 5.3 | -5.1 | 15.6 | <10 | 19.6 | - 18.8 | 58.0 |
| 28-day mortality | NHS ethnicity codes | White and Black African | 23 Jan 2020 to 30 Jun 2020 | 20 | 224.2 | 196.2 | 255.0 | 15 | 144.7 | 122.6 | 169.8 |
| 28-day mortality | NHS ethnicity codes | White and Black African | 01 Jul 2020 to 31 Dec 2020 | 15 | 102.5 | 84.1 | 123.8 | 10 | 39.9 | 28.9 | 53.8 |
| 28-day mortality | NHS ethnicity codes | White and Black African | 01 Jan 2021 to 30 Jun 2021 | 15 | 92.1 | 74.7 | 112.4 | 10 | 129.9 | 109.0 | 153.8 |
| 28-day mortality | NHS ethnicity codes | White and Black African | 01 Jul 2021 to 31 Dec 2021 | <10 | 16.5 | 5.1 | 28.0 | <10 | 13.6 | 0.3 | 27.0 |
| 28-day mortality | NHS ethnicity codes | White and Black African | 01 Jan 2022 to 01 Apr 2022 | <10 | 10.2 | -3.9 | 24.3 | <10 | 0.9 | -0.9 | 2.7 |
| 28-day mortality | NHS ethnicity codes | White and Black Caribbean | 23 Jan 2020 to 30 Jun 2020 | 85 | 407.8 | 369.6 | 448.8 | 60 | 279.9 | 248.5 | 314.1 |
| 28-day mortality | NHS ethnicity codes | White and Black Caribbean | 01 Jul 2020 to 31 Dec 2020 | 45 | 142.3 | 120.3 | 167.1 | 15 | 41.9 | 30.6 | 56.1 |
| 28-day mortality | NHS ethnicity codes | White and Black Caribbean | 01 Jan 2021 to 30 Jun 2021 | 35 | 79.3 | 63.3 | 98.3 | 40 | 75.4 | 59.8 | 93.9 |
| 28-day mortality | NHS ethnicity codes | White and Black Caribbean | 01 Jul 2021 to 31 Dec 2021 | 25 | 32.6 | 22.8 | 45.3 | 35 | 45.4 | 33.6 | 60.2 |
| 28-day mortality | NHS ethnicity codes | White and Black Caribbean | 01 Jan 2022 to 01 Apr 2022 | <10 | 12.7 | 0.3 | 25.1 | <10 | 3.8 | -3.7 | 11.3 |
| 30-day CVD | High-level groups | Asian or Asian British | 23 Jan 2020 to 30 Jun 2020 | 515 | 106.9 | 97.7 | 116.1 | 255 | 55.9 | 49.0 | 62.7 |
| 30-day CVD | High-level groups | Asian or Asian British | 01 Jul 2020 to 31 Dec 2020 | 1,490 | 78.5 | 74.6 | 82.5 | 730 | 35.1 | 32.5 | 37.6 |
| 30-day CVD | High-level groups | Asian or Asian British | 01 Jan 2021 to 30 Jun 2021 | 1,395 | 85.1 | 80.7 | 89.6 | 645 | 38.6 | 35.6 | 41.6 |
| 30-day CVD | High-level groups | Asian or Asian British | 01 Jul 2021 to 31 Dec 2021 | 950 | 44.8 | 42.0 | 47.7 | 475 | 23.7 | 21.6 | 25.9 |
| 30-day CVD | High-level groups | Asian or Asian British | 01 Jan 2022 to 01 Apr 2022 | 315 | 30.4 | 27.0 | 33.7 | 180 | 17.2 | 14.7 | 19.8 |
| 30-day CVD | High-level groups | Black or Black British | 23 Jan 2020 to 30 Jun 2020 | 210 | 77.9 | 67.3 | 88.4 | 135 | 48.9 | 40.6 | 57.1 |
| 30-day CVD | High-level groups | Black or Black British | 01 Jul 2020 to 31 Dec 2020 | 255 | 52.6 | 46.2 | 59.1 | 195 | 35.0 | 30.1 | 39.9 |
| 30-day CVD | High-level groups | Black or Black British | 01 Jan 2021 to 30 Jun 2021 | 385 | 70.4 | 63.3 | 77.4 | 260 | 34.4 | 30.3 | 38.6 |
| 30-day CVD | High-level groups | Black or Black British | 01 Jul 2021 to 31 Dec 2021 | 360 | 39.6 | 35.5 | 43.7 | 275 | 22.9 | 20.2 | 25.6 |
| 30-day CVD | High-level groups | Black or Black British | 01 Jan 2022 to 01 Apr 2022 | 130 | 35.7 | 29.5 | 41.8 | 110 | 23.8 | 19.4 | 28.2 |
| 30-day CVD | High-level groups | Mixed | 23 Jan 2020 to 30 Jun 2020 | 55 | 98.4 | 80.3 | 119.3 | 50 | 81.2 | 65.0 | 100.4 |
| 30-day CVD | High-level groups | Mixed | 01 Jul 2020 to 31 Dec 2020 | 90 | 63.3 | 49.1 | 80.4 | 65 | 37.5 | 26.9 | 51.0 |
| 30-day CVD | High-level groups | Mixed | 01 Jan 2021 to 30 Jun 2021 | 90 | 61.5 | 47.6 | 78.4 | 55 | 33.9 | 23.9 | 46.9 |
| 30-day CVD | High-level groups | Mixed | 01 Jul 2021 to 31 Dec 2021 | 95 | 31.7 | 22.1 | 44.3 | 65 | 20.0 | 12.6 | 30.2 |
| 30-day CVD | High-level groups | Mixed | 01 Jan 2022 to 01 Apr 2022 | 35 | 31.3 | 21.7 | 43.7 | 20 | 12.9 | 7.2 | 21.5 |
| 30-day CVD | High-level groups | Other Ethnic Group | 23 Jan 2020 to 30 Jun 2020 | 90 | 114.2 | 94.7 | 136.7 | 45 | 56.8 | 43.4 | 73.1 |
| 30-day CVD | High-level groups | Other Ethnic Group | 01 Jul 2020 to 31 Dec 2020 | 165 | 66.6 | 56.5 | 76.7 | 80 | 34.6 | 24.4 | 47.6 |
| 30-day CVD | High-level groups | Other Ethnic Group | 01 Jan 2021 to 30 Jun 2021 | 170 | 83.6 | 71.1 | 96.2 | 75 | 32.3 | 22.6 | 45.0 |
| 30-day CVD | High-level groups | Other Ethnic Group | 01 Jul 2021 to 31 Dec 2021 | 150 | 46.1 | 38.7 | 53.4 | 70 | 20.1 | 12.7 | 30.4 |
| 30-day CVD | High-level groups | Other Ethnic Group | 01 Jan 2022 to 01 Apr 2022 | 45 | 34.8 | 24.6 | 47.9 | 40 | 25.9 | 17.3 | 37.4 |
| 30-day CVD | High-level groups | Unknown | 23 Jan 2020 to 30 Jun 2020 | 120 | 67.4 | 55.3 | 79.6 | 80 | 34.6 | 24.5 | 47.6 |
| 30-day CVD | High-level groups | Unknown | 01 Jul 2020 to 31 Dec 2020 | 255 | 40.8 | 35.8 | 45.8 | 175 | 19.9 | 17.0 | 22.9 |
| 30-day CVD | High-level groups | Unknown | 01 Jan 2021 to 30 Jun 2021 | 285 | 56.3 | 49.8 | 62.8 | 200 | 26.8 | 23.1 | 30.5 |
| 30-day CVD | High-level groups | Unknown | 01 Jul 2021 to 31 Dec 2021 | 305 | 24.4 | 21.7 | 27.1 | 190 | 13.1 | 11.3 | 15.0 |
| 30-day CVD | High-level groups | Unknown | 01 Jan 2022 to 01 Apr 2022 | 100 | 17.8 | 14.3 | 21.3 | 75 | 10.4 | 5.4 | 18.3 |
| 30-day CVD | High-level groups | White | 23 Jan 2020 to 30 Jun 2020 | 4,490 | 82.5 | 80.1 | 85.0 | 2,925 | 36.5 | 35.2 | 37.8 |
| 30-day CVD | High-level groups | White | 01 Jul 2020 to 31 Dec 2020 | 10,850 | 54.6 | 53.6 | 55.6 | 7,105 | 26.3 | 25.7 | 27.0 |
| 30-day CVD | High-level groups | White | 01 Jan 2021 to 30 Jun 2021 | 9,410 | 62.6 | 61.3 | 63.8 | 6,420 | 29.5 | 28.8 | 30.2 |
| 30-day CVD | High-level groups | White | 01 Jul 2021 to 31 Dec 2021 | 11,440 | 28.6 | 28.0 | 29.1 | 6,755 | 15.2 | 14.8 | 15.5 |
| 30-day CVD | High-level groups | White | 01 Jan 2022 to 01 Apr 2022 | 4,185 | 24.0 | 23.3 | 24.7 | 2,710 | 12.2 | 11.7 | 12.6 |
| 30-day CVD | NHS ethnicity codes | African | 23 Jan 2020 to 30 Jun 2020 | 80 | 72.6 | 57.3 | 90.8 | 55 | 50.9 | 38.3 | 66.4 |
| 30-day CVD | NHS ethnicity codes | African | 01 Jul 2020 to 31 Dec 2020 | 110 | 51.0 | 41.5 | 60.5 | 75 | 27.1 | 18.2 | 38.8 |
| 30-day CVD | NHS ethnicity codes | African | 01 Jan 2021 to 30 Jun 2021 | 145 | 66.0 | 55.2 | 76.7 | 90 | 26.6 | 17.9 | 38.2 |
| 30-day CVD | NHS ethnicity codes | African | 01 Jul 2021 to 31 Dec 2021 | 110 | 30.1 | 24.5 | 35.6 | 90 | 21.5 | 13.8 | 32.1 |
| 30-day CVD | NHS ethnicity codes | African | 01 Jan 2022 to 01 Apr 2022 | 50 | 40.5 | 29.4 | 54.4 | 40 | 18.9 | 11.8 | 29.0 |
| 30-day CVD | NHS ethnicity codes | Any other Asian background | 23 Jan 2020 to 30 Jun 2020 | 105 | 100.1 | 81.1 | 119.1 | 55 | 57.6 | 44.1 | 73.9 |
| 30-day CVD | NHS ethnicity codes | Any other Asian background | 01 Jul 2020 to 31 Dec 2020 | 230 | 76.6 | 66.6 | 86.5 | 105 | 29.9 | 24.1 | 35.7 |
| 30-day CVD | NHS ethnicity codes | Any other Asian background | 01 Jan 2021 to 30 Jun 2021 | 255 | 82.0 | 72.0 | 92.1 | 105 | 34.2 | 27.7 | 40.8 |
| 30-day CVD | NHS ethnicity codes | Any other Asian background | 01 Jul 2021 to 31 Dec 2021 | 160 | 37.5 | 31.6 | 43.3 | 85 | 21.4 | 13.7 | 31.9 |
| 30-day CVD | NHS ethnicity codes | Any other Asian background | 01 Jan 2022 to 01 Apr 2022 | 50 | 19.8 | 12.4 | 30.0 | 35 | 14.0 | 8.0 | 22.8 |
| 30-day CVD | NHS ethnicity codes | Any other Black background | 23 Jan 2020 to 30 Jun 2020 | 30 | 116.3 | 96.6 | 138.9 | 15 | 52.4 | 39.6 | 68.1 |
| 30-day CVD | NHS ethnicity codes | Any other Black background | 01 Jul 2020 to 31 Dec 2020 | 30 | 41.1 | 30.0 | 55.2 | 25 | 53.4 | 40.5 | 69.3 |
| 30-day CVD | NHS ethnicity codes | Any other Black background | 01 Jan 2021 to 30 Jun 2021 | 55 | 68.9 | 54.0 | 86.6 | 30 | 31.6 | 22.0 | 44.1 |
| 30-day CVD | NHS ethnicity codes | Any other Black background | 01 Jul 2021 to 31 Dec 2021 | 55 | 31.4 | 21.8 | 43.9 | 35 | 22.1 | 14.3 | 32.9 |
| 30-day CVD | NHS ethnicity codes | Any other Black background | 01 Jan 2022 to 01 Apr 2022 | 20 | 32.4 | 22.6 | 45.0 | 10 | 31.5 | 21.9 | 44.0 |
| 30-day CVD | NHS ethnicity codes | Any other ethnic group | 23 Jan 2020 to 30 Jun 2020 | 80 | 109.7 | 90.6 | 131.7 | 40 | 52.3 | 39.5 | 68.0 |
| 30-day CVD | NHS ethnicity codes | Any other ethnic group | 01 Jul 2020 to 31 Dec 2020 | 150 | 65.4 | 54.9 | 75.9 | 80 | 34.8 | 24.6 | 47.8 |
| 30-day CVD | NHS ethnicity codes | Any other ethnic group | 01 Jan 2021 to 30 Jun 2021 | 155 | 83.3 | 70.2 | 96.4 | 70 | 31.4 | 21.8 | 43.9 |
| 30-day CVD | NHS ethnicity codes | Any other ethnic group | 01 Jul 2021 to 31 Dec 2021 | 140 | 46.3 | 38.6 | 54.0 | 65 | 19.7 | 12.3 | 29.9 |
| 30-day CVD | NHS ethnicity codes | Any other ethnic group | 01 Jan 2022 to 01 Apr 2022 | 40 | 32.2 | 22.4 | 44.8 | 35 | 23.3 | 15.2 | 34.2 |
| 30-day CVD | NHS ethnicity codes | Any other Mixed background | 23 Jan 2020 to 30 Jun 2020 | 15 | 109.3 | 90.2 | 131.3 | 10 | 51.5 | 38.8 | 67.0 |
| 30-day CVD | NHS ethnicity codes | Any other Mixed background | 01 Jul 2020 to 31 Dec 2020 | 35 | 65.6 | 51.2 | 83.0 | 20 | 36.1 | 25.7 | 49.4 |
| 30-day CVD | NHS ethnicity codes | Any other Mixed background | 01 Jan 2021 to 30 Jun 2021 | 35 | 82.2 | 65.9 | 101.5 | 15 | 24.3 | 16.0 | 35.5 |
| 30-day CVD | NHS ethnicity codes | Any other Mixed background | 01 Jul 2021 to 31 Dec 2021 | 30 | 29.8 | 20.5 | 42.0 | 25 | 15.3 | 9.0 | 24.4 |
| 30-day CVD | NHS ethnicity codes | Any other Mixed background | 01 Jan 2022 to 01 Apr 2022 | <10 | 16.4 | 5.0 | 27.8 | 10 | 21.9 | 14.1 | 32.6 |
| 30-day CVD | NHS ethnicity codes | Any other White background | 23 Jan 2020 to 30 Jun 2020 | 285 | 85.7 | 75.8 | 95.6 | 165 | 33.1 | 28.1 | 38.2 |
| 30-day CVD | NHS ethnicity codes | Any other White background | 01 Jul 2020 to 31 Dec 2020 | 740 | 59.1 | 54.9 | 63.4 | 460 | 27.7 | 25.2 | 30.2 |
| 30-day CVD | NHS ethnicity codes | Any other White background | 01 Jan 2021 to 30 Jun 2021 | 760 | 68.2 | 63.3 | 73.0 | 475 | 29.0 | 26.4 | 31.6 |
| 30-day CVD | NHS ethnicity codes | Any other White background | 01 Jul 2021 to 31 Dec 2021 | 835 | 30.7 | 28.6 | 32.8 | 535 | 17.4 | 15.9 | 18.9 |
| 30-day CVD | NHS ethnicity codes | Any other White background | 01 Jan 2022 to 01 Apr 2022 | 340 | 29.3 | 26.2 | 32.4 | 220 | 15.3 | 13.2 | 17.3 |
| 30-day CVD | NHS ethnicity codes | Arab | 23 Jan 2020 to 30 Jun 2020 | <10 | 198.0 | 60.8 | 335.2 | <10 | 938.4 | 18.8 | 1,857.9 |
| 30-day CVD | NHS ethnicity codes | Arab | 01 Jul 2020 to 31 Dec 2020 | 20 | 67.0 | 52.3 | 84.5 | <10 | 34.3 | 0.7 | 68.0 |
| 30-day CVD | NHS ethnicity codes | Arab | 01 Jan 2021 to 30 Jun 2021 | 15 | 86.4 | 69.6 | 106.2 | <10 | 50.2 | 10.0 | 90.4 |
| 30-day CVD | NHS ethnicity codes | Arab | 01 Jul 2021 to 31 Dec 2021 | 15 | 39.6 | 28.6 | 53.4 | <10 | 40.8 | 14.2 | 67.5 |
| 30-day CVD | NHS ethnicity codes | Arab | 01 Jan 2022 to 01 Apr 2022 | <10 | 100.4 | 20.1 | 180.7 | <10 | 2,761.9 | - 363.5 | 5,887.2 |
| 30-day CVD | NHS ethnicity codes | Bangladeshi | 23 Jan 2020 to 30 Jun 2020 | 35 | 170.4 | 146.2 | 197.4 | 25 | 98.9 | 80.8 | 119.8 |
| 30-day CVD | NHS ethnicity codes | Bangladeshi | 01 Jul 2020 to 31 Dec 2020 | 185 | 103.0 | 88.3 | 117.8 | 80 | 35.8 | 25.5 | 49.1 |
| 30-day CVD | NHS ethnicity codes | Bangladeshi | 01 Jan 2021 to 30 Jun 2021 | 170 | 110.1 | 93.5 | 126.8 | 80 | 47.1 | 35.0 | 62.0 |
| 30-day CVD | NHS ethnicity codes | Bangladeshi | 01 Jul 2021 to 31 Dec 2021 | 100 | 65.9 | 53.1 | 78.8 | 40 | 25.0 | 16.6 | 36.3 |
| 30-day CVD | NHS ethnicity codes | Bangladeshi | 01 Jan 2022 to 01 Apr 2022 | 30 | 44.9 | 33.1 | 59.5 | 20 | 42.5 | 31.1 | 56.7 |
| 30-day CVD | NHS ethnicity codes | British | 23 Jan 2020 to 30 Jun 2020 | 4,140 | 82.2 | 79.7 | 84.7 | 2,730 | 36.8 | 35.5 | 38.2 |
| 30-day CVD | NHS ethnicity codes | British | 01 Jul 2020 to 31 Dec 2020 | 9,965 | 54.1 | 53.1 | 55.2 | 6,555 | 26.2 | 25.6 | 26.9 |
| 30-day CVD | NHS ethnicity codes | British | 01 Jan 2021 to 30 Jun 2021 | 8,525 | 62.0 | 60.7 | 63.3 | 5,865 | 29.6 | 28.8 | 30.3 |
| 30-day CVD | NHS ethnicity codes | British | 01 Jul 2021 to 31 Dec 2021 | 10,470 | 28.4 | 27.8 | 28.9 | 6,135 | 15.0 | 14.6 | 15.4 |
| 30-day CVD | NHS ethnicity codes | British | 01 Jan 2022 to 01 Apr 2022 | 3,795 | 23.6 | 22.9 | 24.4 | 2,460 | 11.9 | 11.5 | 12.4 |
| 30-day CVD | NHS ethnicity codes | Caribbean | 23 Jan 2020 to 30 Jun 2020 | 100 | 94.8 | 76.3 | 113.3 | 70 | 49.8 | 37.4 | 65.1 |
| 30-day CVD | NHS ethnicity codes | Caribbean | 01 Jul 2020 to 31 Dec 2020 | 115 | 59.8 | 48.9 | 70.6 | 95 | 40.3 | 29.2 | 54.2 |
| 30-day CVD | NHS ethnicity codes | Caribbean | 01 Jan 2021 to 30 Jun 2021 | 185 | 77.5 | 66.4 | 88.6 | 145 | 40.5 | 33.9 | 47.1 |
| 30-day CVD | NHS ethnicity codes | Caribbean | 01 Jul 2021 to 31 Dec 2021 | 195 | 45.7 | 39.3 | 52.2 | 150 | 23.7 | 19.9 | 27.5 |
| 30-day CVD | NHS ethnicity codes | Caribbean | 01 Jan 2022 to 01 Apr 2022 | 60 | 41.0 | 29.8 | 55.0 | 65 | 27.1 | 18.3 | 38.8 |
| 30-day CVD | NHS ethnicity codes | Chinese | 23 Jan 2020 to 30 Jun 2020 | 15 | 133.6 | 112.4 | 157.8 | <10 | 28.4 | 0.6 | 56.1 |
| 30-day CVD | NHS ethnicity codes | Chinese | 01 Jul 2020 to 31 Dec 2020 | 25 | 81.2 | 64.9 | 100.3 | 15 | 41.2 | 30.0 | 55.2 |
| 30-day CVD | NHS ethnicity codes | Chinese | 01 Jan 2021 to 30 Jun 2021 | 30 | 85.4 | 68.7 | 105.0 | 10 | 31.9 | 22.2 | 44.5 |
| 30-day CVD | NHS ethnicity codes | Chinese | 01 Jul 2021 to 31 Dec 2021 | 25 | 34.8 | 24.6 | 47.8 | 15 | 14.7 | 8.6 | 23.8 |
| 30-day CVD | NHS ethnicity codes | Chinese | 01 Jan 2022 to 01 Apr 2022 | <10 | 11.9 | 0.2 | 23.5 | <10 | 6.1 | -2.3 | 14.4 |
| 30-day CVD | NHS ethnicity codes | Gypsy or Irish Traveller | 23 Jan 2020 to 30 Jun 2020 | <10 | 119.2 | - 15.7 | 254.0 | <10 | 50.4 | - 19.4 | 120.2 |
| 30-day CVD | NHS ethnicity codes | Gypsy or Irish Traveller | 01 Jul 2020 to 31 Dec 2020 | <10 | 60.5 | 1.2 | 119.9 | <10 | 363.3 | 7.3 | 719.3 |
| 30-day CVD | NHS ethnicity codes | Gypsy or Irish Traveller | 01 Jan 2021 to 30 Jun 2021 | <10 | 108.7 | 21.7 | 195.6 | <10 | 122.4 | 2.4 | 242.4 |
| 30-day CVD | NHS ethnicity codes | Gypsy or Irish Traveller | 01 Jul 2021 to 31 Dec 2021 | <10 | 7.0 | -2.7 | 16.6 | <10 | 9.3 | 1.1 | 17.5 |
| 30-day CVD | NHS ethnicity codes | Gypsy or Irish Traveller | 01 Jan 2022 to 01 Apr 2022 | <10 | 14.7 | -5.7 | 35.0 | <10 | 6.9 | -6.7 | 20.6 |
| 30-day CVD | NHS ethnicity codes | Indian | 23 Jan 2020 to 30 Jun 2020 | 205 | 96.0 | 82.9 | 109.2 | 95 | 48.2 | 36.0 | 63.3 |
| 30-day CVD | NHS ethnicity codes | Indian | 01 Jul 2020 to 31 Dec 2020 | 460 | 63.8 | 58.0 | 69.7 | 250 | 31.6 | 27.7 | 35.5 |
| 30-day CVD | NHS ethnicity codes | Indian | 01 Jan 2021 to 30 Jun 2021 | 450 | 69.6 | 63.2 | 76.1 | 230 | 36.8 | 32.1 | 41.6 |
| 30-day CVD | NHS ethnicity codes | Indian | 01 Jul 2021 to 31 Dec 2021 | 335 | 36.1 | 32.3 | 40.0 | 155 | 18.3 | 15.5 | 21.2 |
| 30-day CVD | NHS ethnicity codes | Indian | 01 Jan 2022 to 01 Apr 2022 | 105 | 24.6 | 19.9 | 29.2 | 65 | 15.6 | 9.2 | 24.9 |
| 30-day CVD | NHS ethnicity codes | Irish | 23 Jan 2020 to 30 Jun 2020 | 60 | 69.6 | 54.7 | 87.5 | 30 | 31.4 | 21.8 | 43.8 |
| 30-day CVD | NHS ethnicity codes | Irish | 01 Jul 2020 to 31 Dec 2020 | 140 | 63.3 | 52.9 | 73.7 | 85 | 27.8 | 18.8 | 39.6 |
| 30-day CVD | NHS ethnicity codes | Irish | 01 Jan 2021 to 30 Jun 2021 | 120 | 63.4 | 52.1 | 74.7 | 80 | 30.2 | 20.8 | 42.5 |
| 30-day CVD | NHS ethnicity codes | Irish | 01 Jul 2021 to 31 Dec 2021 | 135 | 31.8 | 26.4 | 37.1 | 80 | 17.3 | 10.5 | 27.0 |
| 30-day CVD | NHS ethnicity codes | Irish | 01 Jan 2022 to 01 Apr 2022 | 50 | 28.3 | 19.3 | 40.2 | 35 | 13.9 | 8.0 | 22.8 |
| 30-day CVD | NHS ethnicity codes | Pakistani | 23 Jan 2020 to 30 Jun 2020 | 155 | 129.4 | 108.9 | 149.9 | 75 | 64.2 | 49.9 | 81.4 |
| 30-day CVD | NHS ethnicity codes | Pakistani | 01 Jul 2020 to 31 Dec 2020 | 590 | 91.5 | 84.1 | 98.9 | 280 | 41.0 | 36.2 | 45.9 |
| 30-day CVD | NHS ethnicity codes | Pakistani | 01 Jan 2021 to 30 Jun 2021 | 490 | 102.1 | 93.1 | 111.1 | 220 | 42.1 | 36.5 | 47.6 |
| 30-day CVD | NHS ethnicity codes | Pakistani | 01 Jul 2021 to 31 Dec 2021 | 330 | 65.5 | 58.5 | 72.6 | 175 | 38.3 | 32.6 | 44.0 |
| 30-day CVD | NHS ethnicity codes | Pakistani | 01 Jan 2022 to 01 Apr 2022 | 125 | 55.2 | 45.5 | 65.0 | 55 | 21.1 | 13.5 | 31.6 |
| 30-day CVD | NHS ethnicity codes | Unknown/Not stated | 23 Jan 2020 to 30 Jun 2020 | 120 | 67.4 | 55.3 | 79.6 | 80 | 34.6 | 24.5 | 47.6 |
| 30-day CVD | NHS ethnicity codes | Unknown/Not stated | 01 Jul 2020 to 31 Dec 2020 | 255 | 40.8 | 35.8 | 45.8 | 175 | 19.9 | 17.0 | 22.9 |
| 30-day CVD | NHS ethnicity codes | Unknown/Not stated | 01 Jan 2021 to 30 Jun 2021 | 285 | 56.3 | 49.8 | 62.8 | 200 | 26.8 | 23.1 | 30.5 |
| 30-day CVD | NHS ethnicity codes | Unknown/Not stated | 01 Jul 2021 to 31 Dec 2021 | 305 | 24.4 | 21.7 | 27.1 | 190 | 13.1 | 11.3 | 15.0 |
| 30-day CVD | NHS ethnicity codes | Unknown/Not stated | 01 Jan 2022 to 01 Apr 2022 | 100 | 17.8 | 14.3 | 21.3 | 75 | 10.4 | 5.4 | 18.3 |
| 30-day CVD | NHS ethnicity codes | White and Asian | 23 Jan 2020 to 30 Jun 2020 | 10 | 129.3 | 108.5 | 153.1 | <10 | 53.7 | 10.7 | 96.8 |
| 30-day CVD | NHS ethnicity codes | White and Asian | 01 Jul 2020 to 31 Dec 2020 | 25 | 76.2 | 60.5 | 94.8 | 15 | 37.4 | 26.8 | 50.9 |
| 30-day CVD | NHS ethnicity codes | White and Asian | 01 Jan 2021 to 30 Jun 2021 | 15 | 58.3 | 44.8 | 74.8 | 10 | 42.2 | 30.9 | 56.5 |
| 30-day CVD | NHS ethnicity codes | White and Asian | 01 Jul 2021 to 31 Dec 2021 | 20 | 30.0 | 20.7 | 42.2 | <10 | 10.4 | 2.7 | 18.0 |
| 30-day CVD | NHS ethnicity codes | White and Asian | 01 Jan 2022 to 01 Apr 2022 | 15 | 58.5 | 44.9 | 75.0 | <10 | 554.7 | - 73.0 | 1,182.4 |
| 30-day CVD | NHS ethnicity codes | White and Black African | 23 Jan 2020 to 30 Jun 2020 | 10 | 112.2 | 92.8 | 134.4 | 10 | 99.6 | 81.4 | 120.6 |
| 30-day CVD | NHS ethnicity codes | White and Black African | 01 Jul 2020 to 31 Dec 2020 | 10 | 46.2 | 34.3 | 61.0 | <10 | 9.3 | 1.9 | 16.7 |
| 30-day CVD | NHS ethnicity codes | White and Black African | 01 Jan 2021 to 30 Jun 2021 | 15 | 60.3 | 46.5 | 77.0 | 15 | 56.7 | 43.3 | 72.9 |
| 30-day CVD | NHS ethnicity codes | White and Black African | 01 Jul 2021 to 31 Dec 2021 | <10 | 14.6 | 4.5 | 24.6 | <10 | 25.1 | 5.0 | 45.2 |
| 30-day CVD | NHS ethnicity codes | White and Black African | 01 Jan 2022 to 01 Apr 2022 | <10 | 96.8 | 25.1 | 168.6 | <10 | 0.5 | - 0.4 | 1.4 |
| 30-day CVD | NHS ethnicity codes | White and Black Caribbean | 23 Jan 2020 to 30 Jun 2020 | 15 | 107.3 | 88.4 | 129.1 | 25 | 111.1 | 91.9 | 133.2 |
| 30-day CVD | NHS ethnicity codes | White and Black Caribbean | 01 Jul 2020 to 31 Dec 2020 | 20 | 76.2 | 60.5 | 94.8 | 25 | 68.9 | 54.0 | 86.6 |
| 30-day CVD | NHS ethnicity codes | White and Black Caribbean | 01 Jan 2021 to 30 Jun 2021 | 25 | 44.8 | 33.1 | 59.4 | 20 | 30.3 | 20.9 | 42.6 |
| 30-day CVD | NHS ethnicity codes | White and Black Caribbean | 01 Jul 2021 to 31 Dec 2021 | 40 | 42.3 | 31.0 | 56.6 | 25 | 29.1 | 19.9 | 41.2 |
| 30-day CVD | NHS ethnicity codes | White and Black Caribbean | 01 Jan 2022 to 01 Apr 2022 | <10 | 40.4 | 12.4 | 68.4 | <10 | 9.0 | 2.3 | 15.7 |

Supplementary Table 15. Adjusted hazard ratios of 28-day mortality from a) men and b) women diagnosed with COVID-19 with diverse ethnic background by the period of COVID-19 diagnosis in England, using White British women as reference group. Period of recorded COVID-19 diagnosis is determined by date of COVID-19 diagnosis. Ethnic classification used: NHS ethnicity codes.

#### a) Men estimates

| **Time of COVID-19 diagnosis** | **23 Jan 2020 to 30 Jun 2020** | | | **01 Jul 2020 to 31 Dec 2020** | | | **01 Jan 2021 to 30 Jun 2021** | | | **01 Jul 2021 to 31 Dec 2021** | | | **01 Jan 2022 to 01 Apr 2022** | | |
| --- | --- | --- | --- | --- | --- | --- | --- | --- | --- | --- | --- | --- | --- | --- | --- |
| **Variables** | **HR** | **Low 95% CI** | **High 95% CI** | **HR** | **Low 95% CI** | **High 95% CI** | **HR** | **Low 95% CI** | **High 95% CI** | **HR** | **Low 95% CI** | **High 95% CI** | **HR** | **Low 95% CI** | **High 95% CI** |
| Indian | 1.17 | 1.06 | 1.28 | 1.11 | 1.01 | 1.23 | 1.09 | 0.99 | 1.20 | 0.96 | 0.82 | 1.13 | 1.24 | 0.82 | 1.86 |
| Pakistani | 1.19 | 1.06 | 1.34 | 1.31 | 1.19 | 1.43 | 1.15 | 1.04 | 1.28 | 1.33 | 1.15 | 1.55 | 1.55 | 1.00 | 2.39 |
| Bangladeshi | 1.75 | 1.45 | 2.11 | 1.57 | 1.35 | 1.82 | 1.55 | 1.33 | 1.81 | 1.68 | 1.31 | 2.16 | 1.25 | 0.55 | 2.83 |
| Chinese | 1.64 | 1.26 | 2.15 | 1.35 | 0.91 | 1.99 | 2.16 | 1.63 | 2.86 | 1.19 | 0.73 | 1.95 | 2.07 | 0.77 | 5.56 |
| Any other Asian background | 0.84 | 0.73 | 0.98 | 1.21 | 1.05 | 1.40 | 1.18 | 1.03 | 1.35 | 1.18 | 0.94 | 1.47 | 1.43 | 0.80 | 2.55 |
| African | 1.11 | 0.98 | 1.26 | 1.12 | 0.93 | 1.35 | 1.40 | 1.21 | 1.61 | 0.86 | 0.67 | 1.12 | 0.77 | 0.38 | 1.57 |
| Caribbean | 0.87 | 0.78 | 0.97 | 1.09 | 0.94 | 1.26 | 1.03 | 0.91 | 1.17 | 0.84 | 0.72 | 0.99 | 0.49 | 0.28 | 0.87 |
| Any other Black background | 1.40 | 1.14 | 1.72 | 1.41 | 1.06 | 1.88 | 1.10 | 0.84 | 1.44 | 1.26 | 0.92 | 1.71 | 0.90 | 0.40 | 2.04 |
| White and Black Caribbean | 1.43 | 1.15 | 1.77 | 1.80 | 1.35 | 2.41 | 0.95 | 0.68 | 1.33 | 1.12 | 0.76 | 1.63 | 1.06 | 0.39 | 2.85 |
| White and Black African | 0.94 | 0.61 | 1.45 | 1.18 | 0.70 | 1.99 | 0.87 | 0.52 | 1.44 | 1.20 | 0.60 | 2.40 | 1.51 | 0.37 | 6.10 |
| White and Asian | 1.66 | 1.13 | 2.42 | 1.17 | 0.73 | 1.85 | 1.44 | 0.94 | 2.22 | 1.32 | 0.71 | 2.47 | 1.19 | 0.17 | 8.50 |
| Any other Mixed background | 0.71 | 0.46 | 1.07 | 0.92 | 0.62 | 1.35 | 1.32 | 0.97 | 1.80 | 1.55 | 1.04 | 2.33 | 0.53 | 0.07 | 3.80 |
| Arab | 0.35 | 0.13 | 0.93 | 0.63 | 0.30 | 1.32 | 0.61 | 0.29 | 1.28 | 0.37 | 0.09 | 1.49 | 1.37 | 0.19 | 9.81 |
| Any Other Ethnic Group | 1.20 | 1.05 | 1.38 | 1.09 | 0.91 | 1.30 | 1.15 | 0.97 | 1.35 | 1.31 | 1.03 | 1.66 | 0.61 | 0.23 | 1.63 |
| Irish | 0.79 | 0.67 | 0.92 | 1.00 | 0.86 | 1.17 | 0.79 | 0.67 | 0.94 | 1.00 | 0.79 | 1.26 | 0.80 | 0.40 | 1.61 |
| Gypsy or Irish Traveller | 1.17 | 0.59 | 2.35 | 0.46 | 0.11 | 1.83 | 0.00 | 0.00 | 2.05E +113 | 0.40 | 0.06 | 2.82 | 2.04 | 0.29 | 14.59 |
| Any other White background | 0.76 | 0.70 | 0.83 | 0.91 | 0.84 | 0.98 | 0.90 | 0.83 | 0.97 | 0.99 | 0.90 | 1.09 | 0.99 | 0.75 | 1.29 |
| Unknown/Not stated | 1.03 | 0.93 | 1.14 | 0.75 | 0.67 | 0.84 | 0.98 | 0.89 | 1.09 | 0.95 | 0.83 | 1.10 | 1.05 | 0.73 | 1.51 |
| Age | 1.22 | 1.20 | 1.24 | 1.33 | 1.31 | 1.35 | 1.27 | 1.25 | 1.29 | 1.16 | 1.15 | 1.18 | 1.20 | 1.14 | 1.26 |
| I(age^2) | 1.00 | 1.00 | 1.00 | 1.00 | 1.00 | 1.00 | 1.00 | 1.00 | 1.00 | 1.00 | 1.00 | 1.00 | 1.00 | 1.00 | 1.00 |
| Vaccination (yes) | NA | NA | NA | 0.75 | 0.64 | 0.88 | 0.77 | 0.74 | 0.80 | 0.16 | 0.15 | 0.17 | 0.14 | 0.12 | 0.17 |
| IMD quintile: IMD 2 | 0.96 | 0.91 | 1.00 | 0.92 | 0.88 | 0.97 | 0.90 | 0.86 | 0.95 | 0.82 | 0.76 | 0.87 | 0.89 | 0.74 | 1.06 |
| IMD quintile: IMD 3 | 0.90 | 0.86 | 0.95 | 0.86 | 0.82 | 0.90 | 0.85 | 0.81 | 0.89 | 0.69 | 0.65 | 0.74 | 0.71 | 0.58 | 0.86 |
| IMD quintile: IMD 4 | 0.91 | 0.86 | 0.95 | 0.80 | 0.76 | 0.84 | 0.79 | 0.74 | 0.83 | 0.65 | 0.60 | 0.70 | 0.65 | 0.53 | 0.80 |
| IMD quintile: IMD 5 (less deprived) | 0.83 | 0.79 | 0.88 | 0.77 | 0.73 | 0.81 | 0.78 | 0.73 | 0.82 | 0.60 | 0.55 | 0.65 | 0.73 | 0.60 | 0.90 |
| IMD quintile: IMD Unknown | 0.89 | 0.52 | 1.54 | 0.99 | 0.49 | 1.98 | 1.13 | 0.59 | 2.19 | 0.51 | 0.13 | 2.05 | 0.00 | 0.00 | Inf |
| LSOA region: East of England | 1.11 | 1.02 | 1.20 | 1.03 | 0.96 | 1.11 | 1.04 | 0.96 | 1.12 | 0.96 | 0.86 | 1.07 | 0.79 | 0.58 | 1.09 |
| LSOA region: London | 0.96 | 0.89 | 1.03 | 0.92 | 0.85 | 0.99 | 0.87 | 0.81 | 0.94 | 0.79 | 0.71 | 0.88 | 0.92 | 0.69 | 1.21 |
| LSOA region: North East | 0.94 | 0.85 | 1.04 | 0.92 | 0.84 | 1.00 | 0.82 | 0.74 | 0.92 | 0.89 | 0.78 | 1.00 | 1.11 | 0.82 | 1.50 |
| LSOA region: North West | 1.00 | 0.93 | 1.08 | 0.96 | 0.90 | 1.02 | 0.93 | 0.86 | 1.00 | 0.83 | 0.76 | 0.92 | 0.73 | 0.56 | 0.95 |
| LSOA region: South East | 0.97 | 0.90 | 1.05 | 1.01 | 0.95 | 1.08 | 1.00 | 0.93 | 1.08 | 0.83 | 0.75 | 0.92 | 0.84 | 0.63 | 1.11 |
| LSOA region: South West | 0.86 | 0.78 | 0.95 | 0.88 | 0.80 | 0.96 | 0.86 | 0.78 | 0.95 | 0.85 | 0.76 | 0.95 | 0.87 | 0.63 | 1.19 |
| LSOA region: Unknown | 0.95 | 0.88 | 1.02 | 0.92 | 0.86 | 0.98 | 0.92 | 0.85 | 0.99 | 0.88 | 0.79 | 0.98 | 0.62 | 0.45 | 0.84 |
| LSOA region: West Midlands | 1.09 | 1.00 | 1.18 | 0.99 | 0.92 | 1.06 | 0.92 | 0.84 | 1.00 | 0.84 | 0.76 | 0.93 | 0.94 | 0.71 | 1.23 |
| LSOA region: Yorkshire and The Humber | 1.00 | 0.92 | 1.08 | 0.90 | 0.83 | 0.97 | 0.94 | 0.87 | 1.02 | 0.84 | 0.76 | 0.94 | 0.82 | 0.61 | 1.10 |
| Atrial Fibrillation (yes) | 0.96 | 0.92 | 0.99 | 1.12 | 1.08 | 1.16 | 1.12 | 1.08 | 1.17 | 1.29 | 1.22 | 1.37 | 1.32 | 1.14 | 1.54 |
| Obesity (yes) | 1.04 | 0.99 | 1.10 | 1.05 | 1.00 | 1.11 | 1.09 | 1.04 | 1.15 | 1.18 | 1.11 | 1.27 | 0.95 | 0.77 | 1.17 |
| Chronic Kidney Disease (yes) | 1.15 | 1.11 | 1.19 | 1.46 | 1.41 | 1.51 | 1.40 | 1.35 | 1.45 | 1.81 | 1.72 | 1.91 | 1.61 | 1.40 | 1.86 |
| Diabetes (yes) | 1.14 | 1.10 | 1.18 | 1.33 | 1.28 | 1.37 | 1.29 | 1.25 | 1.34 | 1.58 | 1.50 | 1.66 | 1.39 | 1.21 | 1.60 |
| Chronic mental health disorders (yes) | 1.08 | 1.00 | 1.17 | 1.34 | 1.24 | 1.45 | 1.20 | 1.11 | 1.31 | 1.24 | 1.09 | 1.41 | 1.29 | 0.92 | 1.81 |
| Use of CVD prevention medication (yes) | 1.06 | 1.02 | 1.10 | 1.06 | 1.02 | 1.11 | 1.14 | 1.10 | 1.19 | 1.12 | 1.06 | 1.19 | 0.94 | 0.80 | 1.09 |
| Rheumatoid Arthritis (yes) | 1.21 | 1.09 | 1.34 | 1.28 | 1.15 | 1.42 | 1.29 | 1.15 | 1.44 | 2.05 | 1.80 | 2.33 | 1.42 | 0.95 | 2.14 |
| Antipsychotic (yes) | 1.19 | 1.07 | 1.32 | 1.18 | 1.05 | 1.33 | 1.36 | 1.21 | 1.52 | 1.72 | 1.40 | 2.13 | 1.17 | 0.72 | 1.92 |
| Cancer (yes) | 1.08 | 1.04 | 1.12 | 1.29 | 1.25 | 1.34 | 1.31 | 1.25 | 1.36 | 1.60 | 1.52 | 1.69 | 1.59 | 1.38 | 1.83 |
| Chronic obstructive pulmonary disease (yes) | 1.10 | 1.06 | 1.15 | 1.52 | 1.46 | 1.58 | 1.49 | 1.43 | 1.56 | 1.88 | 1.77 | 1.99 | 1.87 | 1.60 | 2.19 |
| Dementia (yes) | 1.36 | 1.31 | 1.41 | 1.46 | 1.41 | 1.52 | 1.39 | 1.33 | 1.45 | 1.64 | 1.53 | 1.77 | 1.68 | 1.43 | 1.98 |
| Hypertension (yes) | 0.95 | 0.92 | 0.98 | 0.98 | 0.95 | 1.01 | 0.96 | 0.93 | 1.00 | 1.04 | 0.99 | 1.09 | 0.97 | 0.84 | 1.11 |
| **Reference group**s: White British for ethnicity; IMD 1 (most deprived) for IMD quintile; East Midlands for English region.  **Abbreviations**: CI, confidence interval; CVD, cardiovascular disease; IMD, index of multiple deprivation; I(age^2), main effect and the second order interaction of age. | | | | | | | | | | | | | | | |

#### b) Women estimates

| **Time of COVID-19 diagnosis** | **23 Jan 2020 to 30 Jun 2020** | | | **01 Jul 2020 to 31 Dec 2020** | | | **01 Jan 2021 to 30 Jun 2021** | | | **01 Jul 2021 to 31 Dec 2021** | | | **01 Jan 2022 to 01 Apr 2022** | | |
| --- | --- | --- | --- | --- | --- | --- | --- | --- | --- | --- | --- | --- | --- | --- | --- |
| **Variables** | **HR** | **Low 95% CI** | **High 95% CI** | **HR** | **Low 95% CI** | **High 95% CI** | **HR** | **Low 95% CI** | **High 95% CI** | **HR** | **Low 95% CI** | **High 95% CI** | **HR** | **Low 95% CI** | **High 95% CI** |
| Indian | 1.36 | 1.21 | 1.53 | 1.09 | 0.96 | 1.22 | 1.12 | 1.00 | 1.25 | 1.17 | 0.96 | 1.42 | 1.41 | 0.86 | 2.32 |
| Pakistani | 1.41 | 1.21 | 1.64 | 1.28 | 1.14 | 1.44 | 1.16 | 1.02 | 1.32 | 1.54 | 1.29 | 1.84 | 1.70 | 0.95 | 3.05 |
| Bangladeshi | 1.85 | 1.43 | 2.41 | 1.47 | 1.22 | 1.77 | 1.56 | 1.29 | 1.89 | 2.11 | 1.59 | 2.81 | 1.46 | 0.46 | 4.60 |
| Chinese | 1.24 | 0.83 | 1.85 | 2.19 | 1.54 | 3.12 | 1.66 | 1.11 | 2.47 | 2.05 | 1.21 | 3.47 | 0.95 | 0.13 | 6.79 |
| Any other Asian background | 0.70 | 0.56 | 0.87 | 1.14 | 0.94 | 1.36 | 1.07 | 0.90 | 1.27 | 0.87 | 0.63 | 1.19 | 1.38 | 0.67 | 2.80 |
| African | 0.86 | 0.72 | 1.04 | 1.13 | 0.91 | 1.41 | 1.10 | 0.91 | 1.34 | 1.51 | 1.21 | 1.90 | 0.60 | 0.22 | 1.63 |
| Caribbean | 0.88 | 0.76 | 1.02 | 0.95 | 0.79 | 1.13 | 1.09 | 0.95 | 1.26 | 0.73 | 0.61 | 0.88 | 0.58 | 0.31 | 1.09 |
| Any other Black background | 1.55 | 1.18 | 2.04 | 1.63 | 1.19 | 2.24 | 0.85 | 0.59 | 1.22 | 0.84 | 0.54 | 1.29 | 0.58 | 0.14 | 2.35 |
| White and Black Caribbean | 1.59 | 1.23 | 2.06 | 0.71 | 0.44 | 1.14 | 1.56 | 1.14 | 2.13 | 1.66 | 1.17 | 2.35 | 0.44 | 0.06 | 3.14 |
| White and Black African | 1.04 | 0.63 | 1.74 | 0.93 | 0.50 | 1.73 | 1.10 | 0.62 | 1.93 | 0.53 | 0.20 | 1.40 | 1.20 | 0.17 | 8.56 |
| White and Asian | 1.10 | 0.64 | 1.89 | 1.11 | 0.63 | 1.96 | 1.35 | 0.80 | 2.29 | 1.04 | 0.43 | 2.49 | 1.86 | 0.26 | 13.28 |
| Any other Mixed background | 0.74 | 0.47 | 1.16 | 1.18 | 0.80 | 1.75 | 0.89 | 0.58 | 1.36 | 1.58 | 1.03 | 2.43 | 1.23 | 0.30 | 4.95 |
| Arab | 1.74 | 0.83 | 3.66 | 0.61 | 0.20 | 1.89 | 1.21 | 0.50 | 2.91 | 1.35 | 0.51 | 3.61 | 3.32 | 0.46 | 23.88 |
| Any Other Ethnic Group | 1.23 | 1.04 | 1.46 | 1.03 | 0.83 | 1.28 | 1.04 | 0.86 | 1.27 | 1.02 | 0.76 | 1.38 | 1.89 | 0.97 | 3.70 |
| Irish | 0.73 | 0.60 | 0.89 | 0.95 | 0.80 | 1.12 | 0.98 | 0.83 | 1.16 | 0.97 | 0.74 | 1.26 | 0.77 | 0.37 | 1.64 |
| Gypsy or Irish Traveller | 0.54 | 0.08 | 3.86 | 0.41 | 0.06 | 2.90 | 1.39 | 0.62 | 3.10 | 0.63 | 0.09 | 4.44 | 0.00 | 0 | Inf |
| Any other White background | 0.67 | 0.61 | 0.75 | 0.79 | 0.72 | 0.86 | 0.89 | 0.82 | 0.96 | 0.98 | 0.88 | 1.10 | 0.79 | 0.57 | 1.08 |
| Unknown/Not stated | 0.79 | 0.69 | 0.90 | 0.75 | 0.67 | 0.85 | 0.99 | 0.89 | 1.10 | 1.14 | 0.97 | 1.33 | 0.82 | 0.51 | 1.33 |
| Age | 1.30 | 1.27 | 1.32 | 1.37 | 1.34 | 1.39 | 1.32 | 1.30 | 1.34 | 1.20 | 1.18 | 1.23 | 1.27 | 1.19 | 1.34 |
| I(age^2) | 1.00 | 1.00 | 1.00 | 1.00 | 1.00 | 1.00 | 1.00 | 1.00 | 1.00 | 1.00 | 1.00 | 1.00 | 1.00 | 1.00 | 1.00 |
| Pregnancy (yes) | 1.36 | 0.34 | 5.48 | 1.18 | 0.16 | 8.43 | 4.54E-05 | 1.05E-111 | 1.96E+ 102 | 1.60E-05 | 6.50E-161 | 3.95E+ 150 | 0.00 | 0 | Inf |
| Vaccination (yes) | NA | NA | NA | 0.61 | 0.50 | 0.75 | 0.75 | 0.72 | 0.79 | 0.17 | 0.16 | 0.18 | 0.20 | 0.16 | 0.24 |
| IMD quintile: IMD 2 | 0.99 | 0.94 | 1.05 | 0.93 | 0.88 | 0.98 | 0.93 | 0.88 | 0.98 | 0.81 | 0.75 | 0.88 | 0.83 | 0.68 | 1.02 |
| IMD quintile: IMD 3 | 0.96 | 0.91 | 1.02 | 0.86 | 0.81 | 0.90 | 0.86 | 0.81 | 0.91 | 0.77 | 0.71 | 0.84 | 0.69 | 0.56 | 0.86 |
| IMD quintile: IMD 4 | 0.94 | 0.89 | 1.00 | 0.82 | 0.78 | 0.87 | 0.81 | 0.77 | 0.86 | 0.69 | 0.63 | 0.75 | 0.60 | 0.48 | 0.75 |
| IMD quintile: IMD 5 (less deprived) | 0.93 | 0.87 | 0.99 | 0.78 | 0.74 | 0.82 | 0.82 | 0.78 | 0.87 | 0.67 | 0.61 | 0.74 | 0.55 | 0.43 | 0.70 |
| IMD quintile: IMD Unknown | 0.76 | 0.34 | 1.70 | 0.72 | 0.34 | 1.51 | 0.95 | 0.48 | 1.92 | 0.64 | 0.21 | 2.00 | 0.00 | 0 | Inf |
| LSOA region: East of England | 1.13 | 1.02 | 1.25 | 1.12 | 1.03 | 1.22 | 1.04 | 0.96 | 1.12 | 0.89 | 0.77 | 1.03 | 1.22 | 0.85 | 1.74 |
| LSOA region: London | 0.98 | 0.90 | 1.08 | 1.03 | 0.95 | 1.12 | 0.88 | 0.81 | 0.95 | 0.94 | 0.83 | 1.07 | 1.08 | 0.77 | 1.52 |
| LSOA region: North East | 1.13 | 1.02 | 1.26 | 0.93 | 0.85 | 1.03 | 0.86 | 0.76 | 0.96 | 0.99 | 0.85 | 1.15 | 1.04 | 0.72 | 1.52 |
| LSOA region: North West | 1.01 | 0.93 | 1.10 | 1.03 | 0.96 | 1.11 | 0.93 | 0.86 | 1.00 | 0.99 | 0.88 | 1.11 | 1.01 | 0.74 | 1.38 |
| LSOA region: South East | 0.98 | 0.89 | 1.07 | 1.10 | 1.02 | 1.19 | 0.97 | 0.90 | 1.05 | 0.91 | 0.80 | 1.04 | 0.91 | 0.64 | 1.29 |
| LSOA region: South West | 0.87 | 0.77 | 0.98 | 0.94 | 0.85 | 1.04 | 0.81 | 0.73 | 0.89 | 0.86 | 0.75 | 0.99 | 1.05 | 0.72 | 1.54 |
| LSOA region: West Midlands | 0.94 | 0.86 | 1.03 | 0.98 | 0.91 | 1.07 | 0.92 | 0.85 | 1.00 | 0.89 | 0.79 | 1.02 | 0.90 | 0.63 | 1.27 |
| LSOA region: Yorkshire and The Humber | 1.12 | 1.02 | 1.23 | 1.03 | 0.95 | 1.11 | 0.95 | 0.87 | 1.04 | 1.01 | 0.90 | 1.14 | 0.78 | 0.55 | 1.12 |
| LSOA region: Unknown | 1.08 | 0.98 | 1.19 | 1.02 | 0.94 | 1.11 | 0.92 | 0.85 | 1.00 | 0.92 | 0.81 | 1.05 | 1.03 | 0.73 | 1.46 |
| Atrial Fibrillation (yes) | 1.03 | 0.99 | 1.08 | 1.18 | 1.13 | 1.23 | 1.11 | 1.06 | 1.17 | 1.42 | 1.32 | 1.52 | 1.49 | 1.25 | 1.78 |
| Obesity (yes) | 1.07 | 1.01 | 1.14 | 1.13 | 1.07 | 1.19 | 1.12 | 1.06 | 1.18 | 1.25 | 1.16 | 1.34 | 1.09 | 0.87 | 1.37 |
| Chronic Kidney Disease (yes) | 1.20 | 1.16 | 1.25 | 1.36 | 1.31 | 1.41 | 1.37 | 1.31 | 1.42 | 1.64 | 1.54 | 1.75 | 1.38 | 1.18 | 1.61 |
| Diabetes (yes) | 1.23 | 1.18 | 1.29 | 1.35 | 1.29 | 1.40 | 1.37 | 1.31 | 1.43 | 1.70 | 1.59 | 1.81 | 1.30 | 1.09 | 1.53 |
| Chronic mental health disorders (yes) | 1.13 | 1.04 | 1.22 | 1.24 | 1.15 | 1.34 | 1.19 | 1.10 | 1.28 | 1.50 | 1.34 | 1.68 | 0.86 | 0.58 | 1.26 |
| Use of CVD prevention medication (yes) | 1.03 | 0.99 | 1.08 | 1.15 | 1.11 | 1.20 | 1.08 | 1.03 | 1.12 | 1.20 | 1.12 | 1.28 | 1.15 | 0.98 | 1.36 |
| Rheumatoid Arthritis (yes) | 1.10 | 1.00 | 1.21 | 1.43 | 1.32 | 1.56 | 1.36 | 1.25 | 1.49 | 2.37 | 2.13 | 2.64 | 2.18 | 1.63 | 2.90 |
| Antipsychotic (yes) | 1.21 | 1.07 | 1.37 | 1.24 | 1.09 | 1.40 | 1.20 | 1.06 | 1.35 | 1.29 | 1.01 | 1.66 | 1.63 | 1.00 | 2.67 |
| Cancer (yes) | 1.12 | 1.08 | 1.18 | 1.09 | 1.05 | 1.14 | 1.13 | 1.09 | 1.18 | 1.17 | 1.10 | 1.24 | 1.38 | 1.18 | 1.62 |
| Chronic obstructive pulmonary disease (yes) | 1.20 | 1.14 | 1.27 | 1.75 | 1.67 | 1.84 | 1.72 | 1.63 | 1.81 | 2.33 | 2.17 | 2.50 | 2.47 | 2.06 | 2.95 |
| Dementia (yes) | 1.24 | 1.18 | 1.29 | 1.25 | 1.20 | 1.30 | 1.28 | 1.23 | 1.33 | 1.42 | 1.32 | 1.54 | 1.15 | 0.97 | 1.36 |
| Hypertension (yes) | 0.98 | 0.94 | 1.02 | 1.02 | 0.98 | 1.06 | 1.02 | 0.98 | 1.06 | 1.19 | 1.11 | 1.27 | 0.97 | 0.83 | 1.14 |
| **Reference group**s: White British for ethnicity; IMD 1 (most deprived) for IMD quintile; East Midlands for English region.  **Abbreviations**: CI, confidence interval; CVD, cardiovascular disease; IMD, index of multiple deprivation; I(age^2), main effect and the second order interaction of age. | | | | | | | | | | | | | | | |

Supplementary Table 16. Adjusted hazard ratios of 30-day CVD from a) men and b) women diagnosed with COVID-19 with diverse ethnic background by the period of COVID-19 diagnosis in England, using White British women as reference group. Period of COVID-19 diagnosis is determined by date of COVID-19 diagnosis. Ethnic classification used: NHS ethnicity codes.

#### a) Men estimates

| **Time of COVID-19 diagnosis** | **23 Jan 2020 to 30 Jun 2020** | | | **01 Jul 2020 to 31 Dec 2020** | | | **01 Jan 2021 to 30 Jun 2021** | | | **01 Jul 2021 to 31 Dec 2021** | | | **01 Jan 2022 to 01 Apr 2022** | | |
| --- | --- | --- | --- | --- | --- | --- | --- | --- | --- | --- | --- | --- | --- | --- | --- |
| **Variables** | **HR** | **Low 95% CI** | **High 95% CI** | **HR** | **Low 95% CI** | **High 95% CI** | **HR** | **Low 95% CI** | **High 95% CI** | **HR** | **Low 95% CI** | **High 95% CI** | **HR** | **Low 95% CI** | **High 95% CI** |
| Indian | 0.94 | 0.81 | 1.08 | 0.99 | 0.90 | 1.09 | 0.95 | 0.86 | 1.04 | 1.05 | 0.94 | 1.17 | 0.90 | 0.74 | 1.10 |
| Pakistani | 1.17 | 0.99 | 1.38 | 1.30 | 1.19 | 1.42 | 1.32 | 1.20 | 1.45 | 1.51 | 1.35 | 1.68 | 1.58 | 1.32 | 1.90 |
| Bangladeshi | 1.03 | 0.74 | 1.44 | 1.31 | 1.13 | 1.52 | 1.23 | 1.05 | 1.44 | 1.42 | 1.17 | 1.74 | 1.19 | 0.82 | 1.72 |
| Chinese | 1.41 | 0.86 | 2.31 | 1.46 | 0.97 | 2.19 | 1.43 | 0.99 | 2.05 | 1.43 | 0.98 | 2.09 | 0.53 | 0.20 | 1.40 |
| Any other Asian background | 0.93 | 0.77 | 1.14 | 1.26 | 1.10 | 1.44 | 1.18 | 1.04 | 1.34 | 1.16 | 0.99 | 1.36 | 0.83 | 0.63 | 1.11 |
| African | 0.62 | 0.50 | 0.79 | 0.95 | 0.79 | 1.15 | 0.90 | 0.76 | 1.06 | 0.94 | 0.77 | 1.13 | 1.07 | 0.80 | 1.41 |
| Caribbean | 0.76 | 0.62 | 0.93 | 0.98 | 0.82 | 1.18 | 1.12 | 0.96 | 1.30 | 1.01 | 0.87 | 1.17 | 1.16 | 0.89 | 1.51 |
| Any other Black background | 1.01 | 0.70 | 1.45 | 0.90 | 0.62 | 1.29 | 1.17 | 0.89 | 1.53 | 1.11 | 0.85 | 1.45 | 0.97 | 0.61 | 1.54 |
| White and Black Caribbean | 0.90 | 0.55 | 1.47 | 1.13 | 0.75 | 1.72 | 0.87 | 0.58 | 1.31 | 1.27 | 0.92 | 1.75 | 0.98 | 0.49 | 1.96 |
| White and Black African | 0.88 | 0.47 | 1.64 | 0.69 | 0.37 | 1.28 | 0.78 | 0.47 | 1.30 | 0.57 | 0.28 | 1.13 | 1.31 | 0.62 | 2.75 |
| White and Asian | 1.40 | 0.77 | 2.53 | 1.52 | 1.02 | 2.27 | 0.95 | 0.57 | 1.57 | 1.20 | 0.77 | 1.89 | 2.20 | 1.28 | 3.80 |
| Any other Mixed background | 1.18 | 0.73 | 1.90 | 1.10 | 0.79 | 1.55 | 1.18 | 0.85 | 1.64 | 1.02 | 0.71 | 1.46 | 0.76 | 0.38 | 1.52 |
| Arab | 1.52 | 0.76 | 3.05 | 1.40 | 0.88 | 2.23 | 1.28 | 0.78 | 2.10 | 1.35 | 0.78 | 2.32 | 2.14 | 0.96 | 4.78 |
| Any Other Ethnic Group | 1.07 | 0.85 | 1.34 | 1.25 | 1.06 | 1.48 | 1.25 | 1.06 | 1.46 | 1.47 | 1.24 | 1.75 | 1.15 | 0.84 | 1.56 |
| Irish | 0.84 | 0.65 | 1.09 | 1.15 | 0.98 | 1.36 | 0.98 | 0.82 | 1.17 | 1.08 | 0.91 | 1.28 | 1.13 | 0.85 | 1.50 |
| Gypsy or Irish Traveller | 0.92 | 0.29 | 2.84 | 0.89 | 0.33 | 2.38 | 1.17 | 0.52 | 2.60 | 0.40 | 0.10 | 1.60 | 0.86 | 0.21 | 3.45 |
| Any other White background | 0.94 | 0.84 | 1.07 | 1.06 | 0.98 | 1.14 | 1.11 | 1.03 | 1.19 | 1.04 | 0.97 | 1.12 | 1.17 | 1.04 | 1.31 |
| Unknown/Not stated | 0.85 | 0.71 | 1.02 | 0.81 | 0.72 | 0.92 | 0.95 | 0.84 | 1.07 | 0.84 | 0.75 | 0.94 | 0.81 | 0.66 | 0.98 |
| Age | 1.16 | 1.14 | 1.19 | 1.20 | 1.19 | 1.22 | 1.20 | 1.19 | 1.22 | 1.12 | 1.11 | 1.14 | 1.11 | 1.09 | 1.13 |
| I(age^2) | 1.00 | 1.00 | 1.00 | 1.00 | 1.00 | 1.00 | 1.00 | 1.00 | 1.00 | 1.00 | 1.00 | 1.00 | 1.00 | 1.00 | 1.00 |
| Vaccination (yes) | NA | NA | NA | 1.25 | 1.03 | 1.52 | 1.12 | 1.06 | 1.18 | 0.32 | 0.30 | 0.34 | 0.43 | 0.38 | 0.48 |
| IMD quintile: IMD 2 | 1.05 | 0.97 | 1.14 | 0.97 | 0.93 | 1.02 | 0.86 | 0.81 | 0.90 | 0.90 | 0.86 | 0.95 | 0.85 | 0.78 | 0.93 |
| IMD quintile: IMD 3 | 1.01 | 0.93 | 1.10 | 0.89 | 0.84 | 0.94 | 0.84 | 0.80 | 0.89 | 0.80 | 0.76 | 0.84 | 0.78 | 0.71 | 0.85 |
| IMD quintile: IMD 4 | 0.95 | 0.88 | 1.04 | 0.89 | 0.84 | 0.94 | 0.80 | 0.76 | 0.85 | 0.74 | 0.70 | 0.78 | 0.73 | 0.67 | 0.80 |
| IMD quintile: IMD 5 (less deprived) | 0.91 | 0.83 | 1.00 | 0.88 | 0.83 | 0.93 | 0.77 | 0.72 | 0.82 | 0.69 | 0.65 | 0.73 | 0.66 | 0.60 | 0.72 |
| IMD quintile: IMD Unknown | 1.13 | 0.42 | 3.03 | 0.54 | 0.18 | 1.69 | 1.14 | 0.57 | 2.28 | 1.91 | 1.02 | 3.57 | 1.06 | 0.34 | 3.30 |
| LSOA region: East of England | 0.90 | 0.79 | 1.03 | 0.96 | 0.87 | 1.05 | 0.97 | 0.88 | 1.06 | 0.96 | 0.87 | 1.05 | 0.91 | 0.78 | 1.06 |
| LSOA region: London | 0.88 | 0.78 | 1.00 | 1.06 | 0.98 | 1.15 | 0.95 | 0.87 | 1.04 | 1.02 | 0.94 | 1.11 | 1.09 | 0.94 | 1.25 |
| LSOA region: North East | 0.54 | 0.45 | 0.65 | 0.91 | 0.83 | 1.01 | 0.87 | 0.77 | 0.99 | 0.97 | 0.88 | 1.07 | 1.00 | 0.86 | 1.17 |
| LSOA region: North West | 0.80 | 0.71 | 0.90 | 1.11 | 1.04 | 1.20 | 1.01 | 0.93 | 1.10 | 1.14 | 1.06 | 1.23 | 1.12 | 0.99 | 1.27 |
| LSOA region: South East | 0.73 | 0.64 | 0.83 | 1.08 | 1.00 | 1.17 | 0.99 | 0.91 | 1.08 | 1.08 | 1.00 | 1.17 | 1.03 | 0.90 | 1.18 |
| LSOA region: South West | 0.69 | 0.58 | 0.82 | 1.04 | 0.94 | 1.16 | 1.04 | 0.94 | 1.16 | 1.02 | 0.94 | 1.12 | 0.96 | 0.82 | 1.12 |
| LSOA region: West Midlands | 0.91 | 0.81 | 1.03 | 1.11 | 1.03 | 1.20 | 1.03 | 0.95 | 1.13 | 1.14 | 1.06 | 1.24 | 1.01 | 0.88 | 1.16 |
| LSOA region: Yorkshire and The Humber | 0.99 | 0.87 | 1.12 | 0.94 | 0.86 | 1.02 | 0.99 | 0.90 | 1.09 | 1.02 | 0.94 | 1.10 | 0.96 | 0.83 | 1.10 |
| LSOA region: Unknown | 0.82 | 0.71 | 0.93 | 0.97 | 0.89 | 1.06 | 0.98 | 0.89 | 1.07 | 0.98 | 0.90 | 1.07 | 0.99 | 0.86 | 1.14 |
| Atrial Fibrillation (yes) | 1.29 | 1.21 | 1.37 | 1.33 | 1.27 | 1.38 | 1.34 | 1.28 | 1.41 | 1.83 | 1.76 | 1.91 | 1.99 | 1.86 | 2.13 |
| Obesity (yes) | 1.07 | 0.99 | 1.16 | 1.14 | 1.08 | 1.19 | 1.10 | 1.04 | 1.16 | 1.15 | 1.10 | 1.20 | 1.06 | 0.97 | 1.15 |
| Chronic Kidney Disease (yes) | 1.36 | 1.28 | 1.44 | 1.54 | 1.48 | 1.61 | 1.53 | 1.46 | 1.60 | 1.74 | 1.67 | 1.81 | 1.71 | 1.59 | 1.83 |
| Diabetes (yes) | 1.14 | 1.08 | 1.21 | 1.21 | 1.16 | 1.25 | 1.25 | 1.20 | 1.30 | 1.34 | 1.29 | 1.39 | 1.18 | 1.10 | 1.25 |
| Chronic mental health disorders (yes) | 0.93 | 0.81 | 1.06 | 1.17 | 1.06 | 1.28 | 1.12 | 1.03 | 1.23 | 1.21 | 1.10 | 1.33 | 1.17 | 1.01 | 1.36 |
| Use of CVD prevention medication (yes) | 2.29 | 2.12 | 2.48 | 2.99 | 2.83 | 3.15 | 3.02 | 2.86 | 3.19 | 3.70 | 3.50 | 3.90 | 3.85 | 3.51 | 4.22 |
| Rheumatoid Arthritis (yes) | 1.00 | 0.82 | 1.21 | 1.12 | 0.99 | 1.28 | 1.21 | 1.06 | 1.38 | 1.51 | 1.35 | 1.69 | 1.48 | 1.22 | 1.79 |
| Antipsychotic (yes) | 0.87 | 0.68 | 1.11 | 0.93 | 0.76 | 1.12 | 0.89 | 0.73 | 1.09 | 1.03 | 0.79 | 1.34 | 1.15 | 0.85 | 1.55 |
| Cancer (yes) | 0.90 | 0.84 | 0.97 | 1.09 | 1.04 | 1.14 | 1.10 | 1.05 | 1.16 | 1.27 | 1.21 | 1.32 | 1.37 | 1.27 | 1.47 |
| Chronic obstructive pulmonary disease (yes) | 1.29 | 1.20 | 1.39 | 1.51 | 1.44 | 1.59 | 1.53 | 1.45 | 1.61 | 1.85 | 1.77 | 1.94 | 1.66 | 1.53 | 1.80 |
| Dementia (yes) | 0.72 | 0.66 | 0.78 | 0.79 | 0.74 | 0.84 | 0.75 | 0.70 | 0.81 | 1.04 | 0.96 | 1.13 | 1.10 | 0.99 | 1.22 |
| Hypertension (yes) | 1.09 | 1.03 | 1.16 | 1.02 | 0.98 | 1.06 | 1.00 | 0.96 | 1.04 | 1.02 | 0.98 | 1.06 | 0.99 | 0.93 | 1.05 |
| **Reference group**s: White British for ethnicity; IMD 1 (most deprived) for IMD quintile; East Midlands for English region. **Abbreviations**: CI, confidence interval; CVD, cardiovascular disease; IMD, index of multiple deprivation; I(age^2), main effect and the second order interaction of age. | | | | | | | | | | | | | | | |

#### b) Women estimates

| **Time of COVID-19 diagnosis** | **23 Jan 2020 to 30 Jun 2020** | | | **01 Jul 2020 to 31 Dec 2020** | | | **01 Jan 2021 to 30 Jun 2021** | | | **01 Jul 2021 to 31 Dec 2021** | | | **01 Jan 2022 to 01 Apr 2022** | | |
| --- | --- | --- | --- | --- | --- | --- | --- | --- | --- | --- | --- | --- | --- | --- | --- |
| **Variables** | **HR** | **Low 95% CI** | **High 95% CI** | **HR** | **Low 95% CI** | **High 95% CI** | **HR** | **Low 95% CI** | **High 95% CI** | **HR** | **Low 95% CI** | **High 95% CI** | **HR** | **Low 95% CI** | **High 95% CI** |
| Indian | 1.13 | 0.91 | 1.39 | 1.02 | 0.89 | 1.16 | 0.98 | 0.86 | 1.13 | 0.92 | 0.78 | 1.08 | 1.08 | 0.84 | 1.39 |
| Pakistani | 1.23 | 0.97 | 1.56 | 1.07 | 0.95 | 1.22 | 1.07 | 0.93 | 1.23 | 1.37 | 1.17 | 1.59 | 1.29 | 0.98 | 1.69 |
| Bangladeshi | 1.86 | 1.23 | 2.80 | 0.91 | 0.72 | 1.14 | 1.09 | 0.86 | 1.36 | 0.97 | 0.72 | 1.32 | 1.75 | 1.13 | 2.72 |
| Chinese | 0.65 | 0.24 | 1.73 | 1.88 | 1.16 | 3.02 | 1.20 | 0.68 | 2.11 | 1.38 | 0.83 | 2.29 | 0.45 | 0.11 | 1.82 |
| Any other Asian background | 1.16 | 0.88 | 1.52 | 1.01 | 0.83 | 1.23 | 0.92 | 0.76 | 1.12 | 1.05 | 0.84 | 1.30 | 1.12 | 0.79 | 1.57 |
| African | 1.00 | 0.75 | 1.33 | 1.03 | 0.81 | 1.30 | 1.04 | 0.84 | 1.28 | 1.10 | 0.89 | 1.36 | 1.36 | 0.98 | 1.89 |
| Caribbean | 1.07 | 0.83 | 1.37 | 1.09 | 0.89 | 1.34 | 1.21 | 1.02 | 1.43 | 0.95 | 0.80 | 1.13 | 1.55 | 1.19 | 2.02 |
| Any other Black background | 1.12 | 0.65 | 1.95 | 1.19 | 0.80 | 1.78 | 1.05 | 0.72 | 1.52 | 1.07 | 0.77 | 1.50 | 1.07 | 0.57 | 2.00 |
| White and Black Caribbean | 2.46 | 1.63 | 3.73 | 1.54 | 1.04 | 2.28 | 1.03 | 0.65 | 1.63 | 1.16 | 0.79 | 1.70 | 0.98 | 0.47 | 2.06 |
| White and Black African | 2.30 | 1.30 | 4.06 | 0.56 | 0.25 | 1.25 | 1.35 | 0.79 | 2.34 | 0.59 | 0.26 | 1.32 | 0.25 | 0.04 | 1.78 |
| White and Asian | 1.41 | 0.63 | 3.15 | 1.62 | 0.96 | 2.73 | 1.29 | 0.71 | 2.33 | 0.80 | 0.38 | 1.67 | 0.97 | 0.31 | 3.01 |
| Any other Mixed background | 1.23 | 0.68 | 2.24 | 1.21 | 0.79 | 1.87 | 0.75 | 0.44 | 1.27 | 1.08 | 0.72 | 1.63 | 1.55 | 0.85 | 2.80 |
| Arab | 3.80 | 1.42 | 10.18 | 0.87 | 0.33 | 2.32 | 1.45 | 0.65 | 3.24 | 2.07 | 1.08 | 3.99 | 2.61 | 0.84 | 8.12 |
| Any Other Ethnic Group | 1.24 | 0.91 | 1.69 | 1.22 | 0.97 | 1.53 | 1.04 | 0.82 | 1.32 | 1.02 | 0.79 | 1.31 | 1.82 | 1.31 | 2.53 |
| Irish | 0.88 | 0.62 | 1.26 | 1.01 | 0.81 | 1.25 | 1.06 | 0.85 | 1.32 | 1.04 | 0.83 | 1.29 | 1.11 | 0.79 | 1.57 |
| Gypsy or Irish Traveller | 2.64 | 0.66 | 10.58 | 1.95 | 0.73 | 5.20 | 1.39 | 0.52 | 3.69 | 1.48 | 0.61 | 3.55 | 1.30 | 0.18 | 9.23 |
| Any other White background | 0.92 | 0.79 | 1.08 | 1.00 | 0.91 | 1.10 | 0.98 | 0.89 | 1.07 | 1.03 | 0.94 | 1.12 | 1.09 | 0.95 | 1.26 |
| Unknown/Not stated | 0.91 | 0.73 | 1.13 | 0.84 | 0.72 | 0.97 | 0.99 | 0.86 | 1.14 | 0.96 | 0.83 | 1.11 | 0.94 | 0.75 | 1.19 |
| Age | 1.22 | 1.19 | 1.25 | 1.23 | 1.21 | 1.24 | 1.20 | 1.18 | 1.22 | 1.11 | 1.09 | 1.12 | 1.13 | 1.10 | 1.15 |
| I(age^2) | 1.00 | 1.00 | 1.00 | 1.00 | 1.00 | 1.00 | 1.00 | 1.00 | 1.00 | 1.00 | 1.00 | 1.00 | 1.00 | 1.00 | 1.00 |
| Pregnancy (yes) | 1.99 | 0.63 | 6.32 | 3.02 | 1.65 | 5.55 | 3.05 | 1.78 | 5.23 | 2.99 | 2.01 | 4.45 | 2.79E-05 | 1.30E-300 | 5.99E+ 290 |
| Vaccination (yes) | NA | NA | NA | 1.00 | 0.78 | 1.27 | 0.99 | 0.93 | 1.05 | 0.38 | 0.36 | 0.41 | 0.50 | 0.43 | 0.57 |
| IMD quintile: IMD 2 | 1.05 | 0.96 | 1.16 | 0.94 | 0.89 | 1.00 | 0.94 | 0.88 | 1.01 | 0.85 | 0.79 | 0.90 | 0.93 | 0.84 | 1.03 |
| IMD quintile: IMD 3 | 0.97 | 0.88 | 1.08 | 0.86 | 0.81 | 0.92 | 0.90 | 0.84 | 0.96 | 0.82 | 0.77 | 0.88 | 0.87 | 0.78 | 0.97 |
| IMD quintile: IMD 4 | 1.00 | 0.90 | 1.11 | 0.83 | 0.77 | 0.89 | 0.92 | 0.86 | 0.99 | 0.74 | 0.69 | 0.79 | 0.78 | 0.70 | 0.87 |
| IMD quintile: IMD 5 (less deprived) | 0.93 | 0.83 | 1.04 | 0.84 | 0.78 | 0.90 | 0.87 | 0.80 | 0.94 | 0.67 | 0.62 | 0.73 | 0.70 | 0.62 | 0.79 |
| IMD quintile: IMD Unknown | 8.73E-06 | 9.93E-206 | 7.68E+  194 | 1.89 | 0.94 | 3.80 | 1.91 | 0.95 | 3.84 | 1.31 | 0.58 | 2.92 | 2.45 | 0.91 | 6.60 |
| LSOA region: East of England | 0.91 | 0.77 | 1.08 | 1.09 | 0.97 | 1.23 | 0.89 | 0.80 | 1.00 | 0.91 | 0.81 | 1.03 | 0.87 | 0.72 | 1.06 |
| LSOA region: London | 0.79 | 0.68 | 0.92 | 1.27 | 1.14 | 1.41 | 0.88 | 0.79 | 0.99 | 1.08 | 0.96 | 1.20 | 1.01 | 0.85 | 1.21 |
| LSOA region: North East | 0.51 | 0.41 | 0.64 | 0.94 | 0.83 | 1.07 | 0.86 | 0.74 | 0.99 | 1.07 | 0.95 | 1.22 | 1.12 | 0.93 | 1.35 |
| LSOA region: North West | 0.83 | 0.72 | 0.96 | 1.22 | 1.11 | 1.34 | 1.12 | 1.01 | 1.24 | 1.29 | 1.17 | 1.42 | 1.23 | 1.05 | 1.43 |
| LSOA region: South East | 0.72 | 0.62 | 0.84 | 1.18 | 1.06 | 1.31 | 1.04 | 0.94 | 1.15 | 1.08 | 0.97 | 1.20 | 0.97 | 0.82 | 1.16 |
| LSOA region: South West | 0.67 | 0.54 | 0.82 | 1.10 | 0.96 | 1.26 | 0.89 | 0.78 | 1.02 | 1.01 | 0.90 | 1.14 | 0.92 | 0.75 | 1.12 |
| LSOA region: Unknown | 0.75 | 0.63 | 0.88 | 1.11 | 0.99 | 1.24 | 0.96 | 0.86 | 1.07 | 1.00 | 0.89 | 1.12 | 0.97 | 0.81 | 1.16 |
| LSOA region: West Midlands | 0.77 | 0.67 | 0.90 | 1.18 | 1.07 | 1.31 | 1.01 | 0.90 | 1.12 | 1.15 | 1.03 | 1.28 | 1.08 | 0.91 | 1.28 |
| LSOA region: Yorkshire and The Humber | 1.03 | 0.88 | 1.20 | 1.09 | 0.98 | 1.21 | 1.03 | 0.92 | 1.16 | 1.00 | 0.90 | 1.12 | 0.98 | 0.83 | 1.17 |
| Atrial Fibrillation (yes) | 1.46 | 1.34 | 1.58 | 1.54 | 1.46 | 1.63 | 1.52 | 1.44 | 1.61 | 2.10 | 1.99 | 2.23 | 2.45 | 2.24 | 2.67 |
| Obesity (yes) | 1.04 | 0.94 | 1.15 | 1.19 | 1.12 | 1.27 | 1.13 | 1.06 | 1.20 | 1.16 | 1.09 | 1.23 | 1.08 | 0.98 | 1.20 |
| Chronic Kidney Disease (yes) | 1.33 | 1.23 | 1.43 | 1.47 | 1.40 | 1.55 | 1.49 | 1.42 | 1.57 | 1.69 | 1.60 | 1.78 | 1.53 | 1.41 | 1.67 |
| Diabetes (yes) | 1.22 | 1.13 | 1.31 | 1.28 | 1.22 | 1.34 | 1.29 | 1.23 | 1.36 | 1.36 | 1.29 | 1.43 | 1.28 | 1.18 | 1.39 |
| Chronic mental health disorders (yes) | 0.90 | 0.78 | 1.04 | 1.09 | 0.99 | 1.20 | 1.12 | 1.01 | 1.23 | 1.21 | 1.10 | 1.34 | 1.11 | 0.94 | 1.30 |
| Use of CVD prevention medication (yes) | 2.42 | 2.20 | 2.66 | 3.02 | 2.84 | 3.22 | 2.97 | 2.79 | 3.16 | 4.05 | 3.79 | 4.32 | 3.79 | 3.42 | 4.21 |
| Rheumatoid Arthritis (yes) | 1.01 | 0.85 | 1.20 | 1.43 | 1.29 | 1.59 | 1.21 | 1.08 | 1.37 | 1.47 | 1.32 | 1.64 | 1.36 | 1.13 | 1.63 |
| Antipsychotic (yes) | 1.05 | 0.80 | 1.38 | 0.89 | 0.70 | 1.12 | 0.85 | 0.67 | 1.08 | 1.16 | 0.88 | 1.53 | 1.24 | 0.87 | 1.77 |
| Cancer (yes) | 0.92 | 0.85 | 1.00 | 0.94 | 0.90 | 0.99 | 0.90 | 0.85 | 0.94 | 0.96 | 0.92 | 1.01 | 0.96 | 0.89 | 1.04 |
| Chronic obstructive pulmonary disease (yes) | 1.45 | 1.33 | 1.59 | 1.75 | 1.65 | 1.86 | 1.70 | 1.59 | 1.81 | 2.14 | 2.02 | 2.28 | 2.00 | 1.81 | 2.21 |
| Dementia (yes) | 0.59 | 0.53 | 0.64 | 0.67 | 0.63 | 0.72 | 0.62 | 0.58 | 0.67 | 0.93 | 0.85 | 1.01 | 0.77 | 0.69 | 0.87 |
| Hypertension (yes) | 1.23 | 1.14 | 1.33 | 1.21 | 1.15 | 1.28 | 1.17 | 1.11 | 1.24 | 1.19 | 1.12 | 1.25 | 1.17 | 1.08 | 1.28 |
| **Reference group**s: White British for ethnicity; IMD 1 (most deprived) for IMD quintile; East Midlands for English region.  **Abbreviations**: CI, confidence interval; CVD, cardiovascular disease; IMD, index of multiple deprivation; I(age^2), main effect and the second order interaction of age. | | | | | | | | | | | | | | | |
